# Promoting the Adsorption of Metal Ions on Kaolinite by Defect Sites: A Molecular Dynamics Study

**DOI:** 10.1038/srep14377

**Published:** 2015-09-25

**Authors:** Xiong Li, Hang Li, Gang Yang

**Affiliations:** 1 College of Resources and Environment & Chongqing Key Laboratory of Soil Multi-scale Interfacial Process, Southwest University, Chongqing 400715, China

## Abstract

Defect sites exist abundantly in minerals and play a crucial role for a variety of important processes. Here molecular dynamics simulations are used to comprehensively investigate the adsorption behaviors, stabilities and mechanisms of metal ions on defective minerals, considering different ionic concentrations, defect sizes and contents. Outer-sphere adsorbed Pb^2+^ ions predominate for all models (regular and defective), while inner-sphere Na^+^ ions, which exist sporadically only at concentrated solutions for regular models, govern the adsorption for all defective models. Adsorption quantities and stabilities of metal ions on kaolinite are fundamentally promoted by defect sites, thus explaining the experimental observations. Defect sites improve the stabilities of both inner- and outer-sphere adsorption, and (quasi) inner-sphere Pb^2+^ ions emerge only at defect sites that reinforce the interactions. Adsorption configurations are greatly altered by defect sites but respond weakly by changing defect sizes or contents. Both adsorption quantities and stabilities are enhanced by increasing defect sizes or contents, while ionic concentrations mainly affect adsorption quantities. We also find that adsorption of metal ions and anions can be promoted by each other and proceeds in a collaborative mechanism. Results thus obtained are beneficial to comprehend related processes for all types of minerals.

Clays are naturally occurring aluminosilicate minerals that have been used in a wide range of catalytic^1^, environmental^2^ and pharmaceutical^3^ processes. Clay minerals have particular ion-exchange capacity and are known as reservoirs for metal ions, while the uptake of heavy metal ions poses serious environmental problems. Currently, the adsorption mechanisms of metal ions on clay minerals remain largely elusive. Two metal ions may correspond to different adsorption behaviors, and the adsorption performances are closely associated with the surface properties of clay minerals that can be greatly altered by the extent of structural defects^2^,^4^,^5^,^6^.

Two adsorption modes (inner- and outer-sphere) were proposed for the interaction of metal ions with clay minerals^7^,^8^,^9^,^10^,^11^,^12^,^13^,^14^,^15^,^16^,^17^,^18^,^19^,^20^,^21^,^22^,^23^,^24^,^25^,^26^,^27^. Inner-sphere metal ions form stable complexes directly with clay minerals, while outer-sphere ones are separated from clay minerals by an intermediate water molecule. The combination of AFM experiments and MD simulations demonstrated that hydrated Na^+^ ions are adsorbed close to calcite as Rb^+^ ions^13^. With use of MD simulations, Greathouse and Cygan^24^ found that [UO_2_(H_2_O)_5_]^2+^ is the principal species for UO_2_^2+^ adsorption on beidellite and montmorillonite, and Yang *et al.*^25^ further pointed out that [UO_2_(H_2_O)_5_]^2+^ can interact with montmorillonite via the U cationic center and O atom of hydration shell corresponding to the inner- and outer-sphere modes, respectively. Sakuma *et al.*^26^ reported that all Na^+^, K^+^ and Cs^+^ ions form stable inner-sphere complexes with muscovite, while Vasconcelos *et al.*^27^ insisted that relatively weak outer-sphere complexes are the main and sole products respectively for Na^+^ and Pb^2+^ adsorption on kaolinite. This seems to contradict with the experimental observations where strong adsorption on kaolinite was documented^2^,^28^,^29^,^30^.

Defect sites, which have been considered by not a few researchers as adsorption and catalytic centers in aluminosilicates and analogues^5^,^6^,^31^,^32^,^33^,^34^,^35^,^36^, were assumed to reconcile such contradictions. Defects in silicates and zeolites (analogues of kaolinite and montmorillonite) have been well documented experimentally, observing different sizes of defects with silanol nests^37^,^38^,^39^,^40^,^41^,^42^,^43^. Silanol defects were also detected in clay minerals^44^,^45^,^46^,^47^, and it has recently been found that a significant fraction of carbon resources reside at the defects of minerals in the Earth’s mantle^5^. Presently, MD simulations were used to simulate the adsorption of metal ions on regular and defective kaolinite (Fig. 1) with aim to demonstrate the roles of defect sites played during such adsorption processes. The adsorption configurations, quantities and stabilities are all fundamentally altered by defect sites, and the computational results can explain the experimental observations^2^,^28^,^29^,^30^. Two metal ions (Na^+^ and Pb^2+^) were chosen that respond distinctly to defect sites. Na^+^ is a common background metal ion while the environmental pollution of Pb^2+^ has emerged as a global concern. The choice of a wide range of ionic concentrations allows us to discern the concentration-dependent adsorption processes and potentially different adsorption behaviors before and after saturation. In order to comprehensively understand defect sites, effects of defect sizes and defect contents were also considered. In the end, the adsorption mechanisms of metal ions and anions on kaolinite were explored.

## Results

### Regular kaolinite

Discussions of Na^+^ and Pb^2+^ adsorption on regular kaolinite are limited in the report of Vasconcelos *et al.*^27^ and will be elaborated here, mainly to facilitate the understanding of the adsorption processes on defect sites. As indicated in Fig. 2, S13 and S14, metal ions (Na^+^/Pb^2+^) and anions (Cl^−^) tend to approach the slightly negatively charged tetrahedral SiO_4_ surfaces and positively charged octahedral AlO_6_ surfaces, respectively; meanwhile, very few Na^+^ ions are driven by Cl^−^ ions towards the octahedral AlO_6_ surfaces, which are in line with the previous results^27^. Ion pairs (Na^+^-Cl^−^ and Pb^2+^-Cl^−^) are detected in bulk solutions that are more apparent with increase of ionic concentrations. Even at low ionic concentrations (0.16 mol/L), a portion of Cl^−^ ions are strongly adsorbed on the octahedral AlO_6_ surface as inner-sphere mode. In contrast, at 0.16 mo/L, all Na^+^ and Pb^2+^ ions are relatively far from the tetrahedral SiO_4_ surface and for each metal ion, 35.7% are presented as outer-sphere adsorption (Tables 1 and 2), as testified by radial distribution functions (RDF) in Figure S15. Outer-sphere Na^+^ and Pb^2+^ ions departure from the tetrahedral SiO_4_ surfaces by approximately 4.9 and 5.1 Å and are coordinated with an average of 4.2 and 5.3 water molecules, respectively. The coordination numbers of water molecules are slightly less than in bulk solutions (e.g., 5.6 for Pb^2+^), suggesting the influence by kaolinite surfaces although gently.

The outer-sphere Na^+^ ions are doubled when ionic concentrations change from 0.16 to 0.32 mol/L while do not expand so obviously with the further increase of ionic concentrations (Table 1). It indicates that the outer-sphere adsorption sites are gradually saturated, and as a result, a larger proportion of Na^+^ ions remain in bulk solutions and inner-sphere mode emerges at concentrated solutions (one and two at 0.80 and 0.96 mol/L, respectively). Inner-sphere Na^+^ ions are not situated at the center of hexagonal cavity but instead interact mainly with one O_b_ atoms (Fig. 3a), and such adsorption configurations are caused by the involvement of water solvent during the adsorption processes rather than the small ionic size of Na^+^ as proposed before^27^, which is further testified by density functional calculations in the supplementary information. The gradual increase of ionic concentrations also increase the number of outer-sphere Pb^2+^ ions until saturated (Table 2); however, even if saturated, inner-sphere mode is absent and this differs from the case of Na^+^ adsorption^27^.

The time-dependent trajectories of both Na^+^ and Pb^2+^ ions are dispersive for the whole ionic concentration range (Figure S16), suggesting that adsorbed metal ions on regular kaolinite are generally highly mobile and interact weakly with the tetrahedral SiO_4_ surface. In order to quantify the stabilities of metal ions, root mean square fluctuations (RMSF) are calculated (Figure S17 and S18) and then partitioned into four groups (Table 3): (1) RMSF ≤ 1.2 Å. Strong inner-sphere adsorption; (2) 1.2 Å < RMSF ≤ 1.7 Å. Remaining inner-sphere and strong outer-sphere metal ions; (3) 1.7 Å < RMSF ≤ 2.7 Å. Remaining outer-sphere metal ions as well as those in bulk solutions but with comparable stability; (4) RMSF > 2.7 Å. The others in bulk solutions. At 0.16 mol/L, almost all outer-sphere Pb^2+^ and Na^+^ ions have RMSF > 1.7 Å and have stabilities close to those in bulk solutions. At 0.96 mol/L, a majority of Na^+^ and Pb^2+^ ions in bulk solutions fall within Group 4 instead of Group 3 as in 0.16 mol/L, implying the reduction of stabilities for metal ions in bulk solutions with increase of ionic concentrations. At the same time, stabilities of some outer-sphere metal ions are improved, especially in the case of Na^+^ ions. Seven strong outer-sphere Na^+^ ions are ascribed with RMSF ≤ 1.7 Å. As to the two inner-sphere Na^+^ ions, one binds tightly on the tetrahedral SiO_4_ surface (RMSF ≤ 1.2 Å) while the other is relatively more mobile (1.2 Å < RMSF ≤ 1.7 Å).

### Defect sites

Configurations of NaCl and PbCl_2_ solutions in equilibrium with defective kaolinite (**Si**_**1**_) are displayed in Fig. 4, S19 and S20. A totally different scenario from the case of regular models (**Si**_**0**_) has been observed. At 0.16 mol/L, all Na^+^ ions can be considered adsorbed, and 12 out of 14 are inner-sphere (Table 1) with distances of ca. 2.20 Å from the tetrahedral SiO_4_ surface as indicated by RDF plots (Figure S21). Accordingly, the distribution of inner- and outer-sphere Na^+^ ions is greatly altered by defect sites and the strong inner-sphere mode is significantly promoted.

With increase of ionic concentrations, the numbers of both inner- and outer-sphere Na^+^ ions show a gradual increase, while their proportions may decline and more Na^+^ ions will remain in bulk solutions. The inner-sphere adsorption sites seem to be filled up since 0.64 mol/L, and a maximum of 25 inner-sphere Na^+^ ions are detected for **Si**_**1**_ instead of merely 2 for **Si**_**0**_. Thus, defect sites substantially enhance the inner-sphere adsorption sites that are known to be strong and stable^9^,^10^,^11^,^12^,^19^,^26^,^27^. In fact, outer-sphere mode is also affected although relatively slightly. At each concentration, the number of outer-sphere Na^+^ ions differs from that of **Si**_**0**_; in addition, the distances to the tetrahedral SiO_4_ surface show a decrease suggesting the reinforced interactions; e.g., at 0.16 mol/L about 4.7 and 4.9 Å for **Si**_**1**_ and **Si**_**0**_, respectively (Fig. 5). A straightforward way to show how the distances are altered by defect sites is given in Fig. 5 that is obtained as the average of the last 2.0 ns trajectories. At 0.16 mol/L, only two Na^+^ ions are less than 5.0 Å **in Si**_**0**_, while all Na^+^ ions fall below 5.0 Å in **Si**_**1**_ and most of them are not more than 2.5 Å (inner-sphere). Three different interactions are observed for **Si**_**1**_ and inner-sphere Na^+^ ions: One resembles that in **Si**_**0**_ (Fig. 3a) and the other two are closely associated with defect sites, respectively interacting through the O_h_ atom of silanol (Fig. 3b) and the bridging O_a_ atom that bonds to the silanol Si atom (Fig. 3c). Thus, it clearly demonstrates the important role of defect sites played during the adsorption processes, consistent with the experimental results^16^,^25^,^35^,^48^,^49^.

At 0.16 mol/L, ten Pb^2+^ ions are outer-sphere adsorbed on the tetrahedral SiO_4_ surface of **Si**_**1**_, twice as that of **Si**_**0**_ (Fig. 4 and Table 2). This is mainly due to the presence of hydrophilic silanol groups in **Si**_**1**_^50^,^51^,^52^, which improve the adsorption of water molecules and further outer-sphere Pb^2+^ ions. As a result of enhanced interactions, outer-sphere Pb^2+^ ions in **Si**_**1**_ are more close to the tetrahedral SiO_4_ surface than in **Si**_**0**_ (4.3 vs. 5.1 Å, see Fig. 5). The outer-sphere adsorption sites have been filled up since 0.48 mol/L, and at most 22 Pb^2+^ ions can be adsorbed in this mode. Figure 5 shows that three Pb^2+^ ions have distances of ca. 2.91 Å from the tetrahedral SiO_4_ surface that belong to inner-sphere mode. Unlike the outer-sphere case, the inner-sphere adsorption quantities seem not to be much affected by increasing ionic concentrations, and four are ascribed to this mode at 0.96 mol/L with distances of ca. 2.99 Å from the tetrahedral SiO_4_ surface (Fig. 5). More importantly, all inner-sphere Pb^2+^ ions are closely associated with defect sites (Fig. 3f), different from the case of Na^+^ adsorption. Accordingly, Pb^2+^ adsorption is fundamentally changed by defect sites, not only enhancing the outer-sphere adsorption quantities but also creating the inner-sphere mode.

Stabilities of metal ions adsorbed on **Si**_**1**_ are addressed by the time-evolution trajectories (Fig. 6 and S22) and RMSF analyses (Table 3). The trajectories of **Si**_**1**_ are obviously more concentrated than those of **Si**_**0**_. Inner-sphere metal ions are restricted at the adsorption sites; meanwhile, outer-sphere Pb^2+^ ions are also stabilized by the defect sites through the tight anchoring of intermediate water molecules. RMSF data show that a considerable amount of metal ions is transferred to Groups 1 and 2; e.g., in addition to inner-sphere ones, another four and seven Pb^2+^ ions have RMSF ≤ 1.7 Å respectively at 0.16 and 0.96 mol/L that should belong to strong outer-sphere adsorption. Accordingly, defect sites improve the stabilities of both inner- and outer-sphere metal ions and generate long-lived adsorbed species. Both of the adsorption quantities and stabilities are significantly promoted by defect sites, which give a reasonable interpretation for the good adsorption behaviors observed experimentally^2^,^28^,^29^.

### Extending defect sites

Figure 7, S23 and S24 display the equilibrium configurations of kaolinite with a larger defect site (**Si**_**2**_) in contact with NaCl and PbCl_2_ solutions. The adsorption behaviors are similar for **Si**_**2**_ and **Si**_**1**_, and both inner-sphere Na^+^ and Pb^2+^ ions occur at the whole concentration ranges (0.16 ∼ 0.96 mol/L). According to the types of bonded O(O_a_/O_b_/O_h_) atoms, three (Fig. 3a,d,e) and one (Fig. 3g) interactions are respectively assigned for inner-sphere Na^+^ and Pb^2+^ ions that resemble those of **Si**_**1**_. At 0.16 mol/L, most Na^+^/Pb^2+^ ions are already inner-sphere/outer-sphere adsorbed in **Si**_**1**_ and hence the adsorption quantities will remain almost unaltered by enlargement of defect sizes to **Si**_**2**_ (Tables 1 and 2). With increase of ionic concentrations, the adsorption quantities of **Si**_**2**_ may show increases as compared to **Si**_**1**_, and inner-sphere Na^+^ and outer-sphere Pb^2+^ ions of **Si**_**2**_**/Si**_**1**_ amount to 26/24 and 25/21 at 0.96 mol/L, respectively. Meanwhile, **Si**_**2**_ is more structurally flexible and this facilitates the interactions with metal ions. The adsorption energies of inner-sphere Na^+^/Pb^2+^ ions with defective sites (*E*_K_) are approximately −323.5 ± 4.3/−331.1 ± 4.8 and −334.8 ± 4.0/−342.8 ± 4.1 kJ/mol for **Si**_**1**_ and **Si**_**2**_, respectively. As a result of reinforced interactions, distances to the tetrahedral SiO_4_ surface are slightly shortened with enlargement of defect sites (Fig. 5). There are also inner-sphere Na^+^ ions that have no relation with defect sites, and their interactions are obviously weaker as reflected by the *E*_K_ values that are equal to −230.9 ± 5.3 kJ/mol for **Si**_**1**_ and −239.2 ± 4.9 kJ/mol for **Si**_**2**_, respectively. As a result, inner-sphere Pb^2+^ ions can be detected only at defect sites that enhance the interactions (Fig. 6).

**Figure 1:**
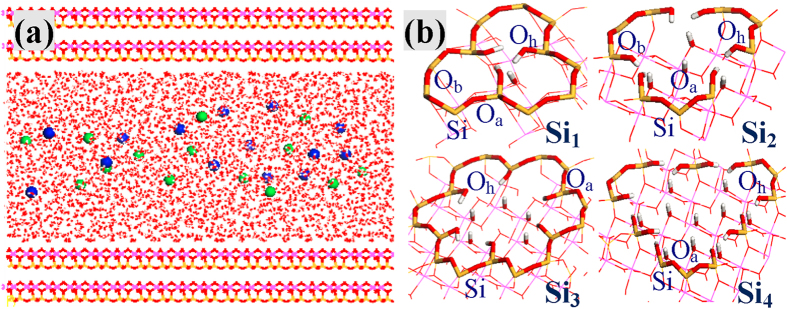
(**a**) Initial configuration of regular kaolinite in 0.16 mol/L NaCl solutions and (**b**) local structures of the various defect sites (**Si**_**1**_, **Si**_**2**_, **Si**_**3**_ and **Si**_**4**_) created at the tetrahedral SiO_4_ surface. The tetrahedral SiO_4_ and octahedral AlO_6_ surfaces of kaolinite that have interfacial interactions with salt solutions are situated at the top and bottom, respectively. O_h_ is from the hydroxyl of silanol, and O_a_ (bridging O) is also bonded to the silanol Si atom while O_b_ (bridging O) has no relation with defect sites. Na^+^ and Cl^−^ ions are represented as blue and green balls, respectively.

**Figure 2:**
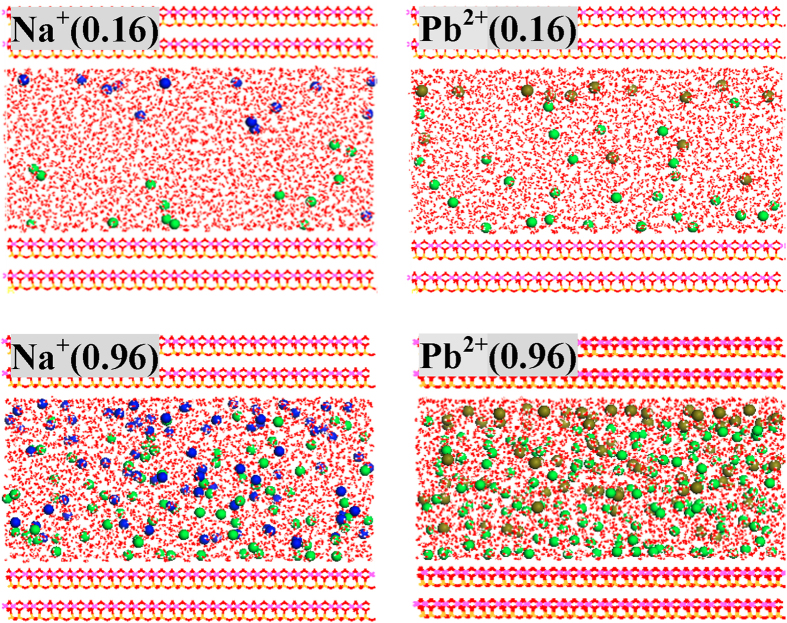
Configurations of regular kaolinite in equilibration with 0.16 and 0.96 mol/L NaCl/PbCl_2_ solutions. Ionic concentrations (mol/L) are indicated in the parentheses of legends. Na^+^, Pb^2+^ and Cl^−^ ions are represented as blue, dark yellow and green balls, respectively.

**Figure 3:**
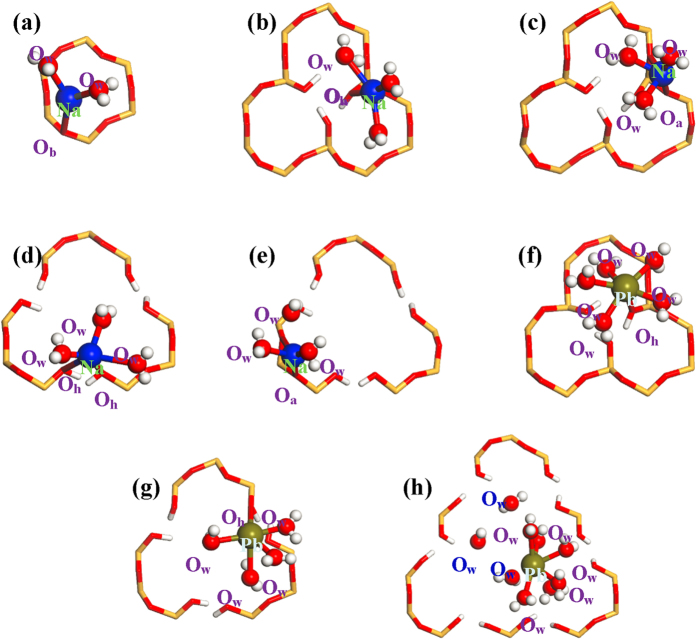
Local structures of inner-sphere Na^+^ and Pb^2+^ ions adsorbed on the tetrahedral SiO_4_ surfaces of regular (Si_0_) and defective kaolinite (Si_1_, Si_2_ and Si_4_). (**a**) for **Si**_**0**_(Na^+^), (**a**–**c**) for **Si**_**1**_(Na^+^), (**a**,**d**,**e**) for **Si**_**2**_(Na^+^) as well as (**f**) for **Si**_**1**_(Pb^2+^), (**g**) for **Si**_**2**_(Pb^2+^) and (**h**) for **Si**_**4**_(Pb^2+^), respectively. Note that **Si**_**4**_(Pb^2+^) is actually for quasi inner-sphere Pb^2+^ ions adsorbed on the water molecules within the silanol nests of **Si**_**4**_.

**Figure 4:**
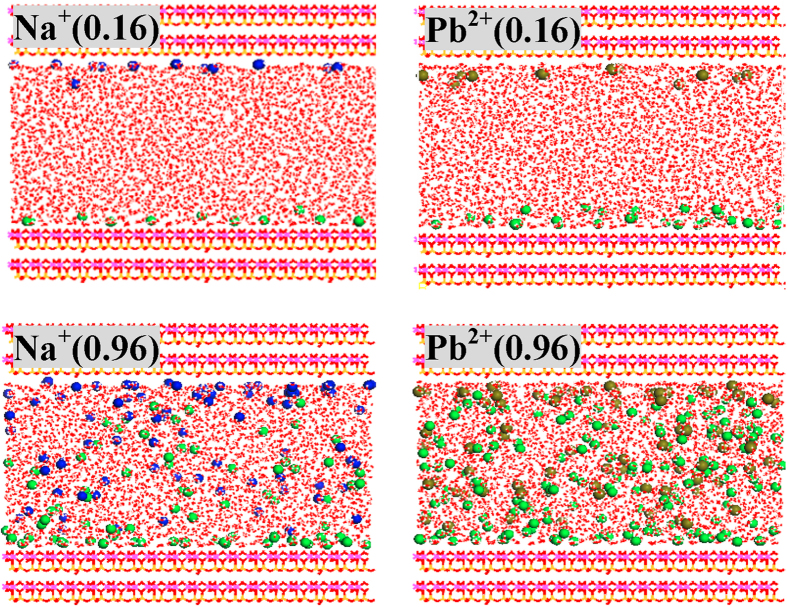
Configurations of defective kaolinite (Si_1_) in equilibration with 0.16 and 0.96 mol/L NaCl/PbCl_2_ solutions. Ionic concentrations (mol/L) are indicated in the parentheses of legends. Na^+^, Pb^2+^ and Cl^−^ ions are represented as blue, dark yellow and green balls, respectively.

**Figure 5:**
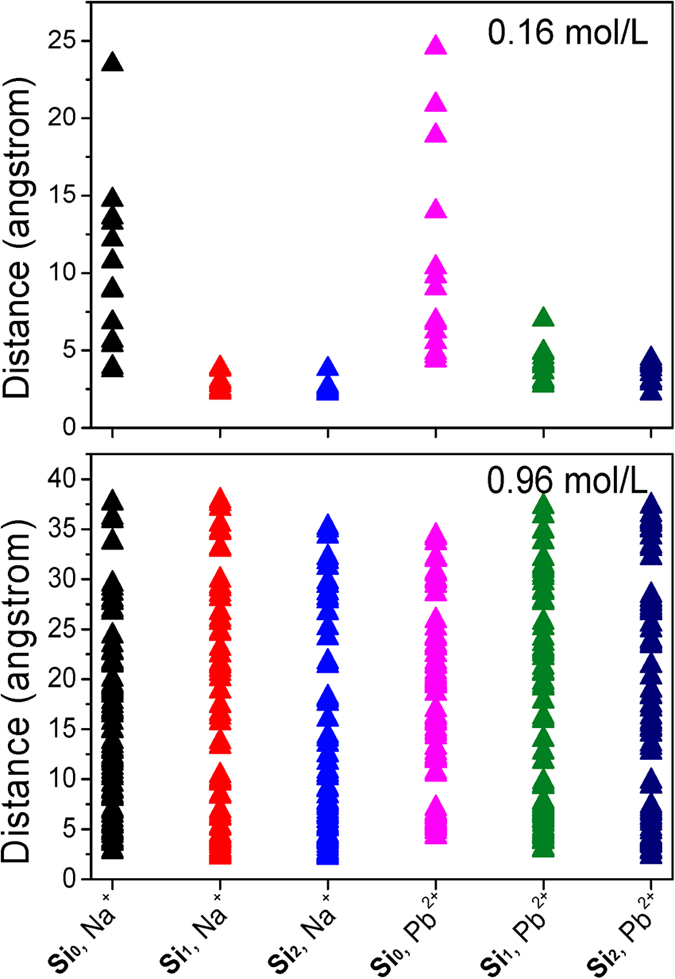
Distances between the tetrahedral SiO_4_ surfaces of kaolinite and Na^+^/Pb^2+^ ions from NaCl/PbCl_2_ solutions.

In order to clarify the effect of defect sizes, two larger defects (**Si**_**3**_ and **Si**_**4**_, see Fig. 1b) are considered, and their equilibrium configurations in 0.16 and 0.96 mol/L NaCl and PbCl_2_ solutions are given in Figures S25, S26. At low ionic concentrations (e.g., 0.16 mol/L), the further enlargement of defect sizes from **Si**_**2**_ to **Si**_**3**_ and **Si**_**4**_ causes slight alterations to the adsorption of inner-sphere Na^+^ and outer-sphere Pb^2+^ ions, which is in agreement with the results of **Si**_**2**_ vs. **Si**_**1**_ discussed earlier; at 0.96 mol/L, however, the numbers of inner-sphere Na^+^ ions of **Si**_**3**_ and **Si**_**4**_ show observable increases and amount to 30 and 35, respectively, while at any given concentration, the quantities of outer-sphere Na^+^ ions remain almost constant for all defective models (Table 1). Figure 3 and S27 indicate that the adsorption structures of inner-sphere Na^+^ ions are similar for all defect sites. With increase of defect sizes, however, inner-sphere Na^+^ ions have an attendance to be closer to the center of hydrophilic silanol nests, probably due to the attraction by water molecules inside these silanol nests. Similarly, at high concentrations, outer-sphere Pb^2+^ ions are promoted by the further enlargement of defect sites, which, at 0.96 mol/L, are counted to be 25, 29 and 32 for **Si**_**2**_, **Si**_**3**_ and **Si**_**4**_, respectively (Table 2). In the case of **Si**_**4**_, the inner-sphere Pb^2+^ species vanishes at both 0.16 and 0.96 mol/L, and a quasi inner-sphere mode emerges instead, where Pb^2+^ ions form direct bonds with water molecules that have entered into the silanol nests (Fig. 3h)^31^. Although not associated directly with kaolinite, the vertical distances of quasi inner-sphere Pb^2+^ ions to the tetrahedral SiO_4_ surface fall within the scope of inner-sphere mode.

**Figure 6:**
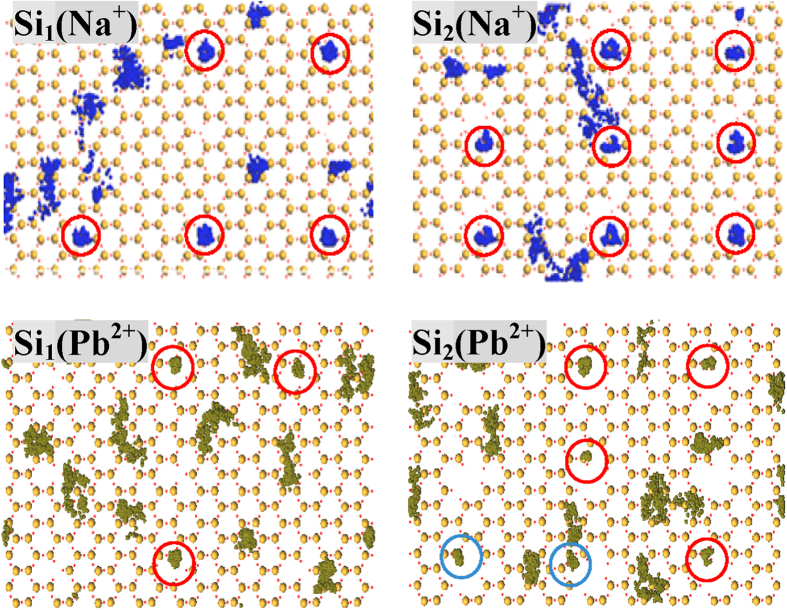
Trajectory maps of tetrahedral SiO_4_ surfaces of defective kaolinite (Si_1_ and Si_2_) in contact with 0.16 mol/L NaCl and PbCl_2_ solutions. Only the inner- and outer-sphere metal ions are made visible, and Na^+^ and Pb^2+^ ions are represented as blue and dark yellow balls, respectively. Inner- and outer-sphere metal ions that fall around the defective sites are highlighted by red and light blue circles, respectively.

**Figure 7:**
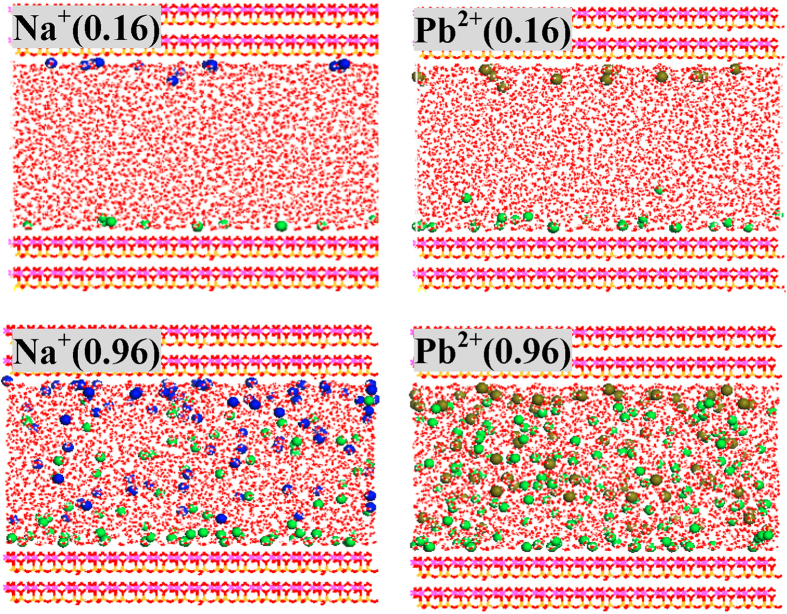
Configurations of defective kaolinite (Si_2_) in equilibration with 0.16 and 0.96 mol/L NaCl/PbCl_2_ solutions. Ionic concentrations (mol/L) are indicated in the parentheses of legends. Na^+^, Pb^2+^ and Cl^−^ ions are represented as blue, dark yellow and green balls, respectively.

Stabilities of adsorbed metal ions improve gradually with the enlargement of defect sites from **Si**_**1**_ to **Si**_**2**_ and then to **Si**_**3**_ and **Si**_**4**_, in line with the adsorption results on zeolites and graphene^35^,^53^. As indicated by the time-evolution trajectories (Fig. 6, S22, S28 and S29), adsorbed metal ions become more concentrated on the tetrahedral SiO_4_ surface due to the increase of defect sizes; meanwhile, more metal ions are directly associated with defect sites; e.g., the numbers of such inner-sphere Na^+^ ions at 0.16/0.96 mol/L are 5/9, 8/13, 8/15, 9/19 for **Si**_**1**_, **Si**_**2**_, **Si**_**3**_ and **Si**_**4**_, respectively. A more elaborate analysis of stabilities is carried out based on the RMSF data (Table 3). Inner-sphere Na^+^ ions at 0.16/0.96 mol/L with RMSF ≤ 1.2 Å are counted to be 0/1, 6/18, 9/21, 10/26 and 10/30 for **Si**_**0**_, **Si**_**1**_, **Si**_**2**_, **Si**_**3**_ and **Si**_**4**_, respectively. It clearly shows that the increase of defect sizes significantly stabilizes the inner-sphere Na^+^ ions, especially at higher ionic concentrations. Meanwhile, other Na^+^ ions are also stabilized, and so fewer will stay with RMSF > 2.7 Å. With the gradual enlargement of defect sizes, similar changing trends are observed for the stabilities of Pb^2+^ ions. At 0.16/0.96 mol/L, Pb^2+^ ions with strong outer-sphere adsorption (1.2 Å < RMSF ≤ 1.7 Å) are counted to be 1/3, 4/7, 9/16, 10/20 and 10/24 for **Si**_**0**_, **Si**_**1**_, **Si**_**2**_, **Si**_**3**_ and **Si**_**4**_, respectively. The quasi inner-sphere Pb^2+^ ions that occur in the case of **Si**_**4**_ are greatly stabilized by associated water molecules within the hydrophilic silanol nests and show comparable stabilities as inner-sphere species (RMSF < 1.2 Å, see Table 3).

### Adsorption capacities and mechanisms

The adsorption capacities (*Γ*) of Na^+^ and Pb^2+^ ions on the tetrahedral SiO_4_ surfaces of kaolinite are calculated for the various defect sites (**Si**_**0**_, **Si**_**1**_, **Si**_**2**_, **Si**_**3**_ and **Si**_**4**_) and a wide range of ionic concentrations (0.16 ∼ 0.96 mol/L), see Tables 1 and 2 and Figures S30, S31. For a given model (e.g., **Si**_**1**_), the adsorption quantities of both inner- and outer-sphere metal ions generally increase with ionic concentrations until saturated. Outer- and inner-sphere Na^+^ ions are respectively the principal adsorbed species for regular and defective models, and the amounts of the former remain close for all models (regular and defective) while those of the latter increase with enlargement of defect sizes. Outer-sphere Pb^2+^ ions that predominate for all models show an increase with enlargement of defect sizes. At 0.96 mol/L, the adsorption capacities of inner-sphere Na^+^/outer-sphere Pb^2+^ ions amount to 0.09/0.71, 1.07/0.93, 1.16/1.11, 1.33/1.29 and 1.56/1.42 μmol/m^2^ for **Si**_**0**_, **Si**_**1**_, **Si**_**2**_, **Si**_**3**_ and **Si**_**4**_, accounting for 12.68%/100.00%, 61.49%/83.78%, 63.39%/100.00% and 68.72%/94.04% of the total adsorption capacities, respectively.

Defect contents may also affect the adsorption processes^49^,^54^, and so 18 and 27 defect sites of **Si**_**1**_ type are considered. Figure S32 shows their configurations in equilibrium with 0.96mol/L NaCl and PbCl_2_ solutions. The adsorption behaviors of both Na^+^ and Pb^2+^ ions remain the same for all defect contents, and the adsorption sites seem to have a finite rate of increase when the number of defect sites (n) is doubled (n = 18) and then tripled (n = 27), see Tables 1 and 2 and Figure S29. Inner-sphere Na^+^ ions that predominate for all defective models are the most affected and the adsorption capacities amount to 1.07, 1.38 and 1.78 μmol/m^2^ respectively for n = 9, 18 and 27 (0.96 mol/L). The adsorption capacities of outer-sphere Na^+^ and Pb^2+^ ions increase relatively slowly with increase of defect contents, while inner-sphere Pb^2+^ ions that are minor remain constant for all defect contents. Stabilities of adsorbed metal ions improve with increase of defect contents (Table S2): RMSFs of more Na^+^ ions and Pb^2+^ ions fall respectively below 1.2 Å (strong inner-sphere) and 1.7 Å (strong outer-sphere), and fewer metal ions remain with RMSF > 2.7 Å.

**Figure 8:**
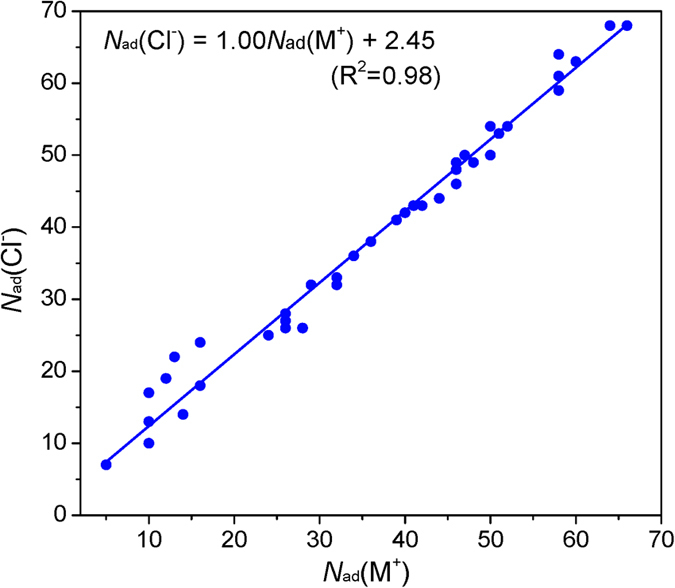
Correlation of the adsorbed quantities between metal cations (M^+^) and anions (Cl^−^). Both inner- and outer-sphere adsorbed ions are counted. Pb^2+^ is two molar equivalents of M^+^; i.e., *N*_ad_(Pb^2+^) = 2*N_ad_(M^+^).

As discussed above, defect sites promote the adsorption of metal ions, with respect to both adsorption quantities and stabilities; in addition, the adsorption capacities are closely associated with ionic concentrations, defect sizes and contents. Adsorption of metal ions and anions may proceed in a collaborative mechanism, see Fig. 8. The octahedral AlO_6_ surface with slightly positive charges attracts Cl^−^ ions strongly and so an average of 2.45 more Cl^−^ ions will be adsorbed than M^+^ ions. Defect sites are developed on the tetrahedral SiO_4_ surface and have no observable impact on Cl^−^ ions, while the adsorption quantities of Cl^−^ ions show a linear increase along with those of metal ions (M^+^). Accordingly, metal ions and anions can promote each other during the adsorption processes. The slope of the fitted line in Fig. 8 is approximately 1.00 and this demonstrates that on average, the adsorption of one more M^+^ ion results in the increase of one adsorbed Cl^−^ ion; vice versa.

## Computational Details

### Models

Kaolinite has alternative tetrahedral SiO_4_ sheet and octahedral AlO_6_ sheet that are bridged by O atoms. The crystal structure of kaolinite was taken from ref. 55 with cell parameters being a = 5.19 Å, b = 8.96 Å, c = 7.36 Å, α = 90.77^o^, β = 104.17^o^ and γ = 90.40^o^. Models were prepared in line with the report of Vasconcelos *et al.*^27^. Firstly, the orthogonal transformation was conducted causing the (001) and (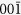) planes of kaolinite respectively to correspond to the octahedral AlO_6_ and tetrahedral SiO_4_ surfaces; secondly, models with 324 unit cells (9 × 9 × 4 along *x*, *y* and *z* directions) were constructed, and the inward octahedral AlO_6_ and tetrahedral SiO_4_ surfaces were separated by a vacuum layer with thickness of 40 Å; thirdly, the aqueous-kaolinite interfaces were achieved by filling 5097 water molecules into the vacuum layer. Because of the choice of a thicker vacuum layer, more water molecules are included in this work than previously^27^, resulting in a total of 26307 atoms; finally, NaCl and PbCl_2_ solutions with a wide range of concentrations (0.16, 0.32, 0.48, 0.64, 0.80 and 0.96 mol/L) were respectively prepared by replacing certain numbers of water molecules with metal cations and anions. These models are referred to “regular” (**Si**_**0**_), and Fig. 1a illustrates the initial configuration of regular kaolinite (**Si**_**0**_) in contact with 0.16 mol/L NaCl solutions.

A series of defect sites^5^,^6^,^31^,^32^,^33^,^34^,^35^,^36^,^56^,^57^ were then constructed on the tetrahedral SiO_4_ surface of regular model (Fig. 1b), by removing one lattice Si atom (**Si**_**1**_) as well as first-shell Si atoms (**Si**_**2**_) and further second-shell Si atoms (**Si**_**4**_), respectively. In addition, **Si**_**3**_ was considered where the six Si atoms of one hexagonal cavity were all leached. The hexagonal cavity was assumed to be the main loci for situating metal ions^27^. Density functional calculated results in the supplementary information showed that defect sites are facile to form in clay minerals. The defect sizes follow as **Si**_**1**_ < **Si**_**2**_ < **Si**_**3**_ < **Si**_**4**_, with removal of 1, 4, 6 and 10 lattice Si atoms, respectively. Note that all these defect sites are far smaller than the trenches reported by Croteau^31^. The dangling O atoms of defect sites were saturated by H atoms to ensure electroneutrality. Unless otherwise noted, the number of defect sites is 9 for defective models. To investigate the effect of defect contents on the adsorption processes, 18 and 27 defect sites of **Si**_**1**_ type were also taken in account. All concentrations of NaCl and PbCl_2_ solutions indicated above were simulated for **Si**_**0**_, **Si**_**1**_ and **Si**_**2**_, as well as 0.16 and 0.96 mol/L for **Si**_**3**_, **Si**_**4**_ and 0.96 mol/L for **Si**_**1**_ with 18 and 27 defect sites, resulting in a total of 48 models.

### Methods

MD simulations with periodic boundary conditions (PBC) were performed by using Gromacs suite of programs (Version 4.6.5)^58^. A combination of CLAYFF force field (parameters given in Table S1)^59^ and simple-point-charge (SPC) water model^60^ was used. Recently, the CLAYFF force field has achieved unprecedented success in modeling clay minerals, and the conjunction with SPC water model has been demonstrated to be accurate to simulate hydrated minerals and aqueous-mineral interfaces^14^,^23^,^27^,^61^,^62^,^63^,^64^,^65^. Non-bonded (electrostatic and vdW) terms that were developed especially for layered minerals were parametrized in the CLAYFF force field. The Particle-Mesh-Ewald (PME) method was employed to treat long-range electrostatic interactions, and the cutoff radii for Ewald electrostatic summation and long-range vdW interactions were set to 12.0 Å. The leapfrog algorithm^66^ was used, and 5.0 ns MD simulations were run for each model, at constant temperature and pressure (300 K, 1.0 bar) that were respectively controlled by the V-rescale thermostat^67^ and Parrinello-Rahman barostat^68^. The time step was 2.0 fs, and the atomic coordinates were updated every 1.0 ps. According to the root-mean-square-deviation (RMSD) results (Figures S3, S12), all models have arrived to the equilibrium since 2.0 ns, and hence the last 3.0 ns trajectories were used for analyses. The adsorption energies of metal ions on kaolinite surfaces were calculated on the average structures of 2.0 ∼ 5.0 ns MD trajectories, considering the regions within 10.0 Å around the metal ions.

## Additional Information

**How to cite this article**: Li, X. *et al.* Promoting the Adsorption of Metal Ions on Kaolinite by Defect Sites: A Molecular Dynamics Study. *Sci. Rep.* **5**, 14377; doi: 10.1038/srep14377 (2015).

## Supplementary Material

Supplementary Information

## Figures and Tables

**Table 1:** Percentage (*X*_ad_), number (*N*_ad_) and adsorption capacity (*Γ*, μmol/m^2^) of Na^+^ ions adsorbed on the kaolinite surfaces, where Na^+^ ions are from NaCl solutions with a wide range of concentrations^a^.

	*c* (mol/L)	Inner-sphere adsorption			Outer-sphere adsorption		
		*X*_ad_	*N*_ad_	*Γ*	*X*_ad_	*N*_ad_	*Γ*
Si_0_	0.16	0.0% ± 0.0%	0 ± 0	0.00 ± 0.00	35.7% ± 7.1%	5 ± 1	0.22 ± 0.04
	0.32	0.0% ± 0.0%	0 ± 0	0.00 ± 0.00	35.7% ± 3.6%	10 ± 1	0.45 ± 0.04
	0.48	0.0% ± 0.0%	0 ± 0	0.00 ± 0.00	23.8% ± 4.8%	10 ± 2	0.45 ± 0.09
	0.64	0.0% ± 0.0%	0 ± 0	0.00 ± 0.00	21.4% ± 3.6%	12 ± 2	0.53 ± 0.09
	0.80	1.4% ± 0.0%	1 ± 0	0.04 ± 0.00	17.1% ± 4.3%	12 ± 3	0.53 ± 0.13
	0.96	2.4% ± 1.2%	2 ± 1	0.09 ± 0.04	16.7% ± 3.6%	14 ± 3	0.62 ± 0.13
Si_1_(9)	0.16	85.7% ± 0.0%	12 ± 0	0.53 ± 0.00	14.3% ± 0.0%	2 ± 0	0.09 ± 0.00
	0.32	60.7% ± 3.6%	17 ± 1	0.76 ± 0.04	32.1% ± 7.1%	9 ± 2	0.40 ± 0.09
	0.48	45.2% ± 2.4%	19 ± 1	0.85 ± 0.04	31.0% ± 2.4%	13 ± 1	0.58 ± 0.04
	0.64	41.1% ± 3.6%	23 ± 2	1.02 ± 0.09	19.6% ± 1.8%	11 ± 1	0.49 ± 0.04
	0.80	35.7% ± 4.3%	25 ± 3	1.11 ± 0.13	15.7% ± 2.9%	11 ± 2	0.49 ± 0.09
	0.96	28.6% ± 3.6%	24 ± 3	1.07 ± 0.13	17.9% ± 3.6%	15 ± 3	0.67 ± 0.13
Si_2_(9)	0.16	92.9% ± 7.1%	13 ± 1	0.58 ± 0.04	7.1% ± 7.1%	1 ± 1	0.04 ± 0.04
	0.32	67.9% ± 3.6%	19 ± 1	0.85 ± 0.04	25.0% ± 7.1%	7 ± 2	0.31 ± 0.09
	0.48	47.6% ± 4.8%	20 ± 2	0.89 ± 0.09	21.4% ± 4.8%	9 ± 2	0.40 ± 0.09
	0.64	44.6% ± 1.8%	25 ± 1	1.11 ± 0.04	19.6% ± 1.8%	11 ± 1	0.49 ± 0.04
	0.80	38.6% ± 2.9%	27 ± 2	1.20 ± 0.09	18.6% ± 4.3%	13 ± 3	0.58 ± 0.13
	0.96	31.0% ± 3.6%	26 ± 3	1.16 ± 0.13	17.9% ± 3.6%	15 ± 3	0.67 ± 0.13
Si_3_(9)	0.16	85.7% ± 7.1%	12 ± 1	0.53 ± 0.04	14.3% ± 7.1%	2 ± 1	0.09 ± 0.04
	0.96	35.7% ± 2.4%	30 ± 2	1.33 ± 0.09	19.0% ± 2.4%	16 ± 2	0.71 ± 0.09
Si_4_(9)	0.16	92.9% ± 7.1%	13 ± 1	0.58 ± 0.04	7.1% ± 7.1%	1 ± 1	0.04 ± 0.04
	0.96	41.7% ± 3.6%	35 ± 3	1.56 ± 0.13	19.0% ± 2.4%	16 ± 2	0.71 ± 0.09
Si_1_(18)	0.96	36.9% ± 1.2%	31 ± 1	1.38 ± 0.04	19.0% ± 1.2%	16 ± 1	0.71 ± 0.04
Si_1_(27)	0.96	47.1% ± 3.6%	40 ± 3	1.78 ± 0.13	21.4% ± 2.4%	18 ± 2	0.80 ± 0.09

^a^The number of defect sites are given in parentheses, and the default is 9.

**Table 2:** Percentage (*X*_ad_), number (*N*_ad_) and adsorption capacity (*Γ*, μmol/m^2^) of Pb^2+^ ions adsorbed on the kaolinite surfaces, where Pb^2+^ ions are from PbCl_2_ solutions with a wide range of concentrations^a^.

	*c* (mol/L)	Inner-sphere adsorption			Outer-sphere adsorption		
		*X*_ad_	*N*_ad_	*Γ*	*X*_ad_	*N*_ad_	*Γ*
Si_0_	0.16	0.0% ± 0.0%	0 ± 0	0.00 ± 0.00	35.7% ± 7.1%	5 ± 1	0.22 ± 0.04
	0.32	0.0% ± 0.0%	0 ± 0	0.00 ± 0.00	25.0% ± 3.6%	7 ± 1	0.31 ± 0.04
	0.48	0.0% ± 0.0%	0 ± 0	0.00 ± 0.00	19.0% ± 2.4%	8 ± 1	0.36 ± 0.04
	0.64	0.0% ± 0.0%	0 ± 0	0.00 ± 0.00	23.2% ± 1.8%	13 ± 1	0.58 ± 0.04
	0.80	0.0% ± 0.0%	0 ± 0	0.00 ± 0.00	22.9% ± 2.9%	16 ± 2	0.71 ± 0.09
	0.96	0.0% ± 0.0%	0 ± 0	0.00 ± 0.00	19.0% ± 2.4%	16 ± 2	0.71 ± 0.09
Si_1_(9)	0.16	21.4% ± 0.0%	3 ± 0	0.13 ± 0.00	71.4% ± 7.1%	10 ± 1	0.44 ± 0.04
	0.32	10.7% ± 0.0%	3 ± 0	0.13 ± 0.00	64.3% ± 7.1%	18 ± 2	0.80 ± 0.09
	0.48	7.1% ± 0.0%	3 ± 0	0.13 ± 0.00	47.6% ± 4.8%	20 ± 2	0.89 ± 0.09
	0.64	5.4% ± 0.0%	3 ± 0	0.13 ± 0.00	35.7% ± 1.8%	20 ± 1	0.89 ± 0.04
	0.80	4.3% ± 0.0%	3 ± 0	0.13 ± 0.00	31.4% ± 4.3%	22 ± 3	0.98 ± 0.13
	0.96	4.8% ± 1.2%	4 ± 1	0.18 ± 0.04	25.0% ± 2.4%	21 ± 2	0.93 ± 0.09
Si_2_(9)	0.16	28.6% ± 0.0%	4 ± 0	0.18 ± 0.00	71.4% ± 7.1%	10 ± 1	0.44 ± 0.04
	0.32	14.3% ± 3.6%	4 ± 1	0.18 ± 0.04	64.3% ± 7.1%	18 ± 2	0.80 ± 0.09
	0.48	9.5% ± 2.4%	4 ± 1	0.18 ± 0.04	47.6% ± 2.4%	20 ± 1	0.89 ± 0.04
	0.64	9.0% ± 0.0%	5 ± 0	0.22 ± 0.00	37.5% ± 3.6%	21 ± 2	0.93 ± 0.09
	0.80	7.1% ± 0.0%	5 ± 0	0.22 ± 0.00	35.7% ± 4.3%	25 ± 3	1.11 ± 0.13
	0.96	4.8% ± 1.2%	4 ± 1	0.18 ± 0.04	29.8% ± 2.4%	25 ± 2	1.11 ± 0.06
Si_3_(9)	0.16	0.0% ± 0.0%	0 ± 0	0.00 ± 0.00	85.7% ± 7.1%	12 ± 1	0.53 ± 0.04
	0.96	0.0% ± 0.0%	0 ± 0	0.00 ± 0.00	34.5% ± 3.6%	29 ± 3	1.29 ± 0.13
Si_4_(9)^b^	0.16	14.3% ± 0.0%	2 ± 0	0.09 ± 0.00	78.6% ± 7.1%	11 ± 1	0.49 ± 0.04
	0.96	2.4% ± 0.0%	2 ± 0	0.09 ± 0.00	38.0% ± 2.4%	32 ± 2	1.42 ± 0.09
Si_1_(18)	0.96	4.8% ± 1.2%	4 ± 1	0.18 ± 0.04	29.8% ± 2.4%	25 ± 2	1.11 ± 0.09
Si_1_(27)	0.96	4.8% ± 1.2%	4 ± 1	0.18 ± 0.04	34.5% ± 2.4%	29 ± 2	1.29 ± 0.09

^a^Numbers of defect sites are given in parentheses, and the default is 9.

^b^Two quasi inner-sphere adsorbed Pb^2+^ ions have been taken into account.

**Table 3:** Numbers of Na^+^ and Pb^2+^ ions falling with the specified RMSF ranges for NaCl and PbCl_2_ solutions in equilibrium with the tetrahedral SiO_4_ surfaces of kaolinite.

*c* (mol/L)	RMSF (Å)	Na^+^					Pb^2+^				
		Si_0_	Si_1_	Si_2_	Si_3_	Si_4_	Si_0_	Si_1_	Si_2_	Si_3_	Si_4_
0.16	≤1.2	0	6	9	10	10	0	1	4	0	2
	1.2 ∼ 1.7	0	6	4	3	4	1	6	9	10	10
	1.7 ∼ 2.7	12	2	1	1	0	11	7	1	4	2
	>2.7	2	0	0	0	0	2	0	0	0	0
0.96	≤1.2	1	18	21	26	30	0	3	4	0	2
	1.2 ∼ 1.7	8	16	18	18	19	3	8	16	20	24
	1.7 ∼ 2.7	28	22	24	21	17	36	43	41	44	40
	>2.7	47	28	21	19	18	45	30	23	20	18
