# Supplementary material for: Promoting the Adsorption of Metal Ions on Kaolinite by Defect Sites: A Molecular Dynamics Study
Source: Sci Rep. 2015 Sep 25;5:14377. doi: 10.1038/srep14377 (PMC4585903; doi:10.1038/srep14377)
Supplement: Supplementary Information [file srep14377-s1.pdf]

Supplementary Information

Promoting the Adsorption of Metal Ions on Kaolinite by Defect Sites: A Molecular Dynamics Study

Xiong Li, Hang Li*, Gang Yang*

Chongqing Key Laboratory of Soil Multi-scale Interfacial Process, College of Resources and Environments, Southwest University, Chongqing 400715, China

Contents:

S1. Details of density functional calculations. ································ P. 6

S.1.1. Defect sites. ·· P. 6

S.1.2. Alteration of Na^+ adsorption by water solvent. ································ P. 7

Figure S1. Cluster models for (a) regular kaolinite (b) kaolinite with Si1 defect site. ·· P. 10

Figure S2. Cluster models for (a) Na^+ adsorption on the tetrahedral SiO_4 surface of regular kaolinite and (b) addition of seven water molecules. ························ P. 11

Table S1. Parameters for the CLAYFF potential. ···································· P. 12

Table S2. Numbers of Na^+ and Pb^{2+} ions falling with the specified RMSF ranges for 0.96 mol/L NaCl and $PbCl_2$ solutions in contact with kaolinite containing different numbers of $\mathbf{S_1}$ defect sites. ·· P.13

Figure S3. RMSD plots for MD simulations of regular kaolinite in contact with NaCl solutions of different ionic concentrations (Ionic concentrations are indicated in the legends). ·· P. 14

Figure S4. RMSD plots for MD simulations of defective kaolinite ($\mathbf{Si_1}$) in contact with NaCl solutions of different ionic concentrations (Ionic concentrations are indicated in the legends). ·· P. 17

Figure S5. RMSD plots for MD simulations of defective kaolinite ($\mathbf{Si_2}$) in contact with NaCl solutions of different ionic concentrations (Ionic concentrations are indicated in the legends). ·· P. 20

Figure S6. RMSD plots for MD simulations of regular kaolinite ($\mathbf{Si_0}$) in contact with $PbCl_2$ solutions of different ionic concentrations (Ionic concentrations are indicated in

the legends). ········· P. 23

Figure S7. RMSD plots for MD simulations of defective kaolinite (**Si_1**) in contact with $PbCl_2$ solutions of different ionic concentrations (Ionic concentrations are indicated in the legends). ········· P. 26

Figure S8. RMSD plots for MD simulations of defective kaolinite (**Si_2**) in contact with $PbCl_2$ solutions of different ionic concentrations (Ionic concentrations are indicated in the legends). ········· P. 29

Figure S9. RMSD plots for MD simulations of defective kaolinite (**Si_1**) in contact with 0.96 mol/L NaCl solutions (Numbers of defect sites are indicated in the legends). ········· P. 32

Figure S10. RMSD plots for MD simulations of defective kaolinite (**Si_1**) in contact with 0.96 mol/L $PbCl_2$ solutions (Numbers of defect sites are indicated in the legends). ········· P. 33

Figure S11. RMSD plots for MD simulations of defective kaolinite (**Si_3** and **Si_4**) in contact with 0.16 and 0.96 mol/L NaCl solutions. ········· P. 34

Figure S12. RMSD plots for MD simulations of defective kaolinite (**Si_3** and **Si_4**) in contact with 0.16 and 0.96 mol/L $PbCl_2$ solutions. ········· P. 36

Figure S13. Configurations of regular kaolinite in equilibrium with (a) 0.32 mol/L, (b) 0.48 mol/L, (c) 0.64 mol/L and (d) 0.80 mol/L NaCl solutions. ········· P. 38

Figure S14. Configurations of regular kaolinite (**Si_0**) in equilibrium with (a) 0.32 mol/L, (b) 0.48 mol/L, (c) 0.64 mol/L and (d) 0.80 mol/L $PbCl_2$ solutions. ········· P. 39

Figure S15. Radial distribution functions (g(r)) and coordination numbers (CN) for Na^+/Pb^{2+} adsorption from 0.16 mol/L NaCl and $PbCl_2$ solutions onto the tetrahedral SiO_4 surfaces of regular kaolinite. ········· P. 40

Figure S16. Trajectory maps of the tetrahedral SiO_4 surfaces of regular kaolinite in contact with 0.16 and 0.96 mol/L NaCl/$PbCl_2$ solutions. ………………………… P. 41

Figure S17. RMSF plots for Na^+/Pb^{2+} for kaolinite surfaces in contact with 0.16 mol/L NaCl/$PbCl_2$ solutions over the 5.0 ns MD simulations. …………………… P. 42

Figure S18. RMSF plots for Na^+/Pb^{2+} for kaolinite surfaces in contact with 0.96 mol/L NaCl/$PbCl_2$ solutions over the 5.0 ns MD simulations. …………………… P. 43

Figure S19. Configurations of defective kaolinite (**Si_1**) in equilibrium with NaCl solutions of different concentrations. ……………………………………………… P. 44

Figure S20. Radial distribution functions (*g*(r)) and coordination numbers (CN) for Na^+ adsorption from 0.16 mol/L NaCl solutions onto the tetrahedral SiO_4 surfaces of defective kaolinite (**Si_1** and **Si_2**). ………………………………………………… P. 45

Figure S21. Configurations of defective kaolinite (**Si_1**) in equilibration with $PbCl_2$ solutions of different concentrations. ……………………………………………… P. 46

Figure S22. Trajectory maps of the tetrahedral SiO_4 surfaces of defective kaolinite (**Si_1** and **Si_2**) in contact with 0.96 mol/L NaCl and $PbCl_2$ solutions. ……………… P. 47

Figure S23. Configurations of defective kaolinite (**Si_2**) in equilibration with NaCl solutions of different concentrations. ……………………………………………… P. 48

Figure S24. Configurations of defective kaolinite (**Si_2**) in equilibration with $PbCl_2$ solutions of different concentrations. ……………………………………………… P. 49

Figure S25. Configurations of kaolinite with **Si_3** and **Si_4** defect sites in equilibrium with 0.16 mol/L NaCl and $PbCl_2$ solutions. ……………………………………… P. 50

Figure S26. Configurations of kaolinite with **Si_3** and **Si_4** defect sites in equilibration with 0.96 mol/L NaCl and $PbCl_2$ solutions. ……………………………………… P. 51

Figure S27. Local structures of inner-sphere Na^+ ions adsorbed on the tetrahedral SiO_4 surfaces of defective kaolinite (**Si_3** and **Si_4**). ···································· P. 52

Figure S28. Trajectory maps of the tetrahedral SiO_4 surfaces of defective kaolinite (**Si_3** and **Si_4**) in contact with 0.16 mol/L NaCl and $PbCl_2$ solutions. ··············· P. 53

Figure S29. Trajectory maps of the tetrahedral SiO_4 surfaces of defective kaolinite (**Si_3** and **Si_4**) in contact with 0.96 mol/L NaCl and $PbCl_2$ solutions. ··············· P. 54

Figure S30. Adsorption capacities (Γ) of Na^+/Pb^{2+} ions from NaCl/$PbCl_2$ solutions with a wide range of concentrations onto the tetrahedral SiO_4 surfaces of kaolinite. ·· P. 55

Figure S31. Effects of (a) the size of defect sites and (b) the content of **Si_1** defect site on adsorption capacities (Γ) of Na^+/Pb^{2+} ions from 0.96 mol/L NaCl/$PbCl_2$ solutions on the tetrahedral SiO_4 surface of kaolinite. ··· P. 56

Figure S32. Configurations of kaolinite with different contents of **Si_1** defect site in equilibration with 0.96 mol/L NaCl and $PbCl_2$ solutions (The numbers of **Si_1** defect site are indicated in the parentheses of legends). ···································· P. 57

S1. Details of density functional calculations

As shown in Figure S1(a), the cluster models of kaolinite presently used contain 12 Si and 12 Al atoms. The Si/Al ratio equals 1.0 and agrees with the chemical formulas of kaolinite; i.e., $Al_2Si_2O_5(OH)_4$. In order to remain electroneutral, the boundary O atoms of cluster models were saturated by H atoms. According to the previous studies [1-5], the cluster models of kaolinite were divided into two regions and simulated at different theoretical levels. The hexagonal ring of tetrahedral SiO_4 surface may interact directly with metal cations [3, 6], and so the related O and Si atoms were selected as the high-level region (in ball and stick, see Figure S1(a)). While the rest atoms were treated as the low-level region (in stick).

Density functional calculations were performed with Gaussian09 software packages [7], and the high- and low-level regions were described by the B3LYP/6-31+G(d,p) and B3LYP/3-21G methods, respectively [1-5].

S.1.1. Defect sites

Defect site of $\mathbf{Si_1}$ type is constructed in the tetrahedral SiO_4 surface of kaolinite, see Figure S1(b). The formation energy of $\mathbf{Si_1}$ defect site (E_F) can be written as,

$$E_F = E(\mathbf{Si_1}) + E(Si(OH)_4) - E(kaolinte) - 4*E(H_2O)$$

where E(kaolinte) and $E(\mathbf{Si_1})$ are the energies of regular kaolinite and kaolinite with $\mathbf{Si_1}$ defect site, respectively.

The formation energy of $\mathbf{Si_1}$ defect site (E_F) is calculated to be 13.6 kJ/mol, which is so small that can be facilely overcome at room temperatures. In addition, clay minerals are known to be produced through magmatism that occurs at very high temperatures, and high temperatures will greatly facilitate the formation of defect sites [8-11]. Accordingly, defect sites should be ubiquitous in clay minerals. It is consistent with the fact that a large fraction of carbon resources is found to reside at the defects

of minerals in the Earth's mantle [12].

S.1.2. Alteration of Na^+ adsorption by water solvent

In absence of water solvent, the Na^+ ions are found to be situated symmetrically above the hexagonal cavity with the formation of three direct Na-O_b bonds, see Figure S2(a). Addition of water molecules alters the interaction configuration, and the Na^+ ions deviate significantly from the center of the hexagonal cavity. Figure S2(b) shows that in presence of seven water molecules, the Na^+ ions are associated directly with only one O_b atom. Hence, it is water solvent instead of small size of Na^+ ions as proposed before [6] that results in the alteration of the Na^+ adsorption configuration shown in Figure 3(a). Meanwhile, the CLAYFF force field that has been sufficiently testified by previous MD simulations [13-20] is validated again by density functional calculations. In addition to the consistent adsorption configurations, MD simulations and density functional calculations have close Na-O_b distances, at 2.82 and 2.91 Å, respectively.

References:

1. Tian, R., Yang, G., Zhu, C., Liu, X. M. & Li, H. Specific anion effects for aggregation of colloidal minerals: a joint experimental and theoretical study. *J. Phys. Chem. C* **119**, 4856-4864 (2015).

2. Gao, X. D. et al. Formation of sandwich structure through ion adsorption at the mineral and humic interfaces: A combined experimental computational study. *J. Mol. Struct.* **1093**, 96-100. (2015)

3. Tian, R., Yang, G., Tang, Y.; Liu, X. M., Li, R., Zhu, H. L. & Li, H. Origin of Hofmeister Effects for Complex Systems. *PLOS One* **10**, e0128602 (2015).

4. Yang, G. & Zhou, L. J. Zwitterionic versus canonical amino acids over the various defects in zeolites: a two–layer ONIOM calculation. *Sci. Rep.* **4**, 6594 (2014).

5. Yang, G., Zhou, L. J., Liu, X. C., Han, X. W. & Bao, X. H. Density functional calculations on

the Distribution, acidity, and catalysis of Ti^{IV} and Ti^{III} ions in MCM-22 zeolite. *Chem. Eur. J.* **17**, 1614-621 (2011).

6. Vasconcelos, I. F., Bunker, B. A. & Cygan, R. T. Molecular dynamics modeling of ion adsorption to the basal surfaces of kaolinite. *J. Phys. Chem. C* **111**, 6753-6762. (2007).

7. Frisch, M. J., Trucks, G. W., Schlegel, H. B., Scuseria, G. E., Robb, M. A., Cheeseman, J. R. et al. Gaussian 09, Revision D.01, Gaussian, Inc., Wallingford CT. (2013).

8. Johannesen, Ø. & Andersen, A. G. (Eds.) Selected Topics in High Temperature Chemistry: Defect Chemistry of Solids, Elsevier, B. V. (1989).

9. Yamagishi, K., Namba, S. & Yashima, T. Defect Sites in Highly Siliceous HZSM-5 Zeolites: A Study Performed by Aiumination and IR Spectroscopy. *J. Phys. Chem.* **95**, 872-877(1991).

10. Kawai, T. & Tsutsumi K. Reactivity of Silanol Groups on Zeolite Surfaces. *Colloid Polym. Sci.* **276**, 992-998 (1998).

11. van Bokhoven, J. A., van der Eerden, A. M. J. & Koningsberger, D. K. Three-Coordinate Aluminum in Zeolites Observed with In situ X-ray Absorption Near-Edge Spectroscopy at the Al K-Edge: Flexibility of Aluminum Coordinations in Zeolites. *J. Am. Chem. Soc.* **125**, 7435-7442 (2003).

12. Wu, J. & Buseck, P. R. Carbon storage at defect sites in mantle mineral analogues. *Nature Geosci.* **6**, 875-878 (2013).

13. Greathouse, J. A. & Cygan, R. T. Molecular dynamics simulation of uranyl(VI) adsorption equilibria onto an external montmorillonite surface. *Phys. Chem. Chem. Phys.* **7**, 3580-3586 (2005).

14. Suter, J. L., Coveney, P. V., Greenwell, H. C. & Thyveetil, M. A. Large-scale molecular dynamics study of montmorillonite clay: emergence of undulatory fluctuations and determination of material properties. *J. Phys. Chem. C* **111**, 8248-8259 (2007).

15. Vasconcelos, I. F., Bunker, B. A. & Cygan, R. T. Molecular dynamics modeling of ion adsorption to the basal surfaces of kaolinite. *J. Phys. Chem. C* **111**, 6753-6762 (2007).

16. Du, H. & Miller, J. D. Adsorption states of amphipathic solutes at the surface of naturally hydrophobic minerals: a molecular dynamics simulation study. *Langmuir* **23**, 11587-11596 (2007).

17. Mazo, M. A. et al. Molecular dynamics simulation of thermomechanical properties of montmorillonite crystal. 1. isolated clay nanoplate. *J. Phys. Chem. B* **112**, 2964-2969 (2008).
18. Cheng, T & Sun, H. Adsorption of ethanol vapor on mica surface under different relative humidities: A molecular simulation study. *J. Phys. Chem. C* **116**, 16436-16446 (2012).
19. Shapley, T. V., Molinari, M., Zhu, R. & Parker, S. C. Atomistic modeling of the sorption free energy of dioxins at clay-water interfaces. *J. Phys. Chem. C* **117**, 24975-24984 (2013).
20. Ngouana, B. F. W. & Kalinichev, A. G. Structural arrangements of isomorphic substitutions in smectites: molecular simulation of the swelling properties, interlayer structure, and dynamics of hydrated Cs-montmorillonite revisited with new clay models. *J. Phys. Chem. C* **118**, 12758-12773 (2014).

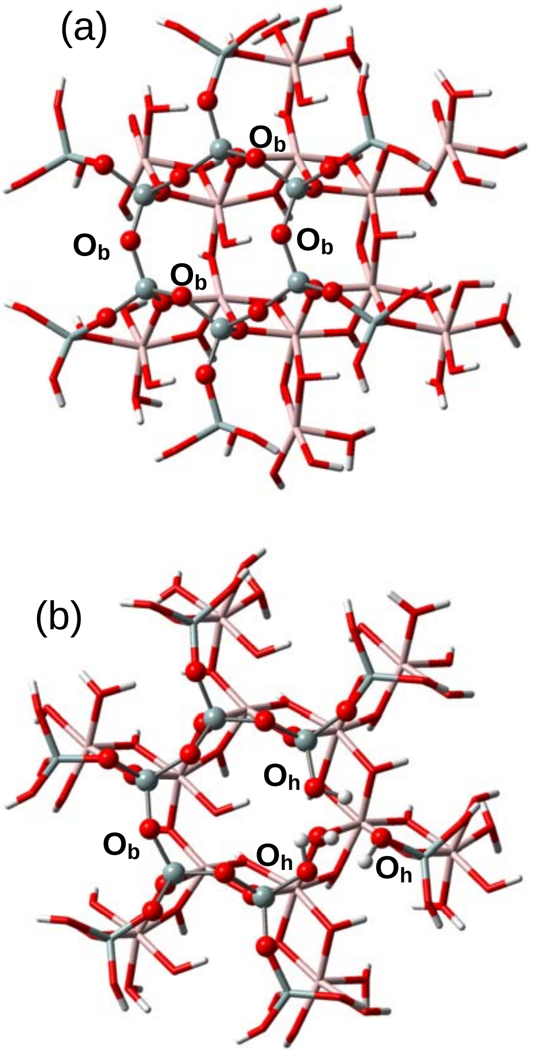

Figure S1. Cluster models for (a) regular kaolinite (b) kaolinite with **Si_1** defect site.

The Si, O, H and Al atoms are displayed in cyan, red, white and rose pink, respectively.

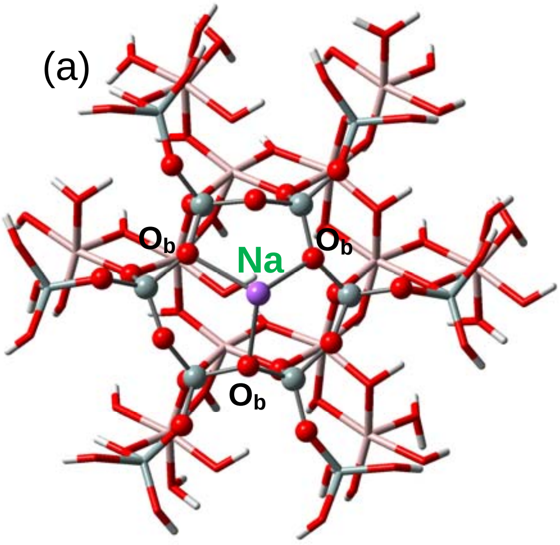

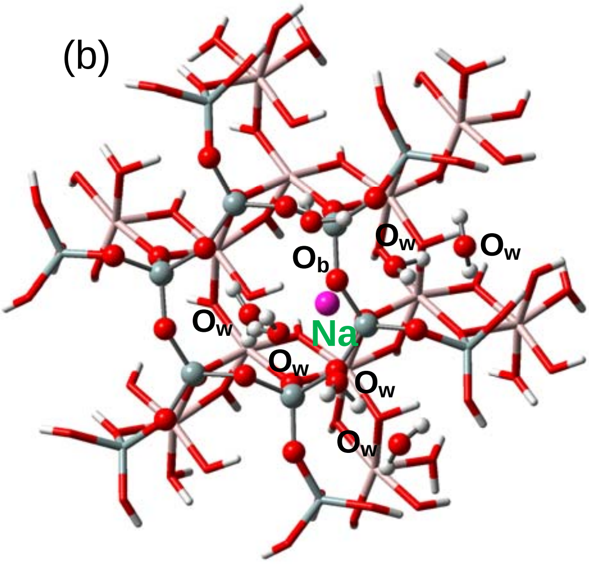

Figure S2. Cluster models for (a) Na^+ adsorption on the tetrahedral SiO_4 surface of regular kaolinite and (b) addition of seven water molecules.

The Si, O, H, Al, and Na atoms are displayed in cyan, red, white, rose pink and purple, respectively.

Table S1. Parameters for the CLAYFF force field [a]

symbol	species	q (e)	ε (kcal/mol)	σ (Å)	source
ao	octahedral aluminum	+1.575	1.3298×10^{-6}	4.7949	[59]
st	tetrahedral silicon	+2.1	1.8405×10^{-6}	3.7064	[59]
ob	bridging oxygen	-1.05	0.1554	3.5532	[59]
oh	hydroxyl oxygen	-0.95	0.1554	3.5532	[59]
ho	hydroxyl hydrogen	+0.425			[59]
Na	aqueous Na^+ ion	+1.0	0.1301	2.6378	[59]
Pb	aqueous Pb^{2+} ion	+2.0	0.1182	3.3243	[27]
h*	water hydrogen	+0.41			[60]
o*	water oxygen	-0.82	0.1554	3.1655	[60]
	Bond stretch [b]	k_1 (kcal/mol Å^2)	r_0 (Å)		
	oh–ho	554.1349	1.0		[59]
	o*–h*	554.1349	1.0		[60]
	Angle bend [c]	k_2 (kcal/mol rad^2)	θ (deg)		
	h*–o*–h*	45.7696	109.47		[60]

[a] q is partial charge, σ is the finite distance at which the inter-particle L-J potential equals zero and ε is the well depth of Lennard-Jones potential;

[b] k_1 is the harmonic potential constant and r_0 is the equilibrium bond length;

[c] k_2 is the harmonic angle potential and θ is the equilibrium angle.

Table S2. Numbers of Na^+ and Pb^{2+} ions falling with the specified RMSF ranges for 0.96 mol/L NaCl and $PbCl_2$ solutions in contact with kaolinite containing different numbers of $\mathbf{S_1}$ defect sites [a]

RMSF(Å)	Na^+				Pb^{2+}			
	$\mathbf{Si_0}$	$\mathbf{Si_1(9)}$	$\mathbf{Si_1(18)}$	$\mathbf{Si_1(27)}$	$\mathbf{Si_0}$	$\mathbf{Si_1(9)}$	$\mathbf{Si_1(18)}$	$\mathbf{Si_1(27)}$
≤ 1.2	1	18	25	32	0	3	4	4
1.2 ~ 1.7	8	16	18	18	3	8	19	22
1.7 ~ 2.7	28	22	21	16	36	43	40	39
> 2.7	47	28	20	18	45	30	21	19

[a] Numbers of $\mathbf{Si_1}$ defect sites are given in parentheses, and the default is 9.

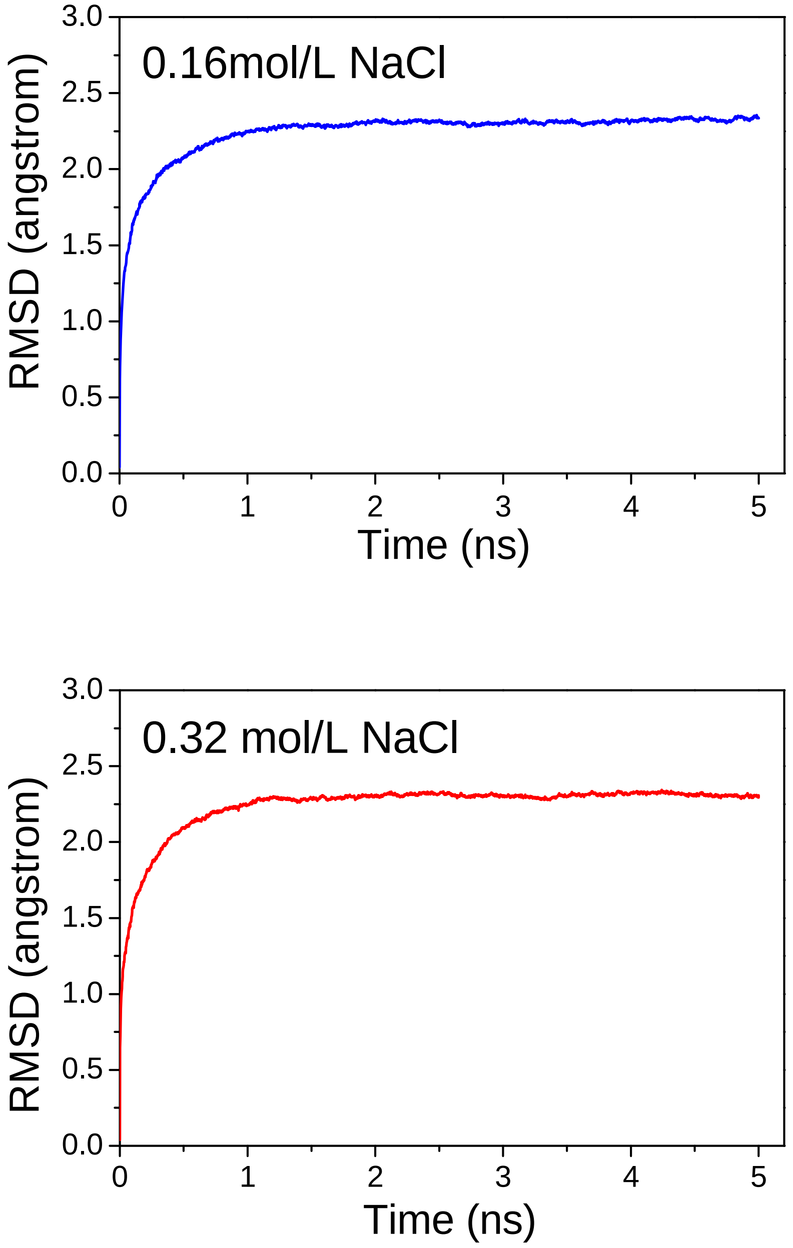
0.16mol/L NaCl
RMSD (angstrom)
Time (ns)
0.32 mol/L NaCl
RMSD (angstrom)
Time (ns)
0.0
0.5
1.0
1.5
2.0
2.5
3.0
0
1
2
3
4
5

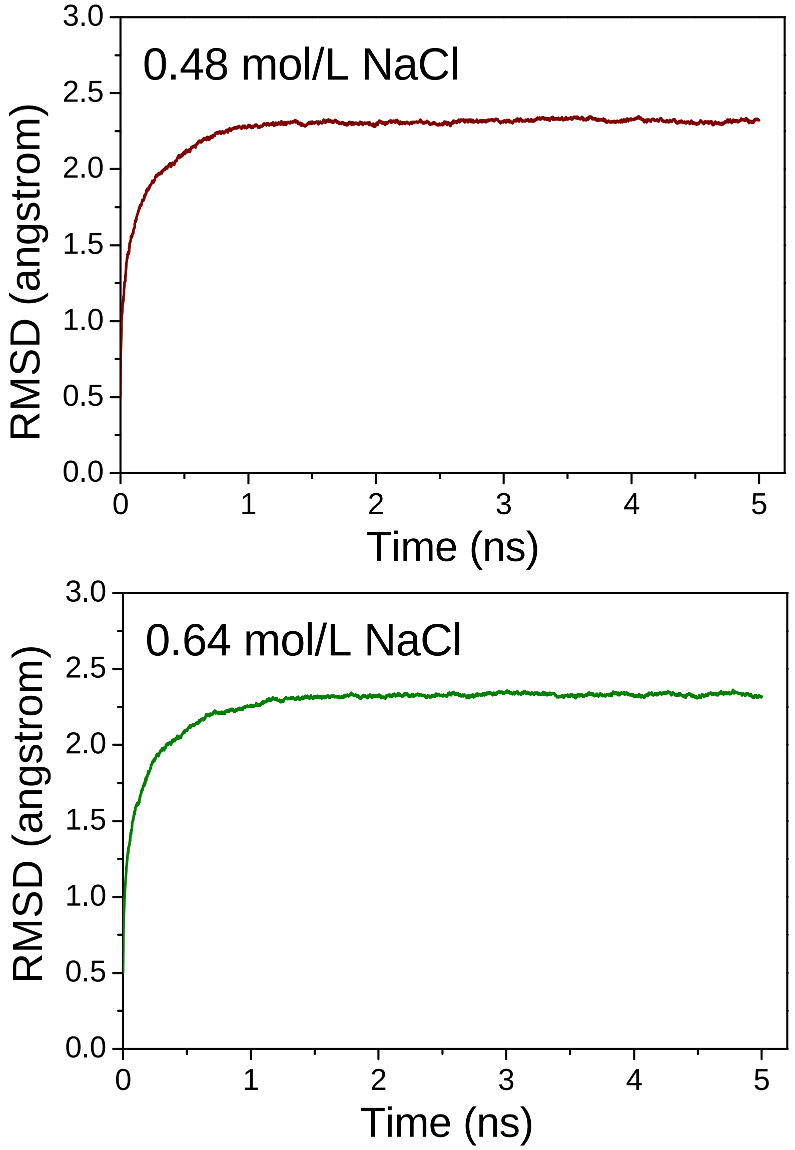

0.48 mol/L NaCl
RMSD (angstrom)
3.0
2.5
2.0
1.5
1.0
0.5
0.0
0
1
2
3
4
5
Time (ns)
0.64 mol/L NaCl
RMSD (angstrom)
3.0
2.5
2.0
1.5
1.0
0.5
0.0
0
1
2
3
4
5
Time (ns)

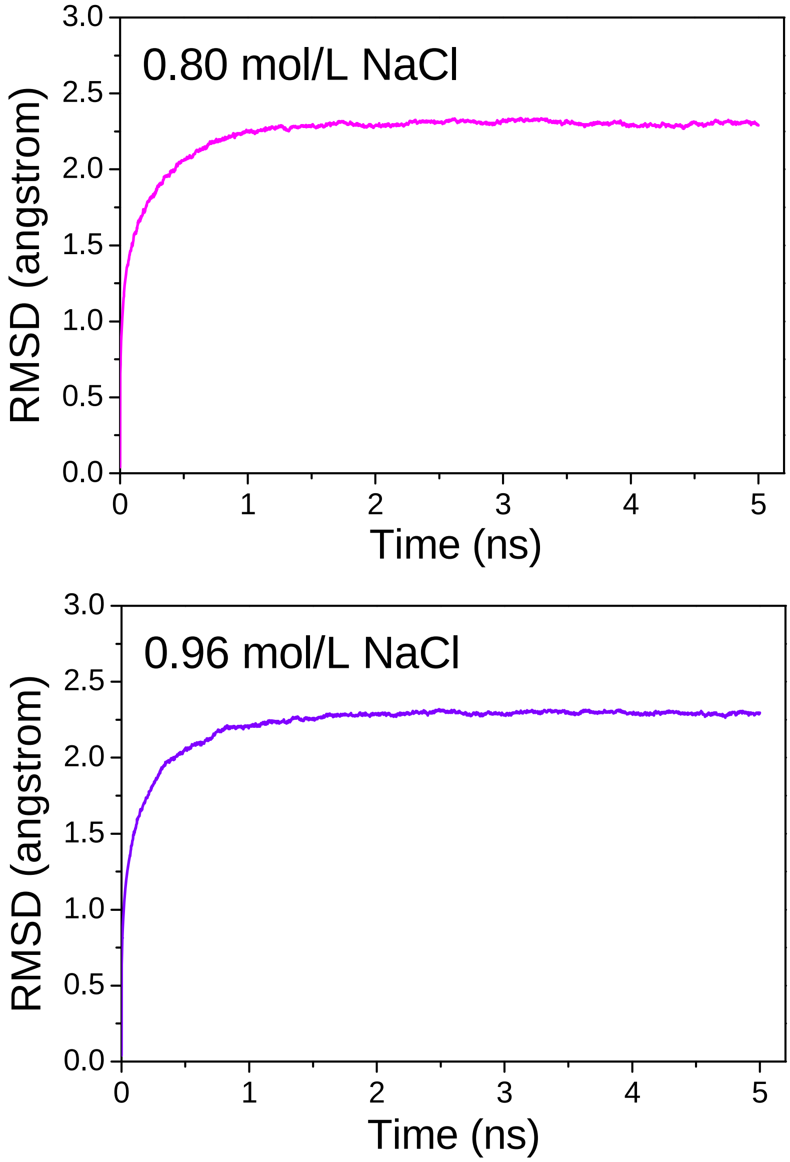

Figure S3. RMSD plots for MD simulations of regular kaolinite in contact with NaCl solutions of different ionic concentrations (Ionic concentrations are indicated in the legends).

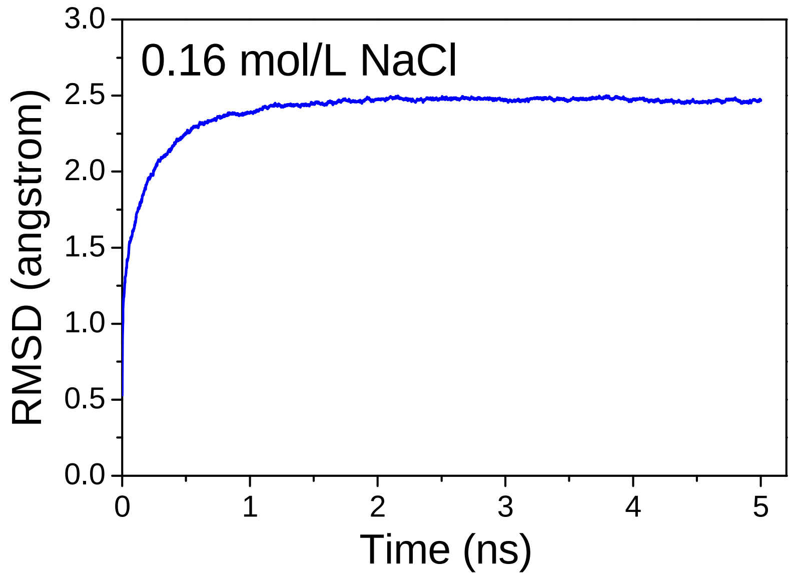
0.16 mol/L NaCl
RMSD (angstrom)
Time (ns)
0.0
0.5
1.0
1.5
2.0
2.5
3.0
0
1
2
3
4
5

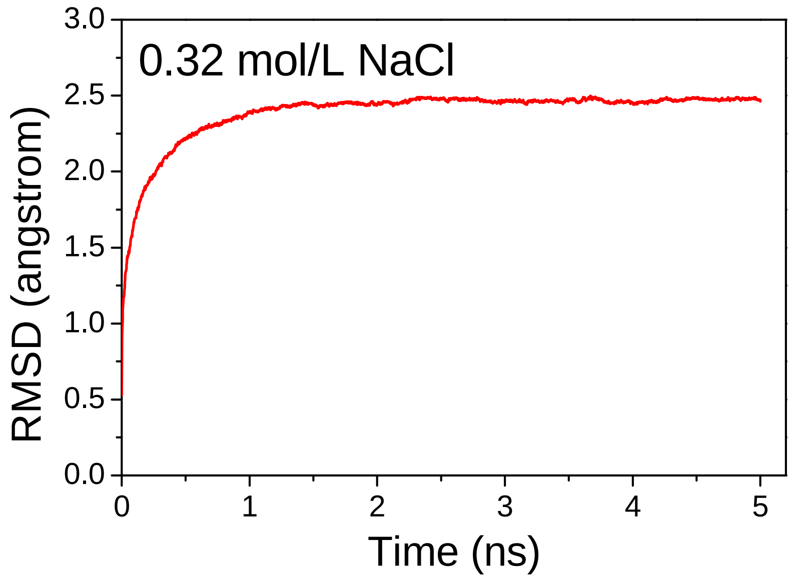
0.32 mol/L NaCl
RMSD (angstrom)
Time (ns)
0.0
0.5
1.0
1.5
2.0
2.5
3.0
0
1
2
3
4
5

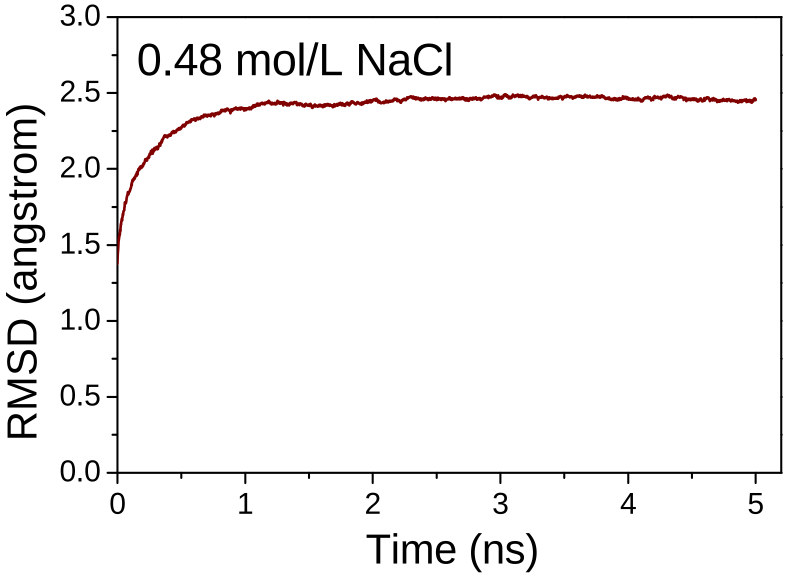
0.48 mol/L NaCl
RMSD (angstrom)
Time (ns)
0.0
0.5
1.0
1.5
2.0
2.5
3.0
0
1
2
3
4
5

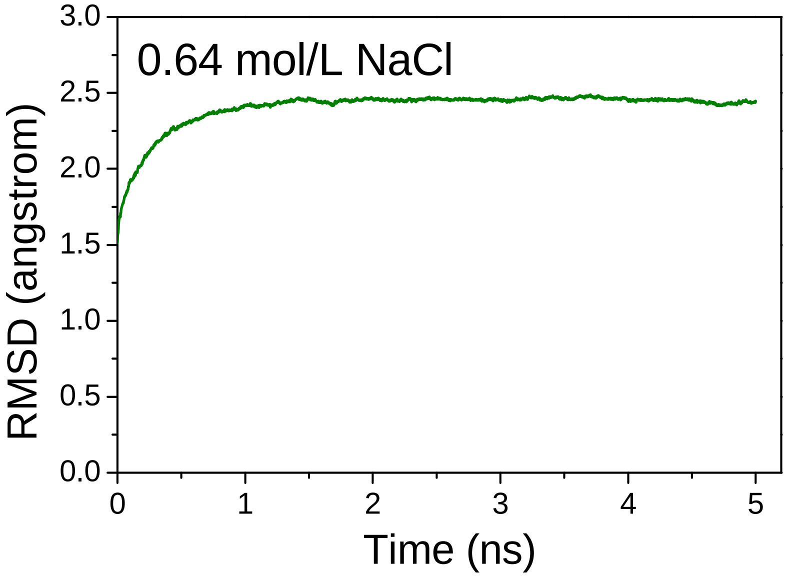
0.64 mol/L NaCl
RMSD (angstrom)
Time (ns)
0.0
0.5
1.0
1.5
2.0
2.5
3.0
0
1
2
3
4
5

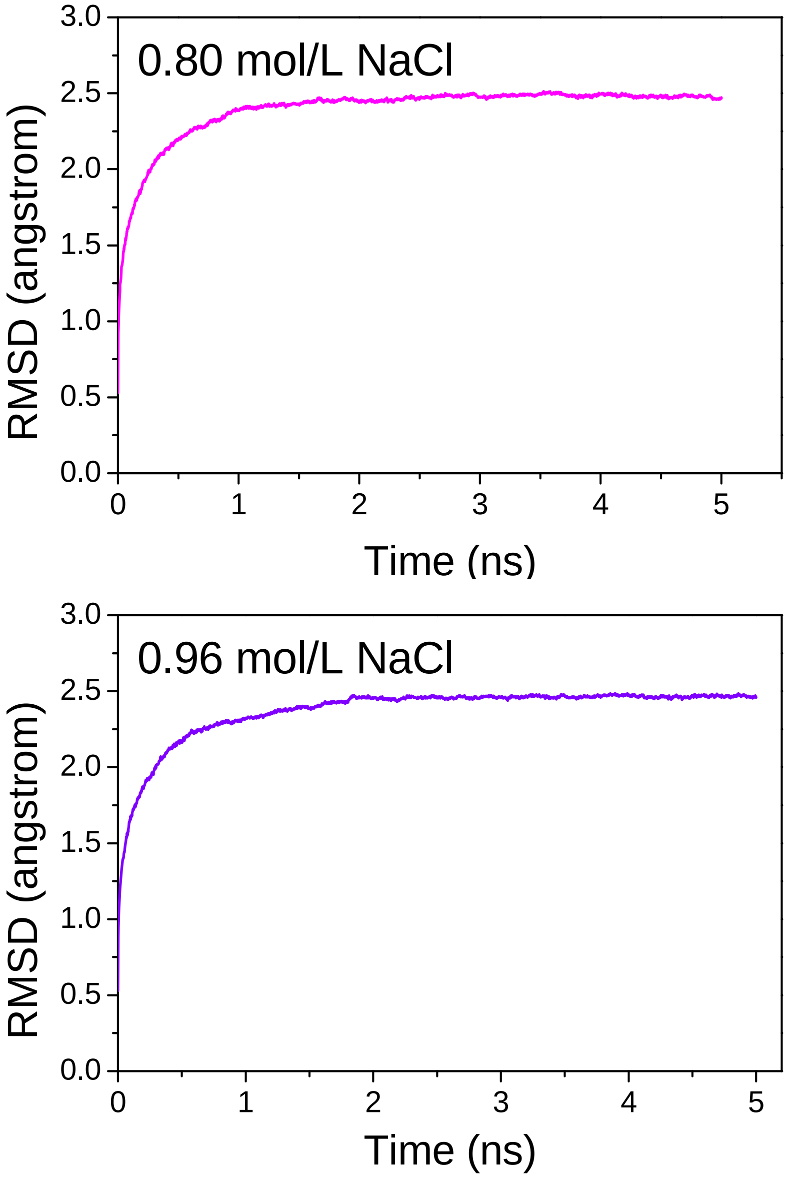

Figure S4. RMSD plots for MD simulations of defective kaolinite (**Si_1**) in contact with NaCl solutions of different ionic concentrations (Ionic concentrations are indicated in the legends).

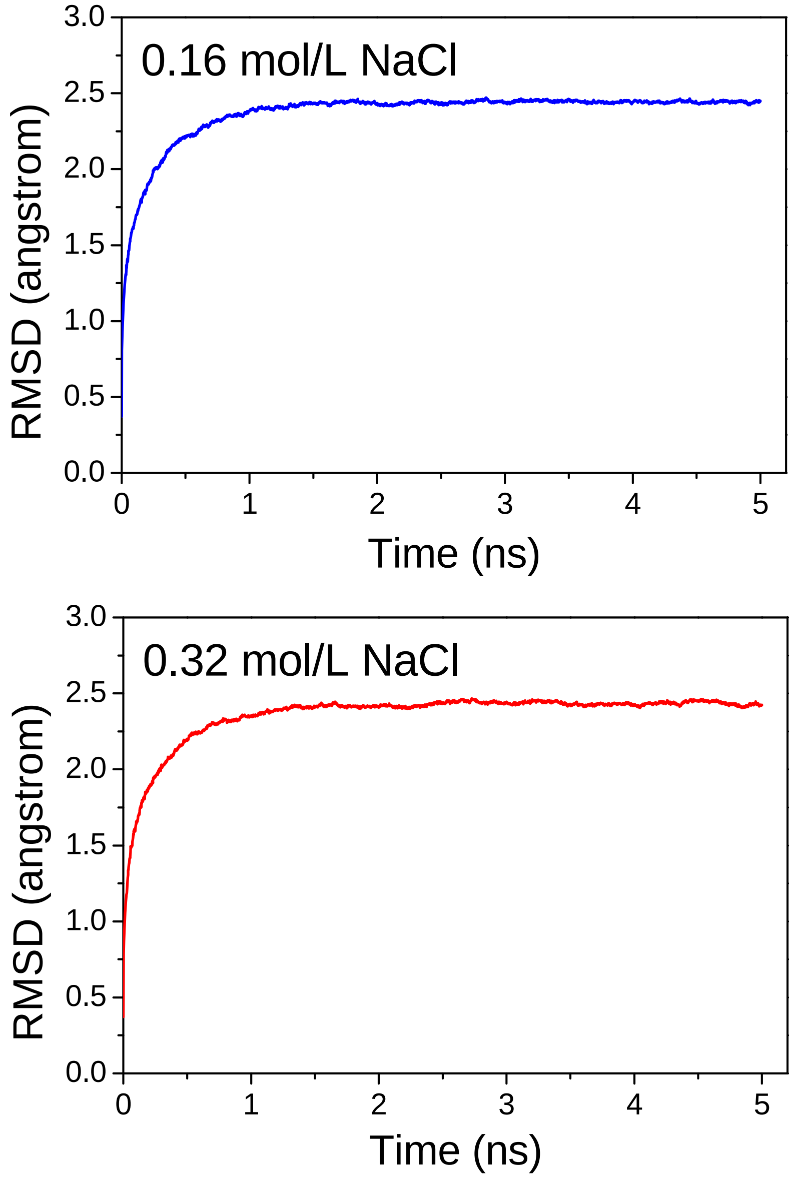
0.16 mol/L NaCl
RMSD (angstrom)
Time (ns)
0.32 mol/L NaCl
RMSD (angstrom)
Time (ns)

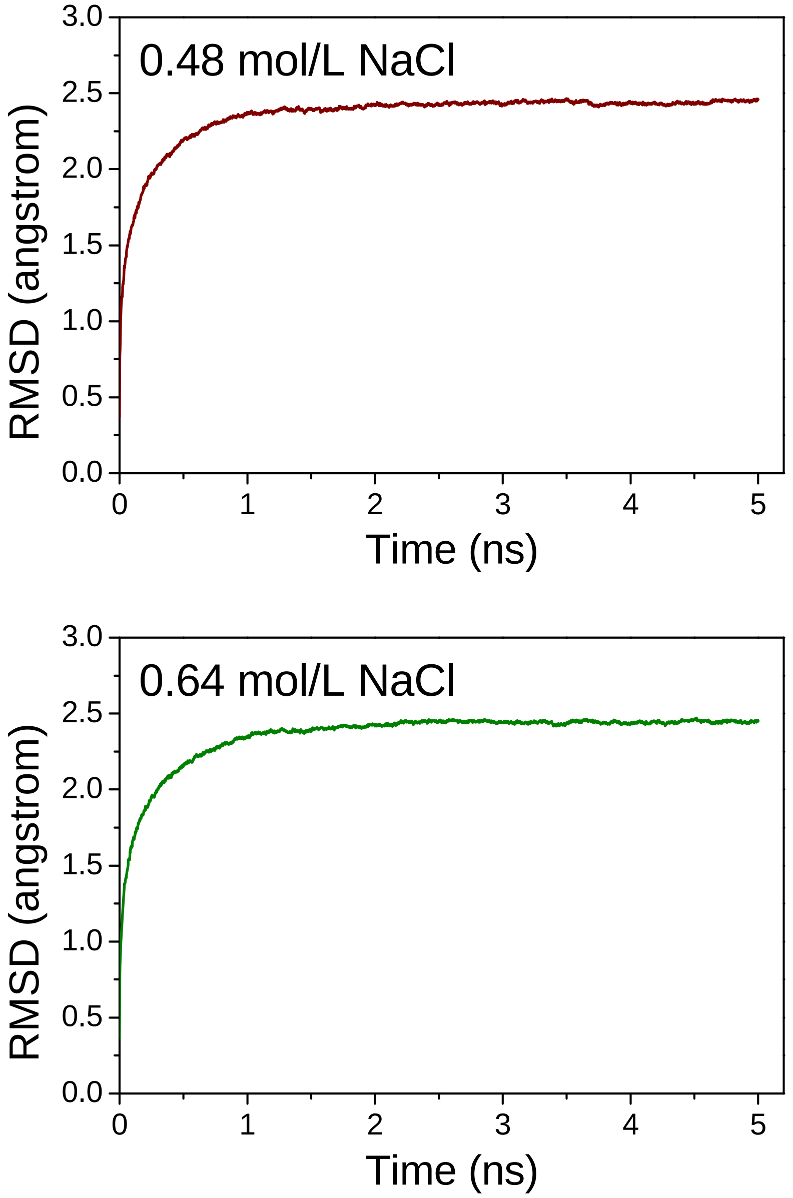
0.48 mol/L NaCl
RMSD (angstrom)
Time (ns)
0.0
0.5
1.0
1.5
2.0
2.5
3.0
0
1
2
3
4
5
0.64 mol/L NaCl
RMSD (angstrom)
Time (ns)

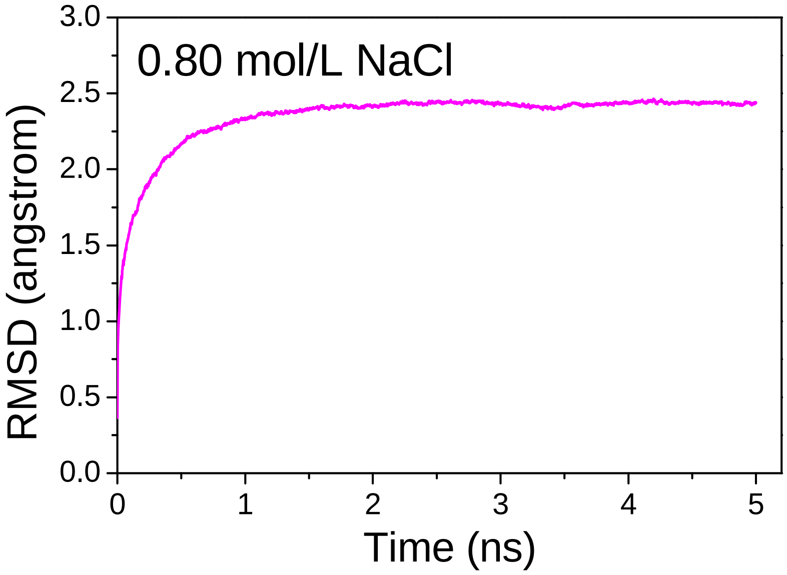

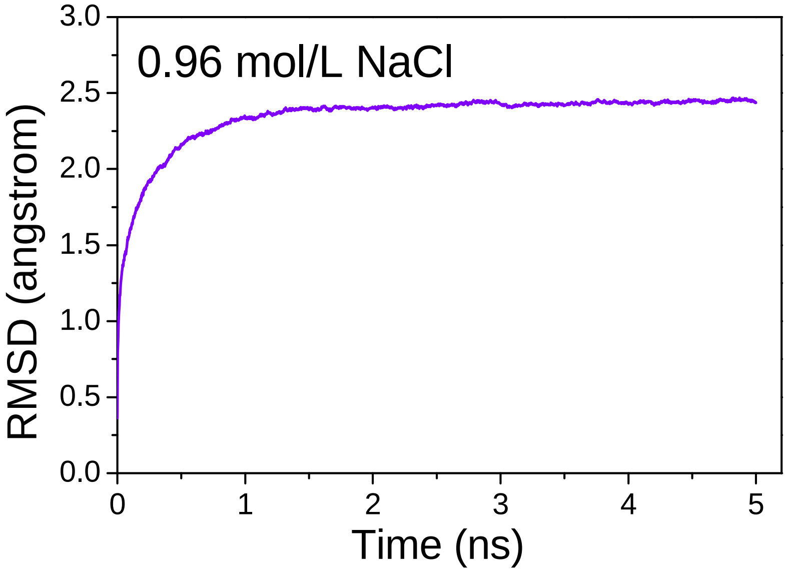

Figure S5. RMSD plots for MD simulations of defective kaolinite (**Si_2**) in contact with NaCl solutions of different ionic concentrations (Ionic concentrations are indicated in the legends).

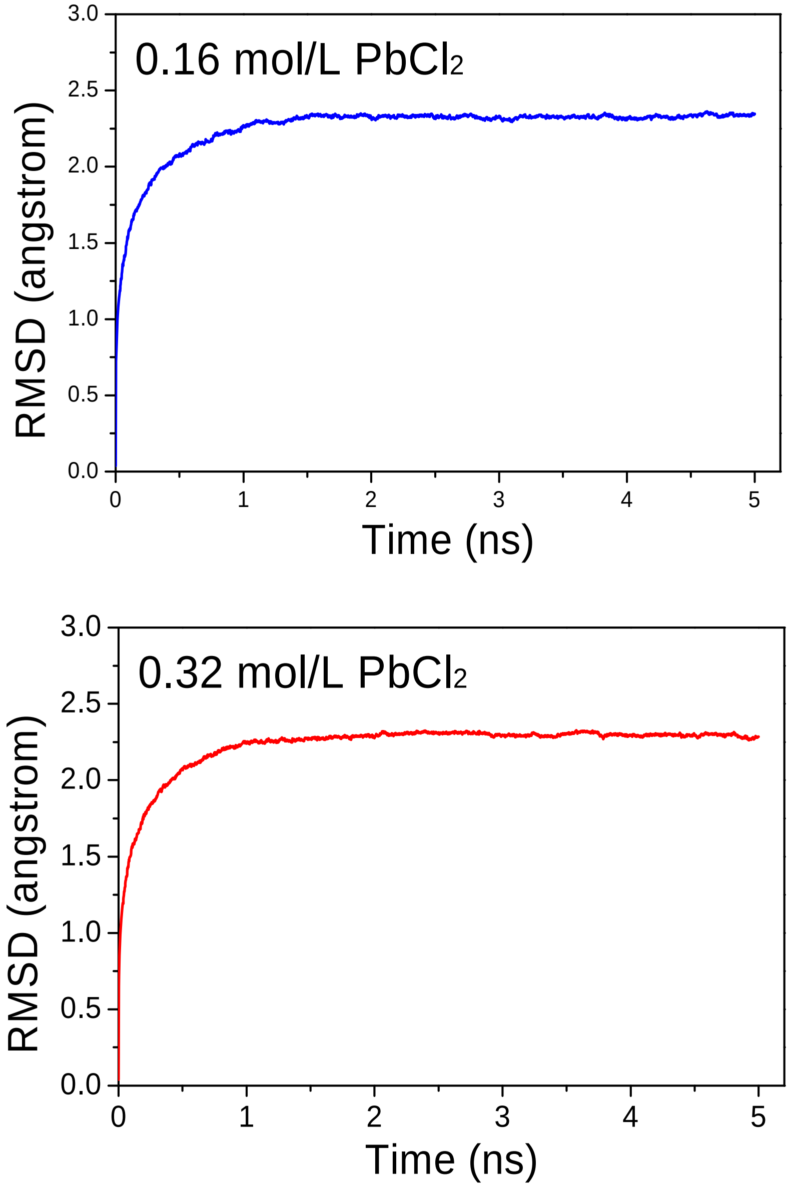
0.16 mol/L PbCl2
RMSD (angstrom)
Time (ns)
0.32 mol/L PbCl2
RMSD (angstrom)
Time (ns)

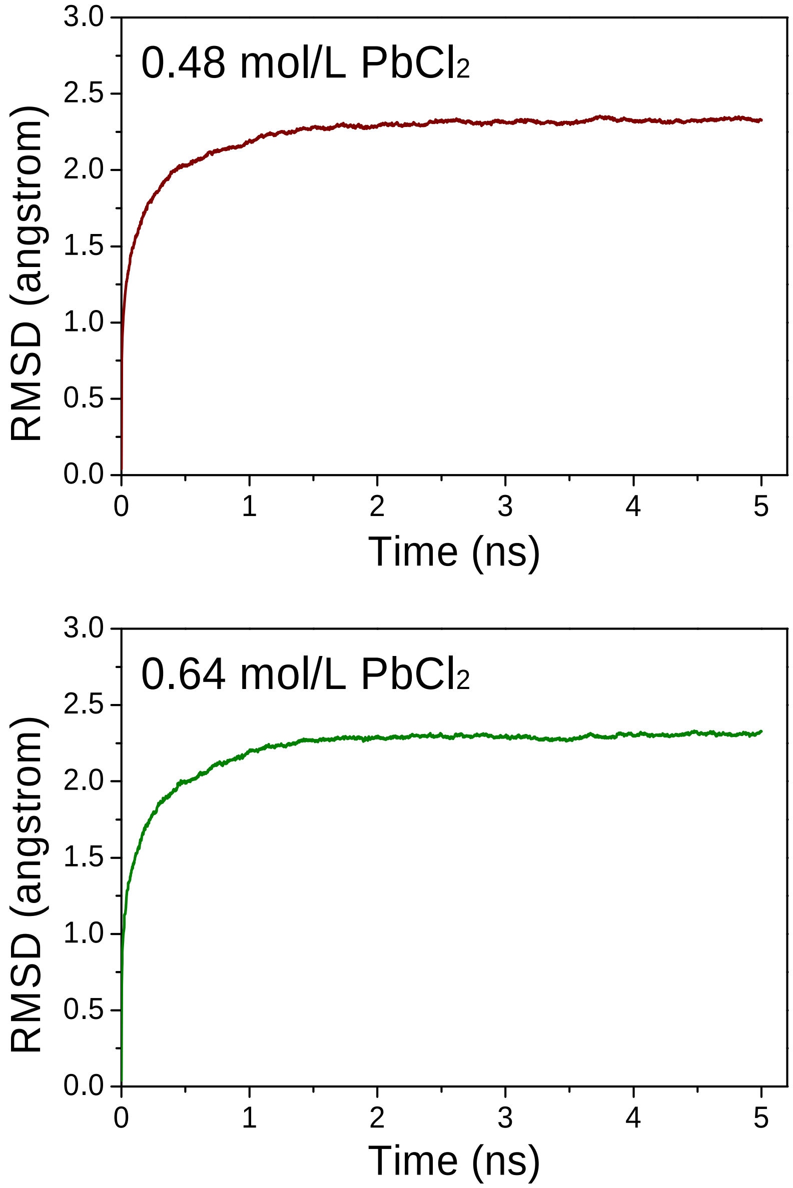
0.48 mol/L PbCl2
RMSD (angstrom)
Time (ns)
0.64 mol/L PbCl2
RMSD (angstrom)
Time (ns)

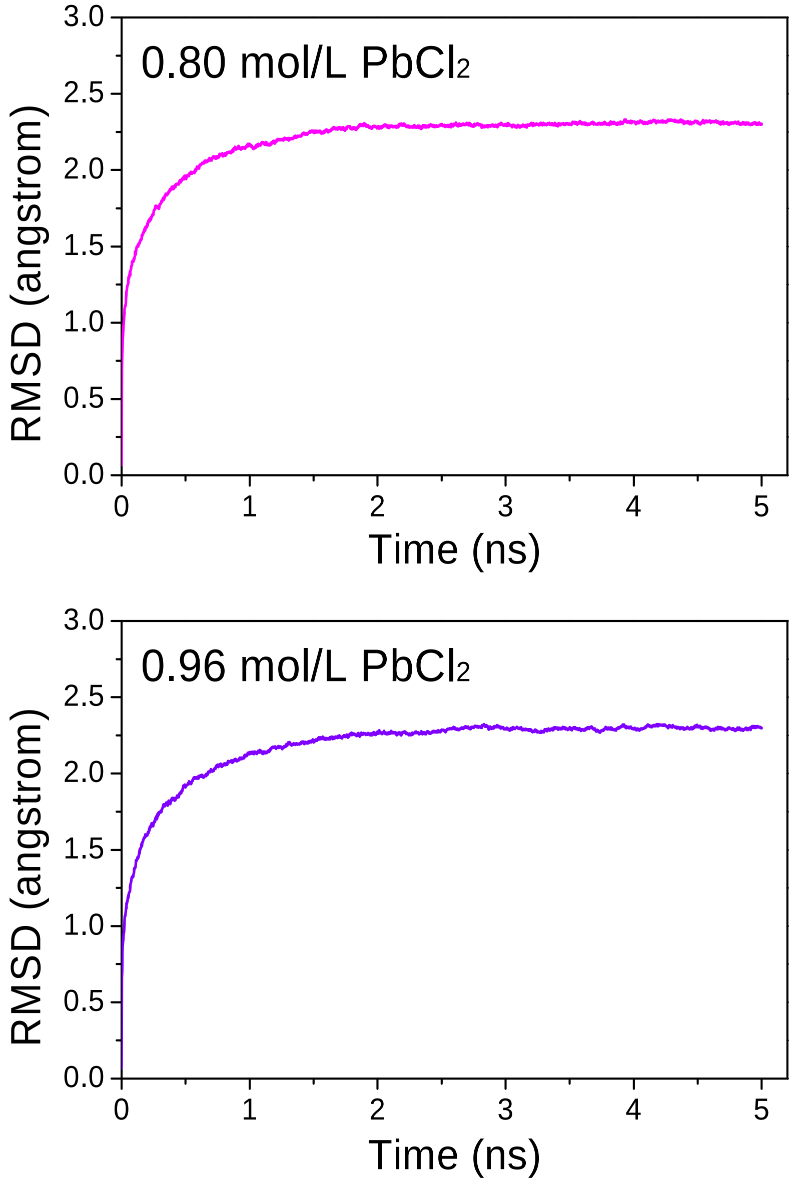

Figure S6. RMSD plots for MD simulations of regular kaolinite (**Si_0**) in contact with $PbCl_2$ solutions of different ionic concentrations (Ionic concentrations are indicated in the legends).

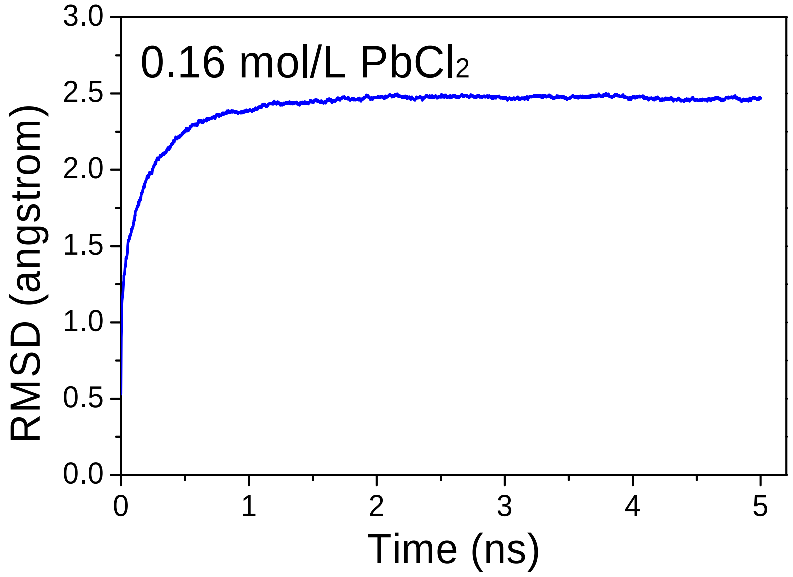
0.16 mol/L PbCl2
RMSD (angstrom)
Time (ns)
0.0
0.5
1.0
1.5
2.0
2.5
3.0
0
1
2
3
4
5

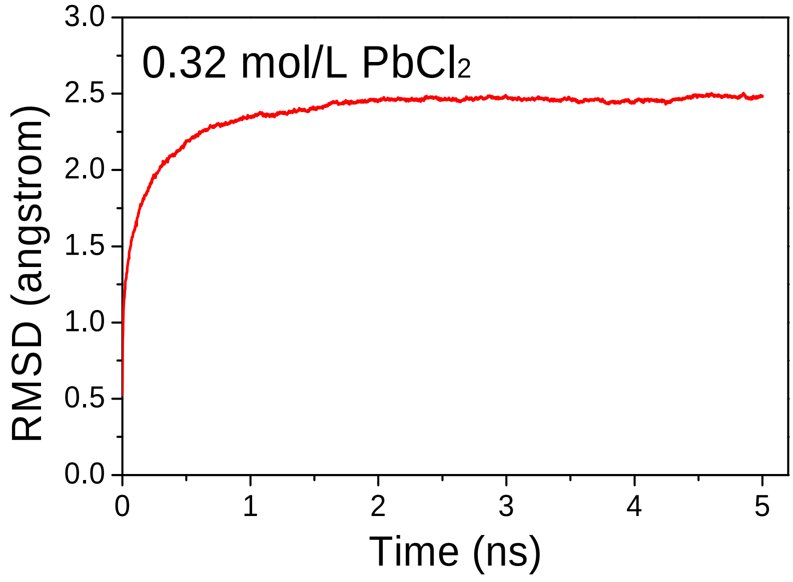
0.32 mol/L PbCl2
RMSD (angstrom)
Time (ns)
0.0
0.5
1.0
1.5
2.0
2.5
3.0
0
1
2
3
4
5

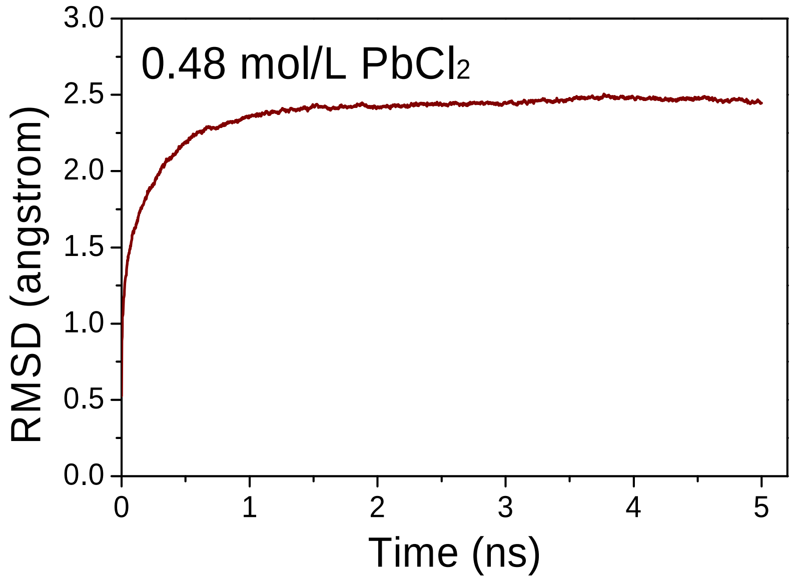
0.48 mol/L PbCl2
RMSD (angstrom)
Time (ns)
0.0
0.5
1.0
1.5
2.0
2.5
3.0
0
1
2
3
4
5

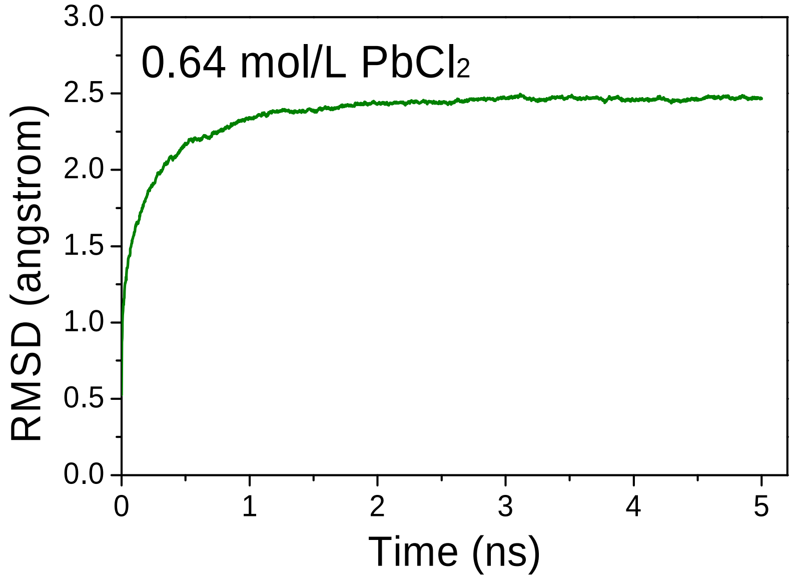
0.64 mol/L PbCl2
RMSD (angstrom)
Time (ns)
0.0
0.5
1.0
1.5
2.0
2.5
3.0
0
1
2
3
4
5

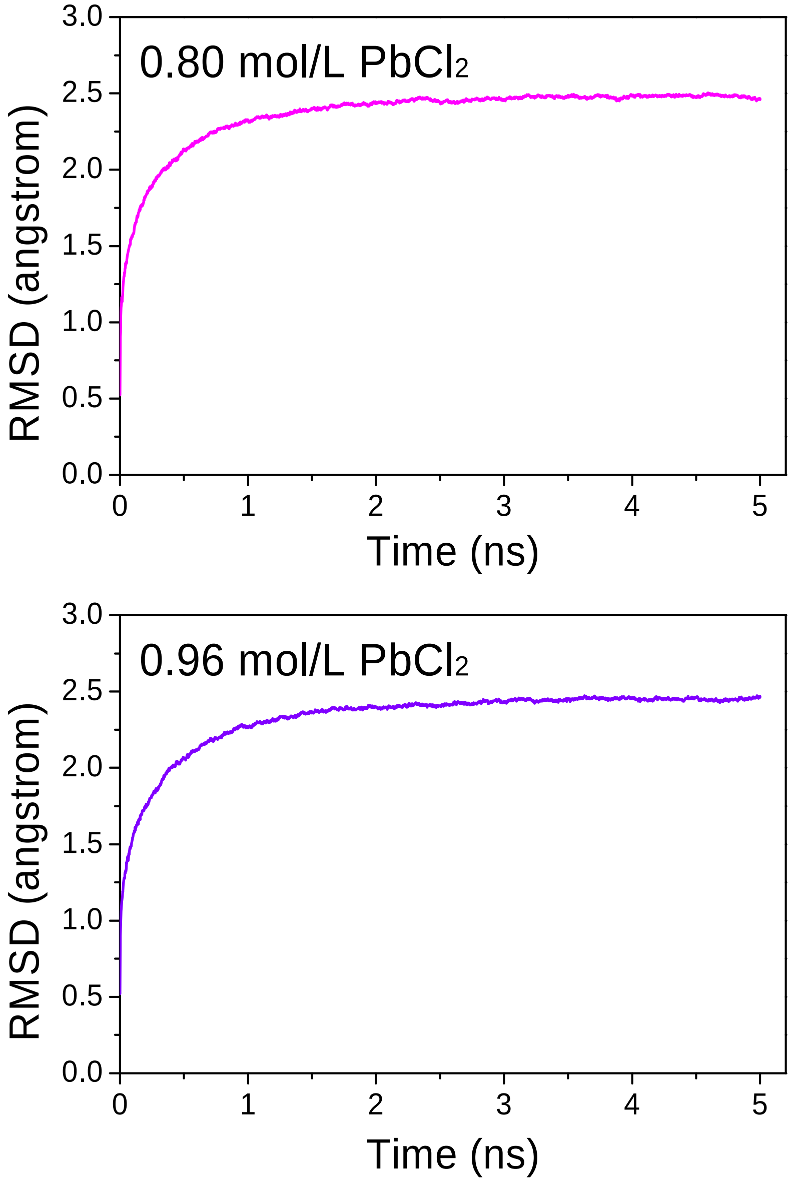

Figure S7. RMSD plots for MD simulations of defective kaolinite (**Si$_1$**) in contact with $PbCl_2$ solutions of different ionic concentrations (Ionic concentrations are indicated in the legends).

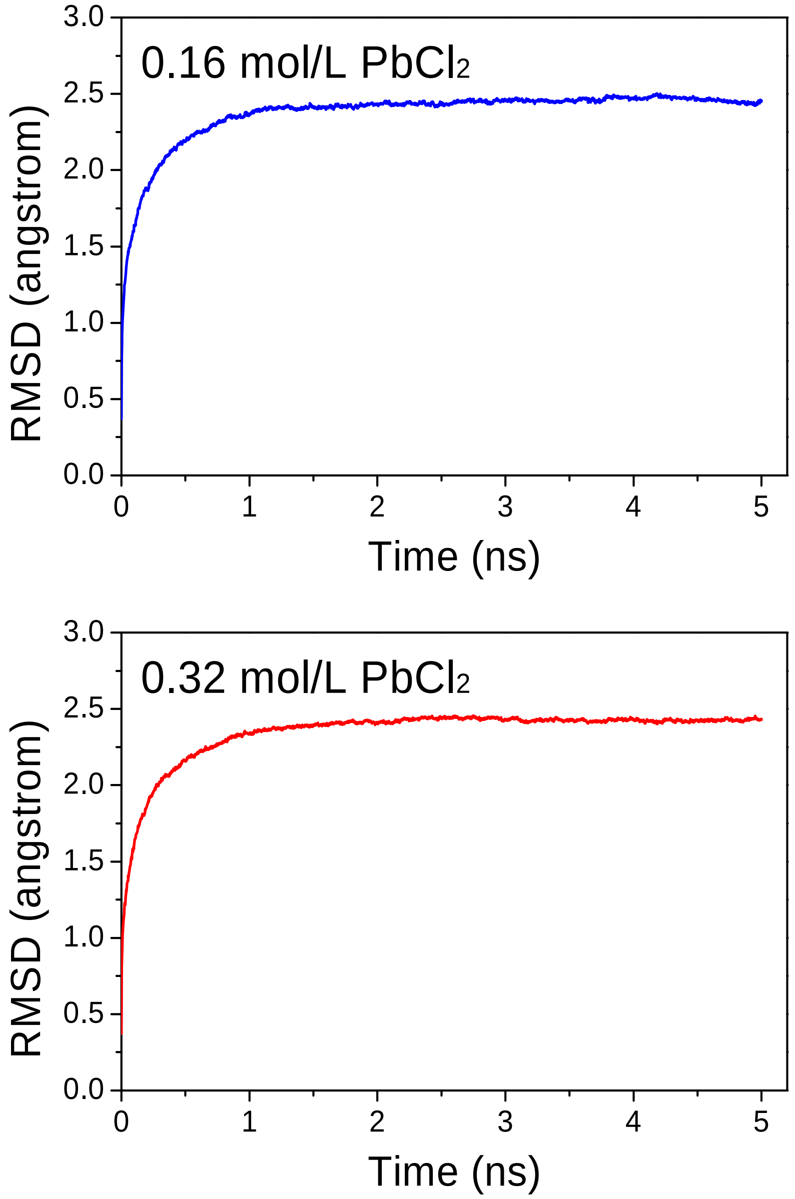
0.16 mol/L PbCl2
RMSD (angstrom)
Time (ns)
0.32 mol/L PbCl2
RMSD (angstrom)
Time (ns)

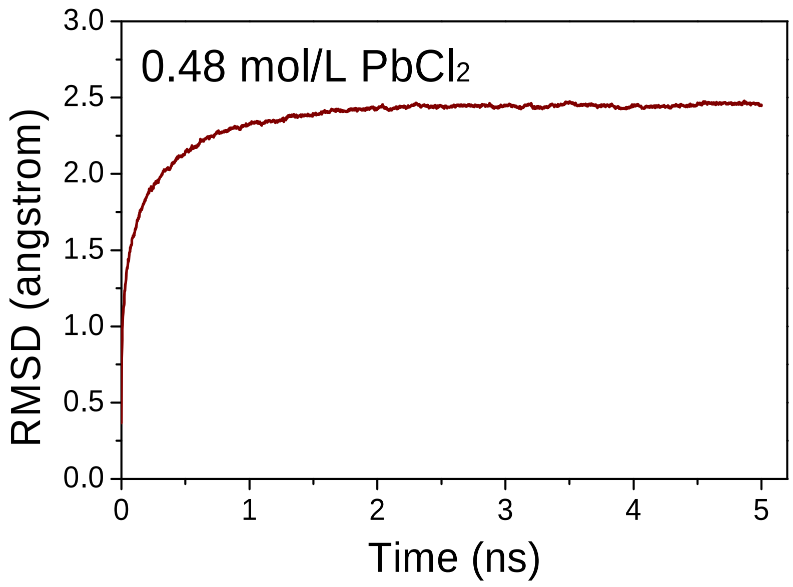
0.48 mol/L PbCl2
RMSD (angstrom)
Time (ns)
0.0
0.5
1.0
1.5
2.0
2.5
3.0
0
1
2
3
4
5

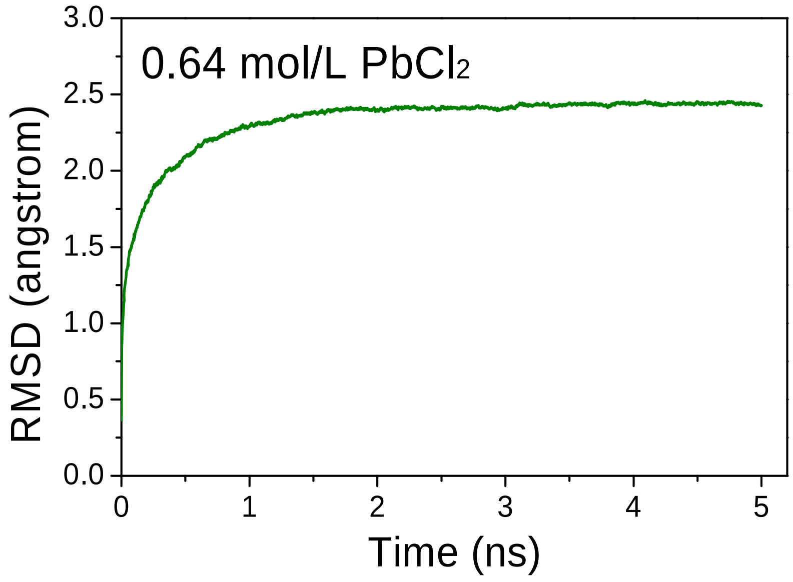
0.64 mol/L PbCl2
RMSD (angstrom)
Time (ns)
0.0
0.5
1.0
1.5
2.0
2.5
3.0
0
1
2
3
4
5

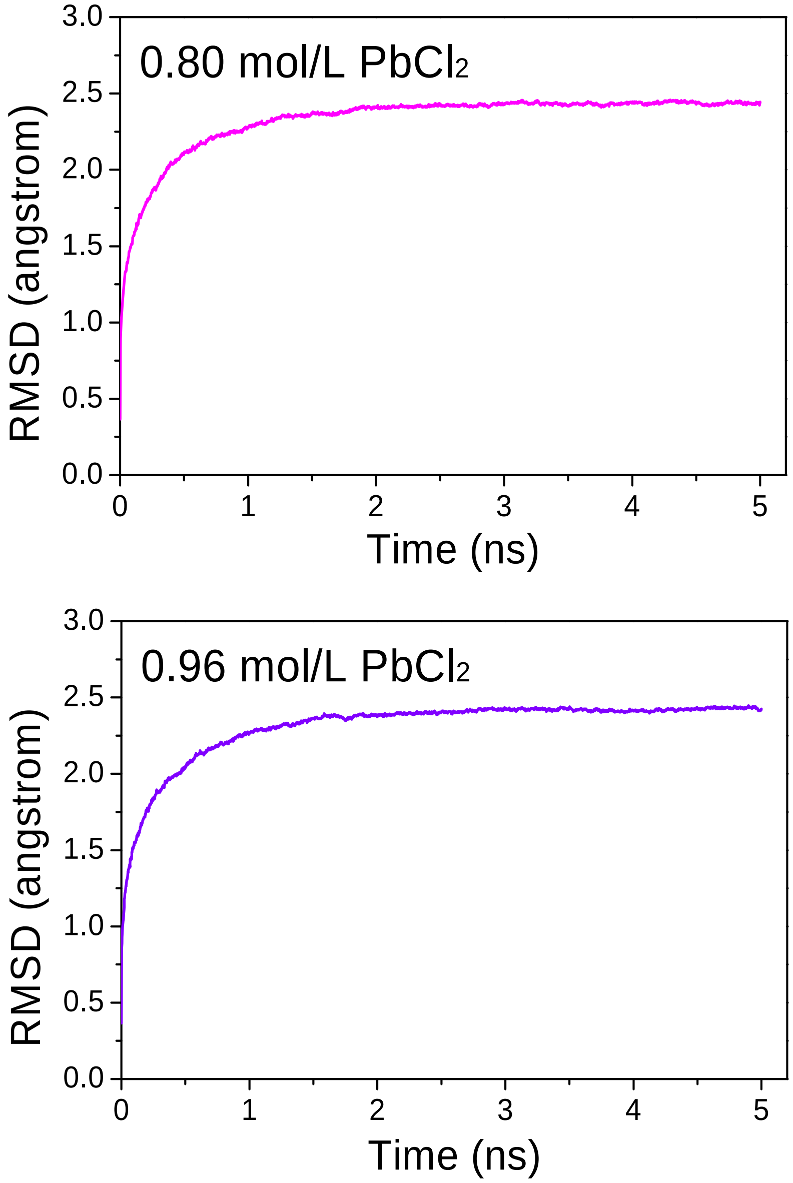

Figure S8. RMSD plots for MD simulations of defective kaolinite (**Si_2**) in contact with $PbCl_2$ solutions of different ionic concentrations (Ionic concentrations are indicated in the legends).

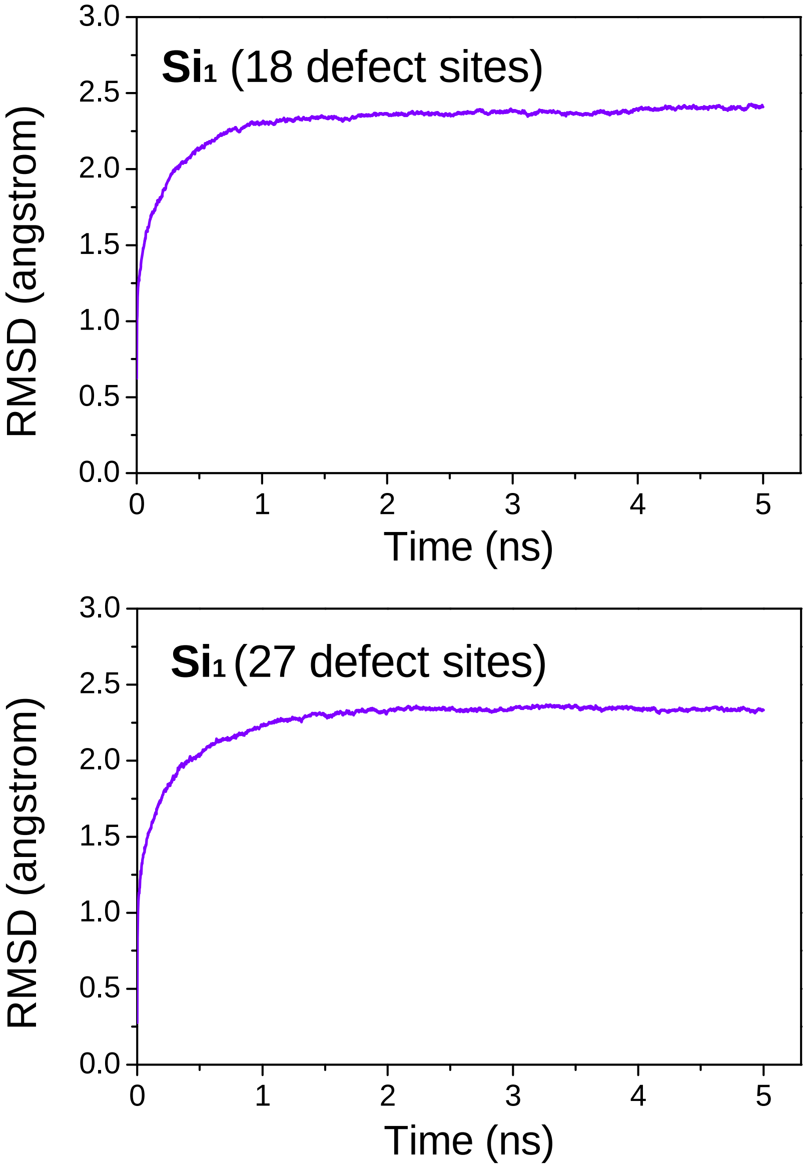

Figure S9. RMSD plots for MD simulations of defective kaolinite (**Si_1**) in contact with 0.96 mol/L NaCl solutions (Numbers of defect sites are indicated in the legends).

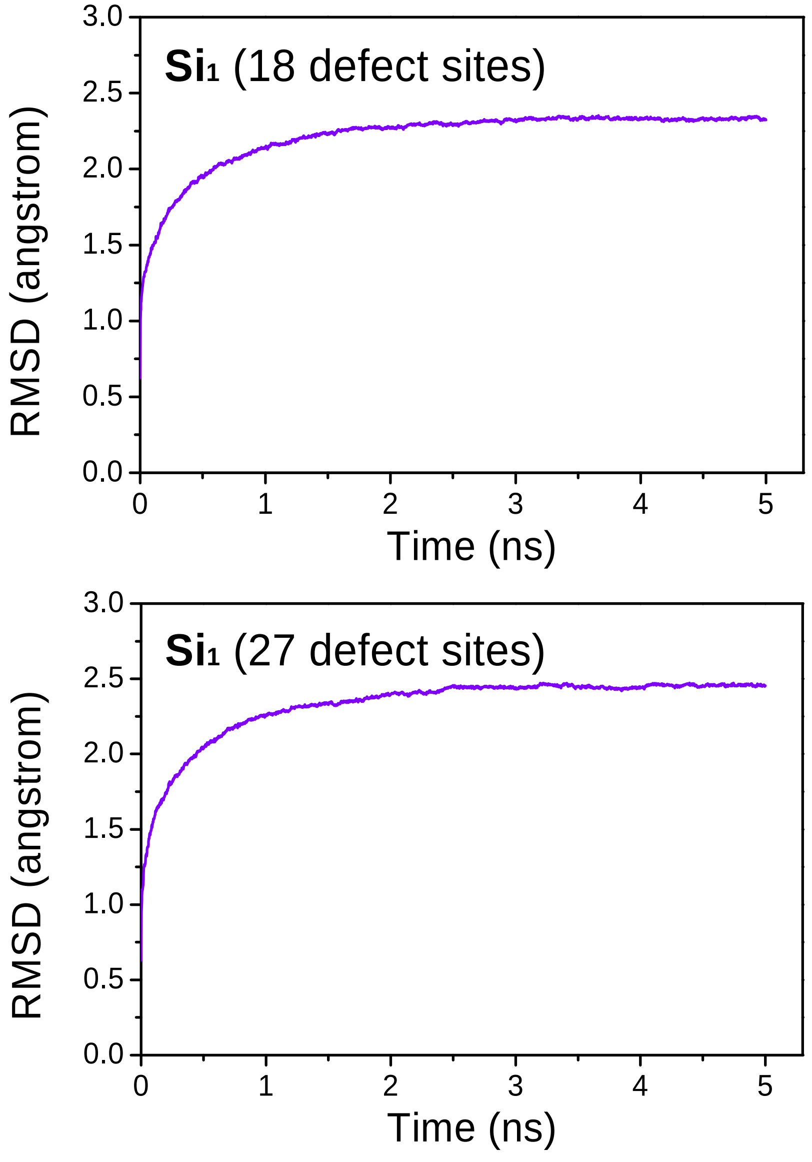

Figure S10. RMSD plots for MD simulations of defective kaolinite (**Si_1**) in contact with 0.96 mol/L $PbCl_2$ solutions (Numbers of defect sites are indicated in the legends).

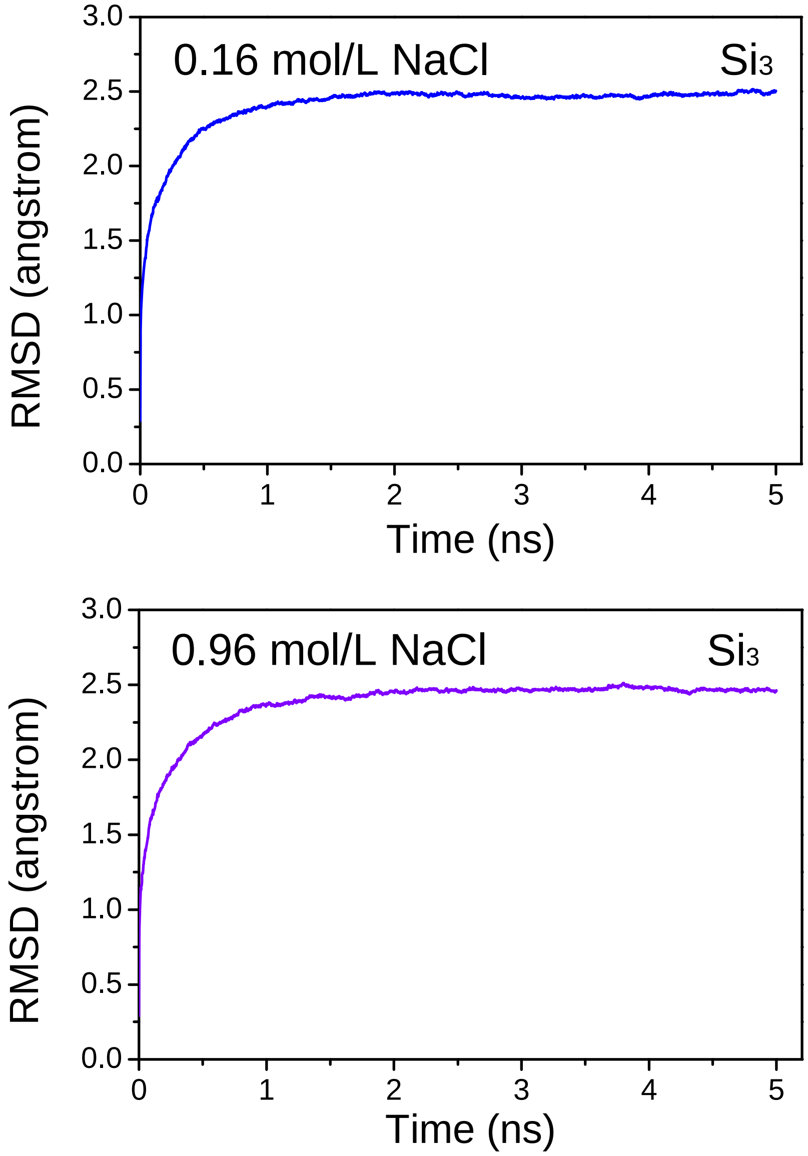

0.16 mol/L NaCl
Si3
RMSD (angstrom)
Time (ns)
0.96 mol/L NaCl
Si3
RMSD (angstrom)
Time (ns)

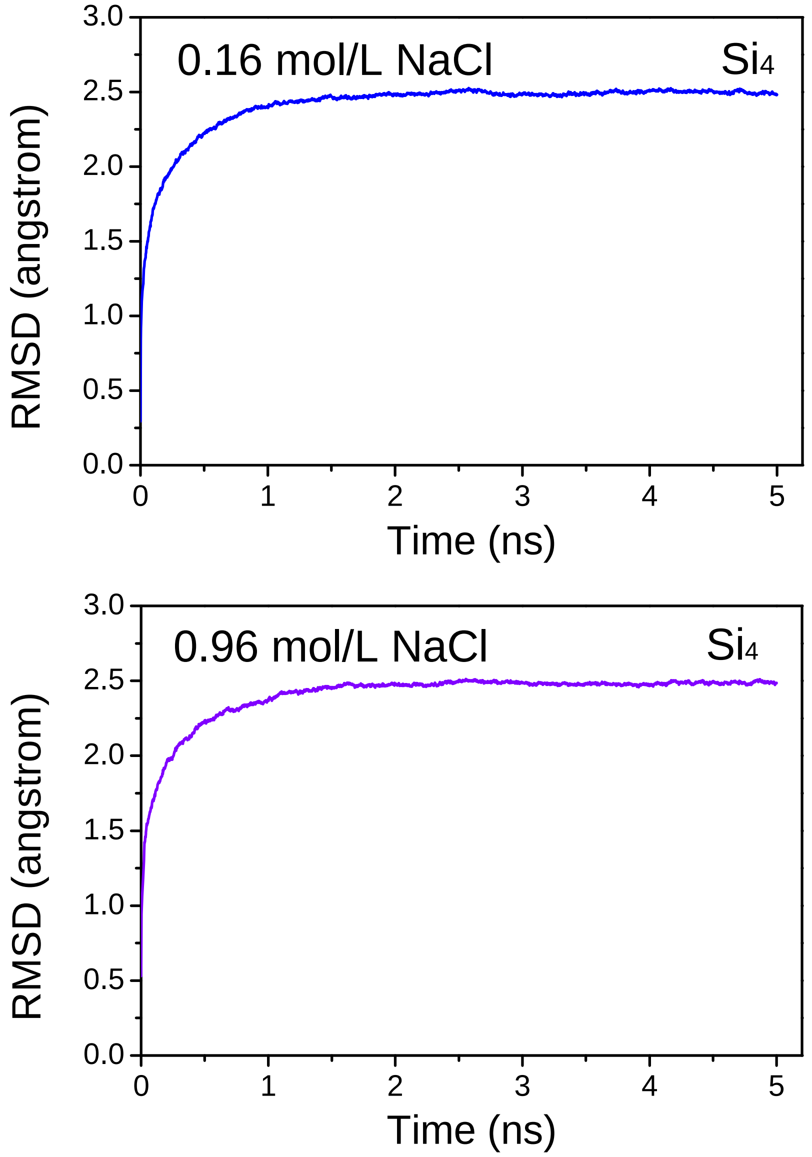

Figure S11. RMSD plots for MD simulations of defective kaolinite (**Si_3** and **Si_4**) in contact with 0.16 and 0.96 mol/L NaCl solutions.

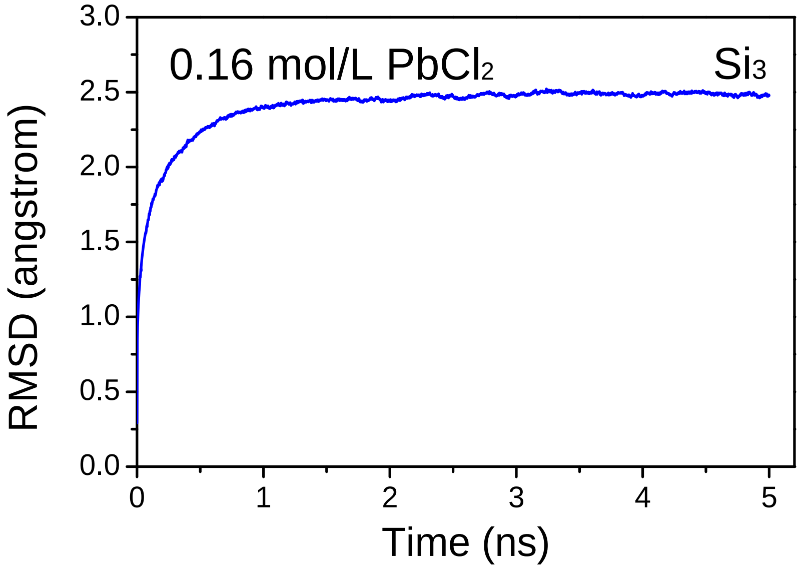
0.16 mol/L PbCl2
Si3
RMSD (angstrom)
Time (ns)
3.0
2.5
2.0
1.5
1.0
0.5
0.0
0
1
2
3
4
5

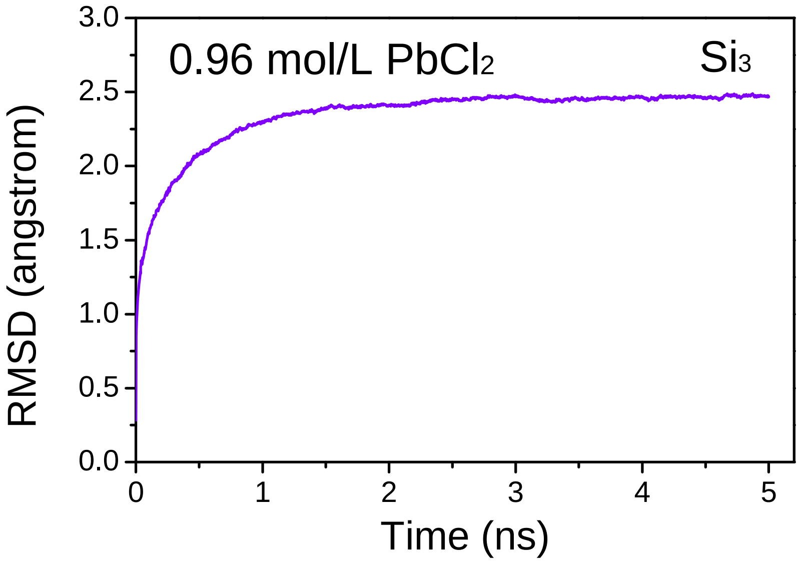
0.96 mol/L PbCl2
Si3
RMSD (angstrom)
Time (ns)
3.0
2.5
2.0
1.5
1.0
0.5
0.0
0
1
2
3
4
5

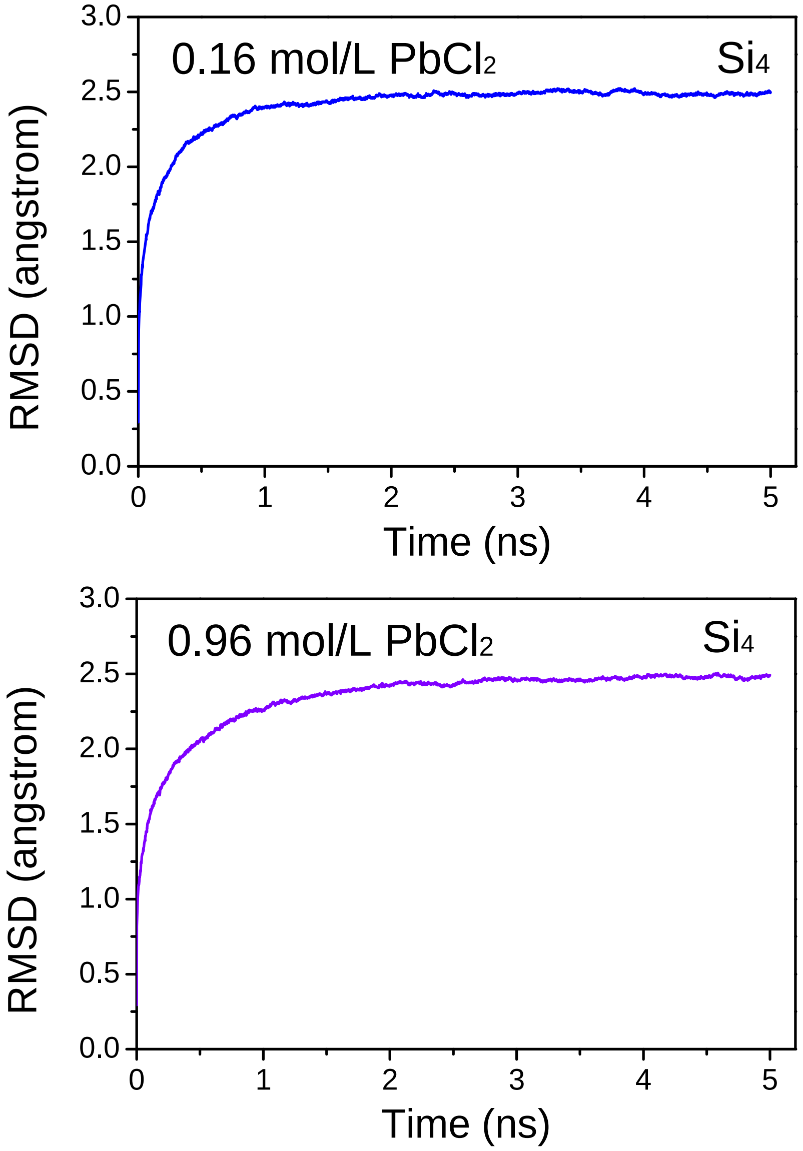

Figure S12. RMSD plots for MD simulations of defective kaolinite (**Si_3** and **Si_4**) in contact with 0.16 and 0.96 mol/L $PbCl_2$ solutions.

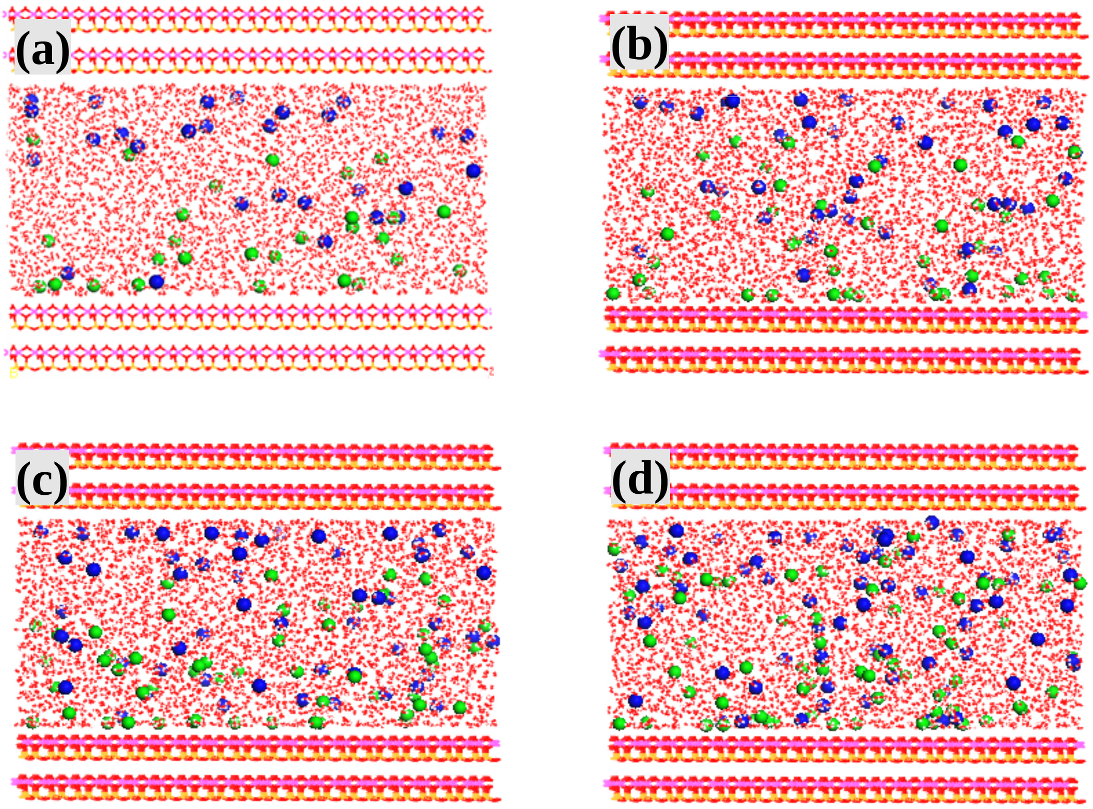

Figure S13. Configurations of regular kaolinite in equilibrium with (a) 0.32 mol/L, (b) 0.48 mol/L, (c) 0.64 mol/L and (d) 0.80 mol/L NaCl solutions.

Na^+ and Cl^- ions are represented as blue and green balls, respectively.

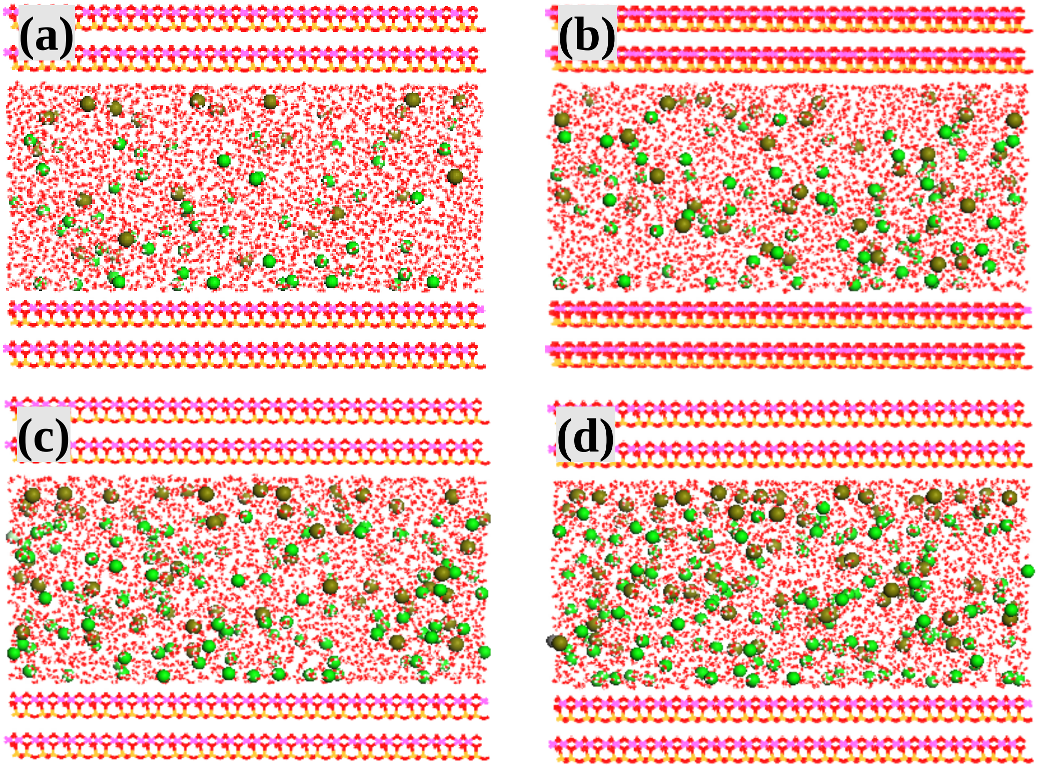

Figure S14. Configurations of regular kaolinite (**Si_0**) in equilibrium with (a) 0.32 mol/L, (b) 0.48 mol/L, (c) 0.64 mol/L and (d) 0.80 mol/L $PbCl_2$ solutions.
Pb^{2+} and Cl^- ions are represented as dark yellow and green balls, respectively.

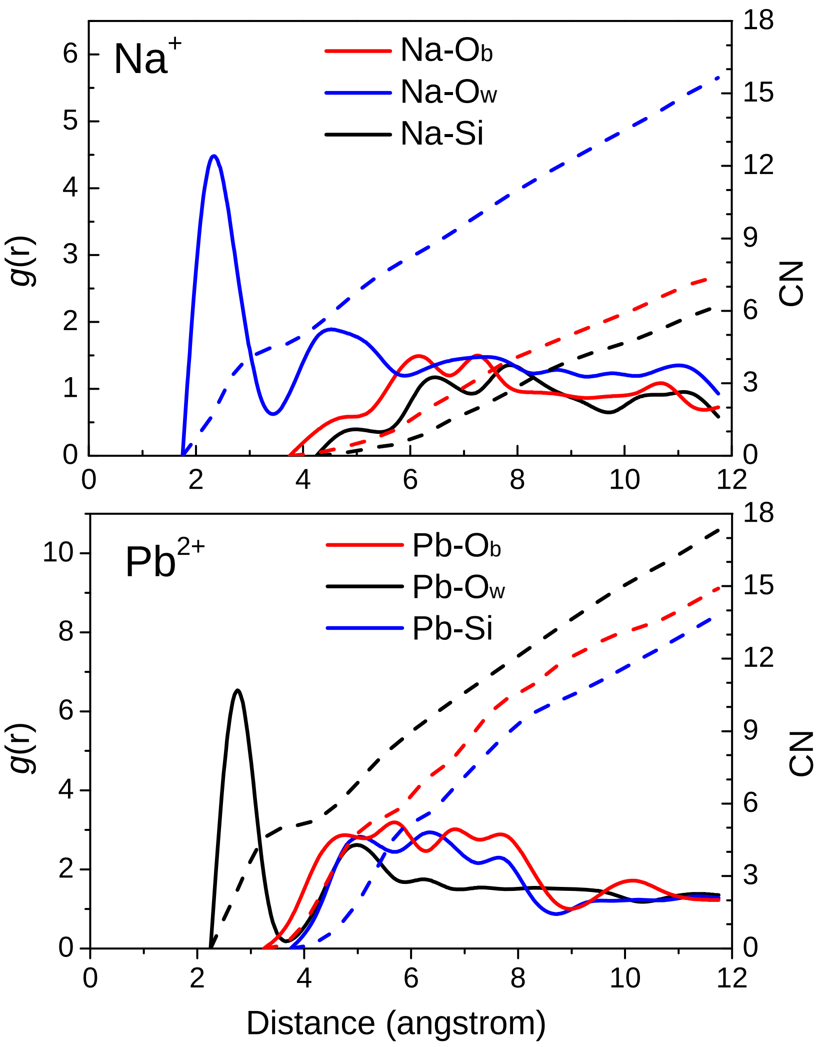

Figure S15. Radial distribution functions (*g*(r)) and coordination numbers (CN) for Na^+/Pb^{2+} adsorption from 0.16 mol/L NaCl and $PbCl_2$ solutions onto the tetrahedral SiO_4 surfaces of regular kaolinite.

Plots of coordination numbers (CN) are in dashed lines.

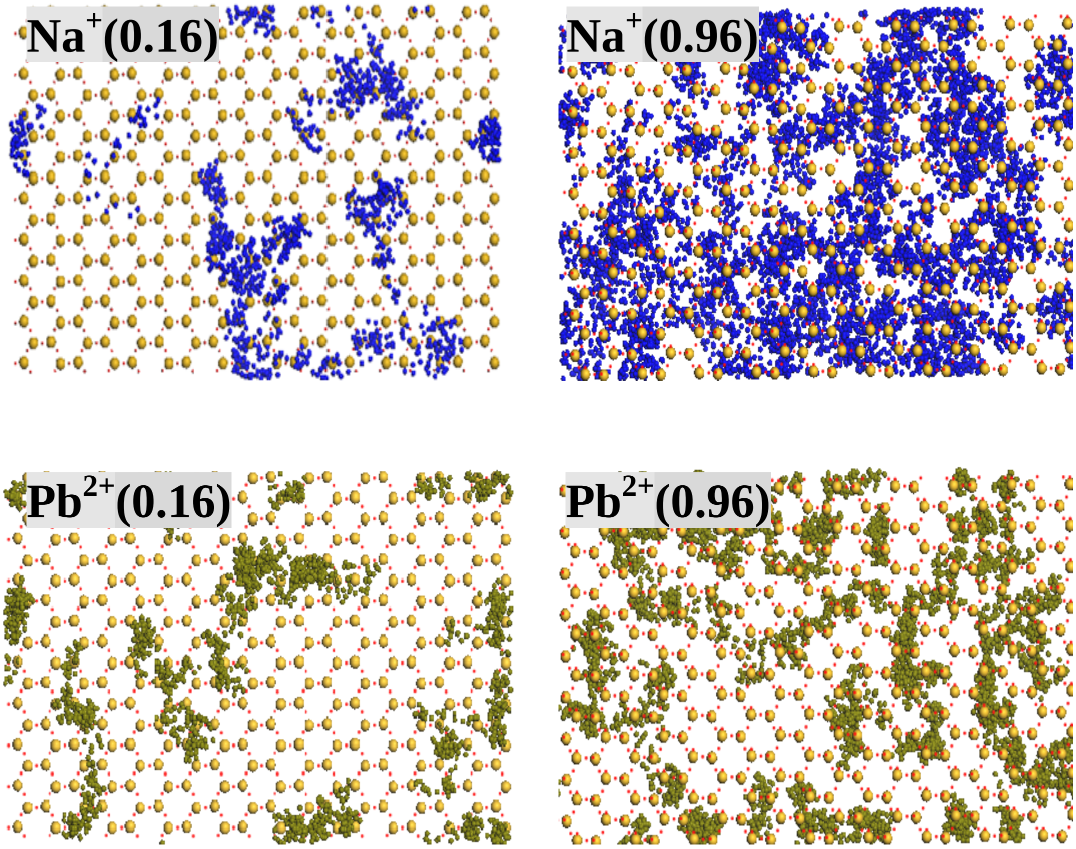

Figure S16. Trajectory maps of the tetrahedral SiO_4 surfaces of regular kaolinite in contact with 0.16 and 0.96 mol/L NaCl/$PbCl_2$ solutions.

Only the inner- and outer-sphere metal ions are made visible, and Na^+ and Pb^{2+} ions are represented as blue, dark yellow, respectively.

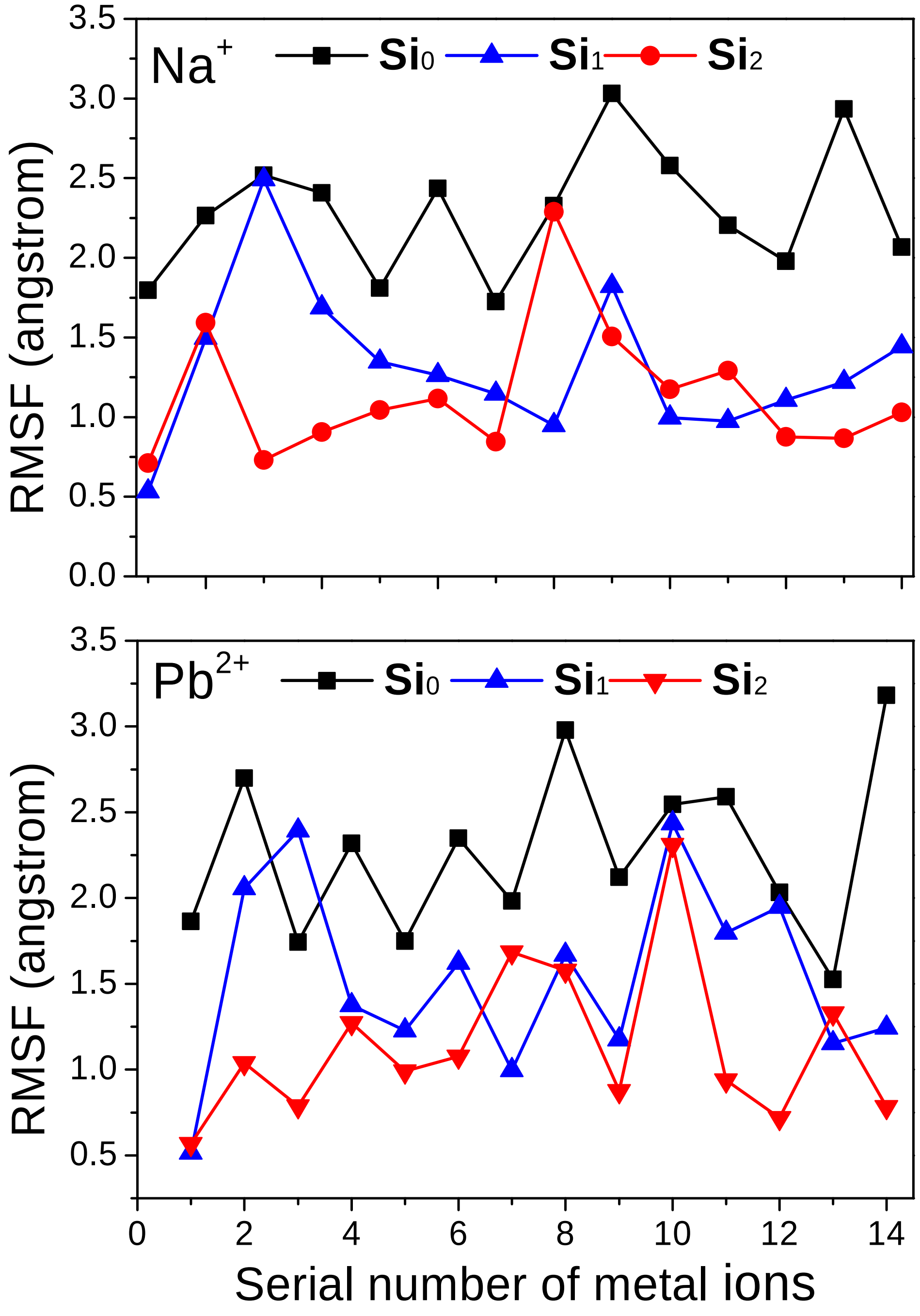

Figure S17. RMSF plots for Na^+/Pb^{2+} for kaolinite surfaces in contact with 0.16 mol/L NaCl/$PbCl_2$ solutions over the 5.0 ns MD simulations.

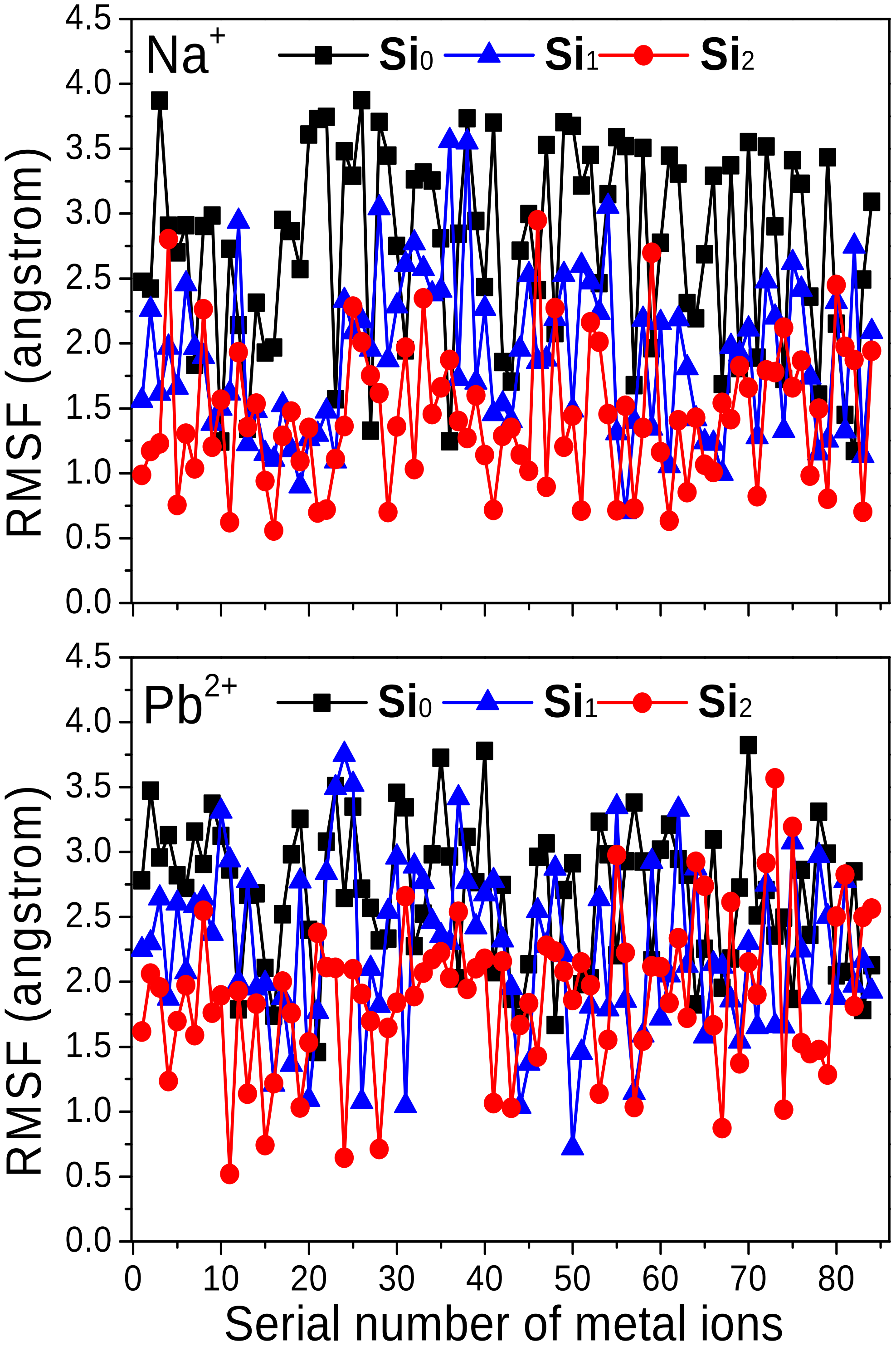

Figure S18. RMSF plots for Na^+/Pb^{2+} for kaolinite surfaces in contact with 0.96 mol/L $NaCl/PbCl_2$ solutions over the 5.0 ns MD simulations.

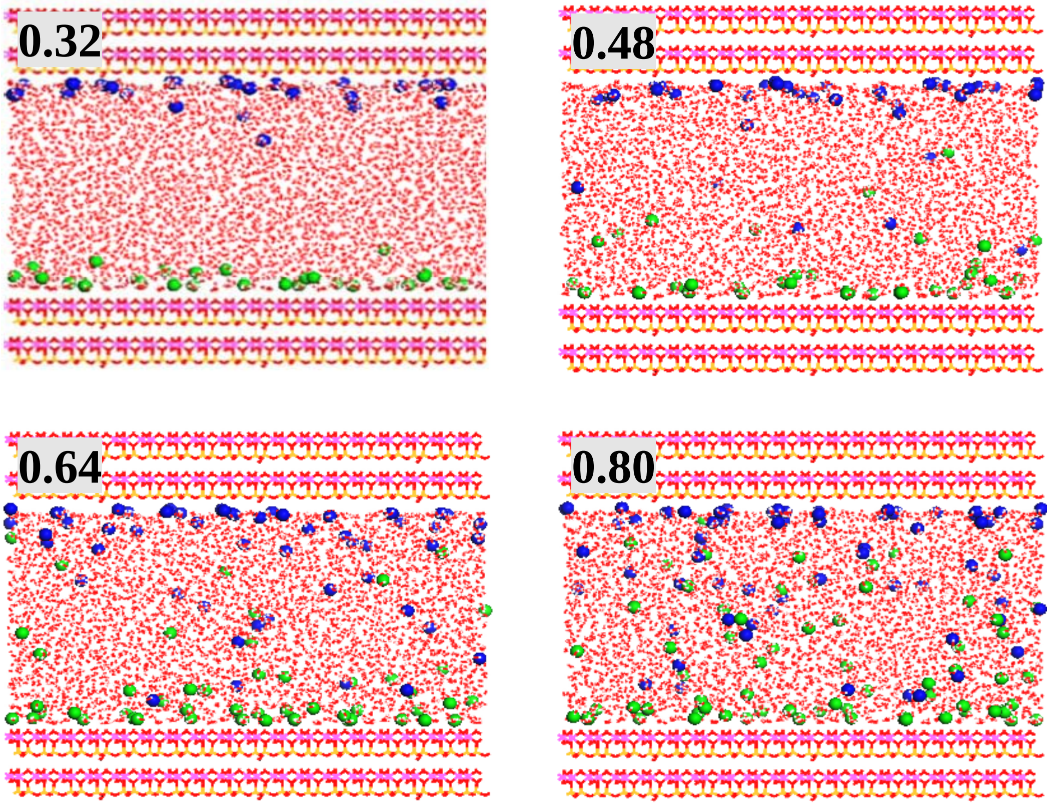

Figure S19. Configurations of defective kaolinite (**Si_1**) in equilibrium with NaCl solutions of different concentrations.

The concentrations (mol/L) of NaCl solutions are indicated in the legends. Na^+, Pb^{2+} and Cl^- ions are represented as blue, dark yellow and green balls, respectively.

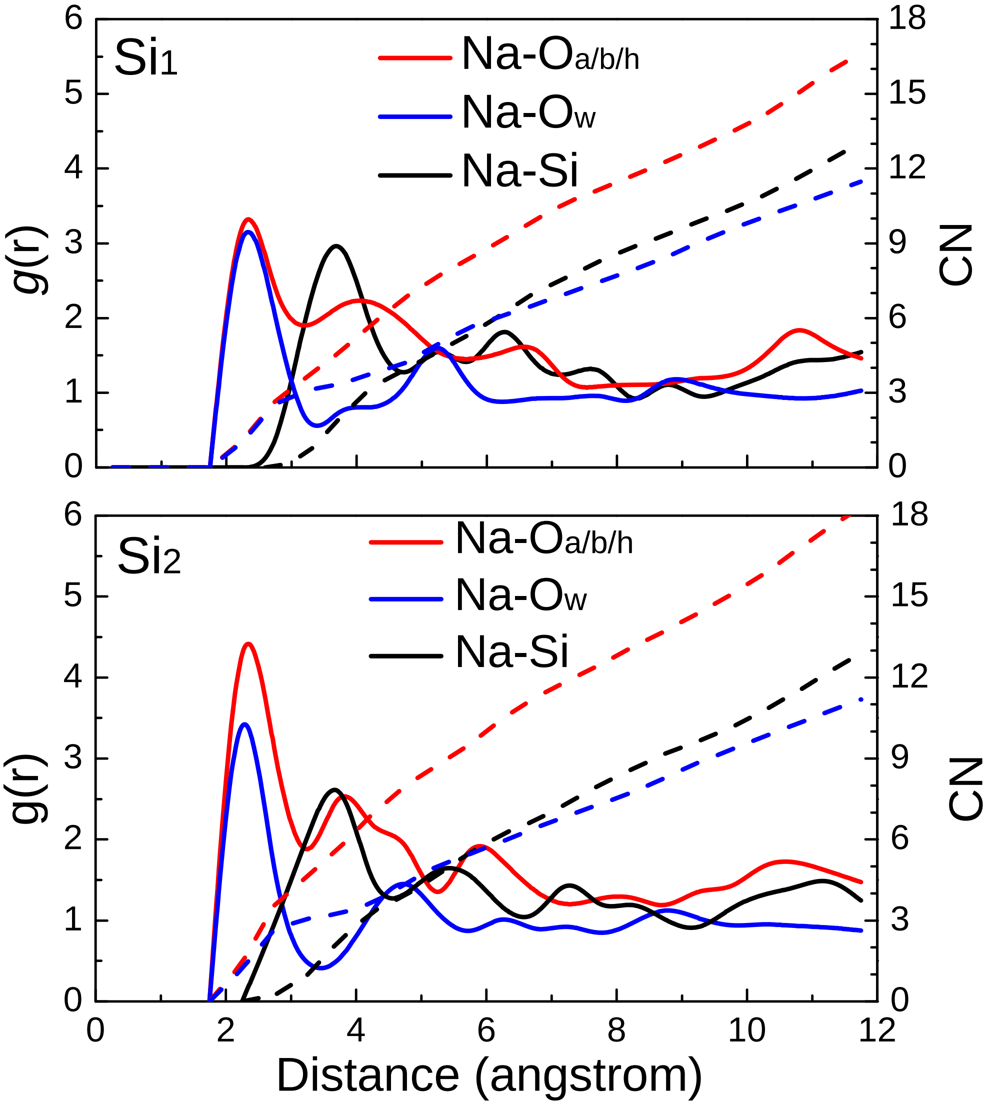

Figure S20. Radial distribution functions (g(r)) and coordination numbers (CN) for Na^+ adsorption from 0.16 mol/L NaCl solutions onto the tetrahedral SiO_4 surfaces of defective kaolinite (**Si_1** and **Si_2**).

Plots of coordination numbers (CN) are in dashed lines.

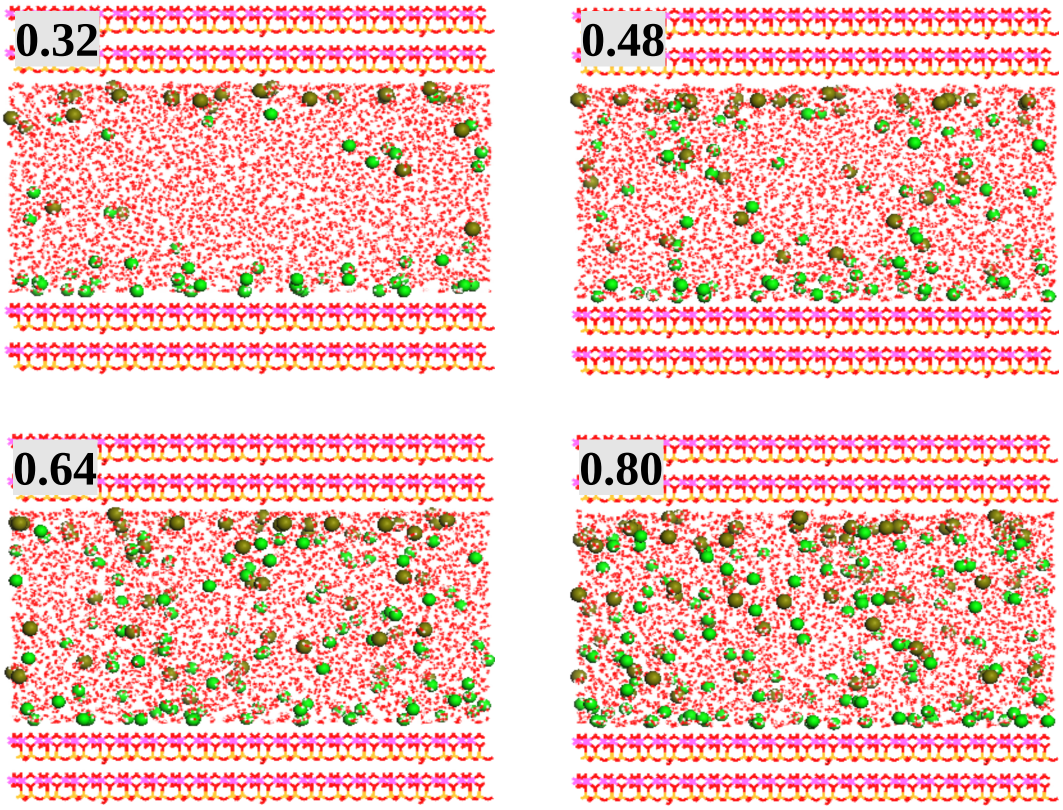

Figure S21. Configurations of defective kaolinite (**Si$_1$**) in equilibrium with $PbCl_2$ solutions of different concentrations.

The concentrations (mol/L) of NaCl solutions are indicated in the legends. Pb^{2+} and Cl^- ions are represented as dark yellow and green balls, respectively.

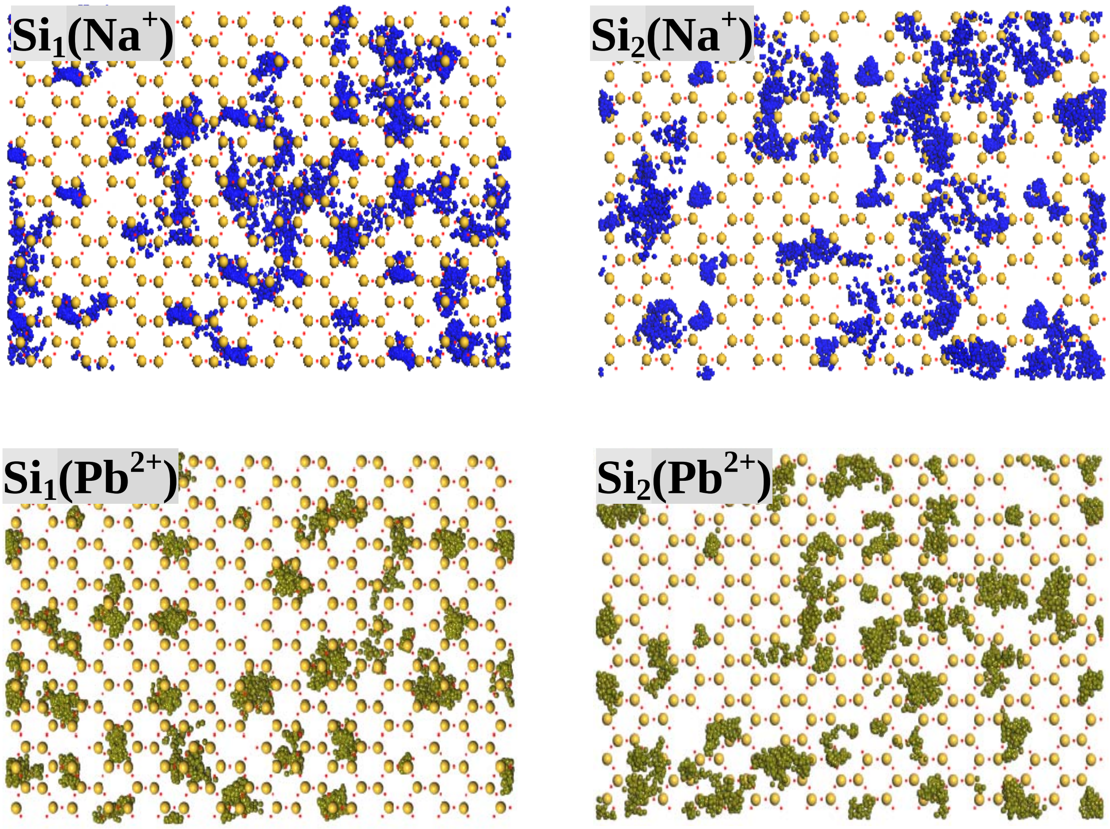

Figure S22. Trajectory maps of the tetrahedral SiO_4 surfaces of defective kaolinite (**Si1** and **Si2**) in contact with 0.96 mol/L NaCl and $PbCl_2$ solutions.

Only the inner- and outer-sphere metal ions are made visible. Na^+, Pb^{2+} ions are represented as blue, dark yellow, respectively.

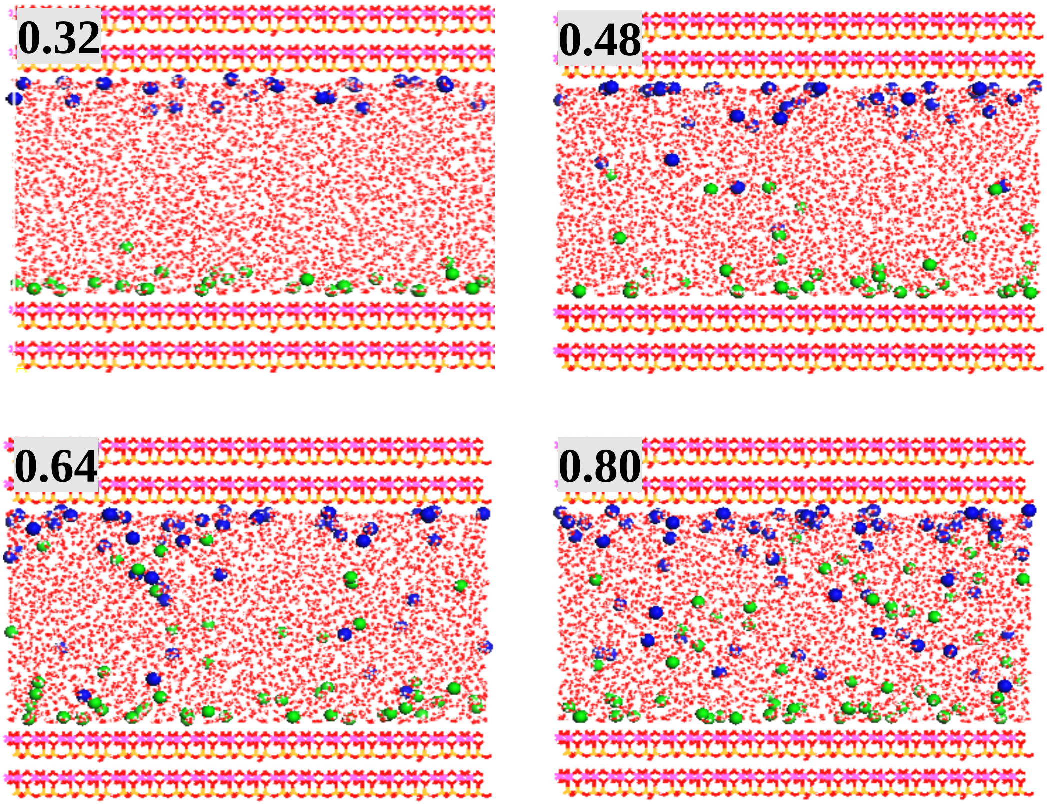

Figure S23. Configurations of defective kaolinite (**Si_2**) in equilibrium with NaCl solutions of different concentrations.

The concentrations (mol/L) of NaCl solutions are indicated in the legends. Na^+ and Cl^- ions are represented as blue and green balls, respectively.

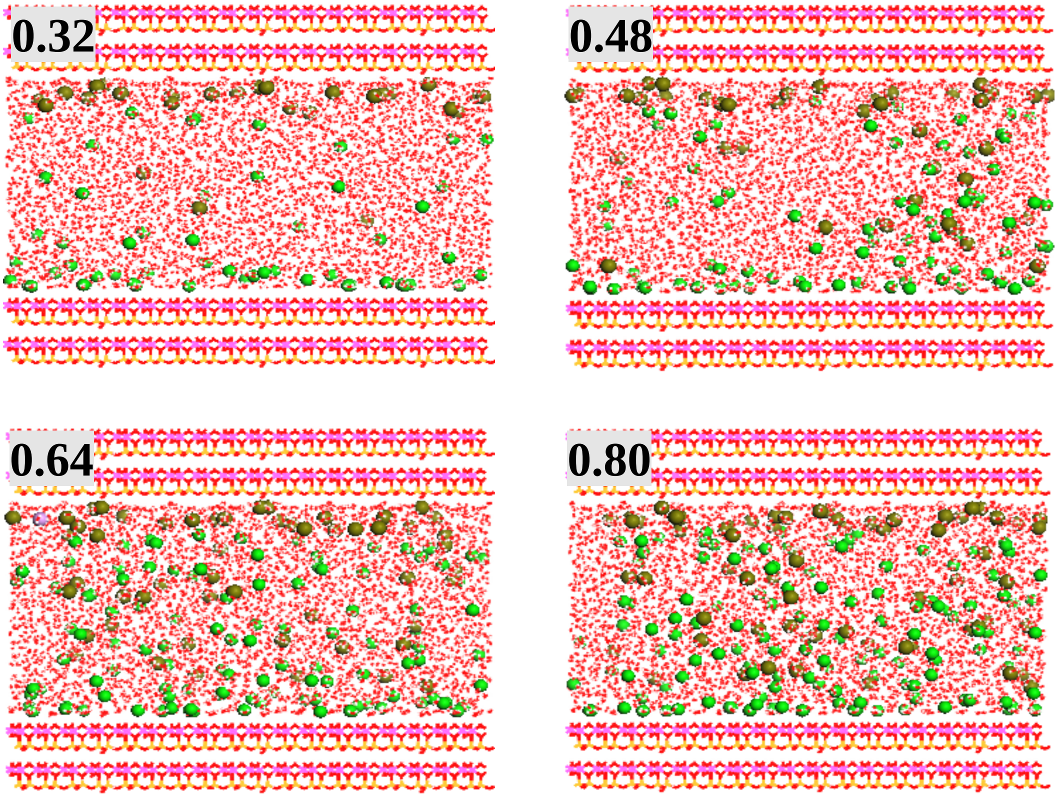

Figure S24. Configurations of defective kaolinite (**Si_2**) in equilibrium with $PbCl_2$ solutions of different concentrations.

The concentrations (mol/L) of NaCl solutions are indicated in the legends. Pb^{2+} and Cl^- ions are represented as dark yellow and green balls, respectively.

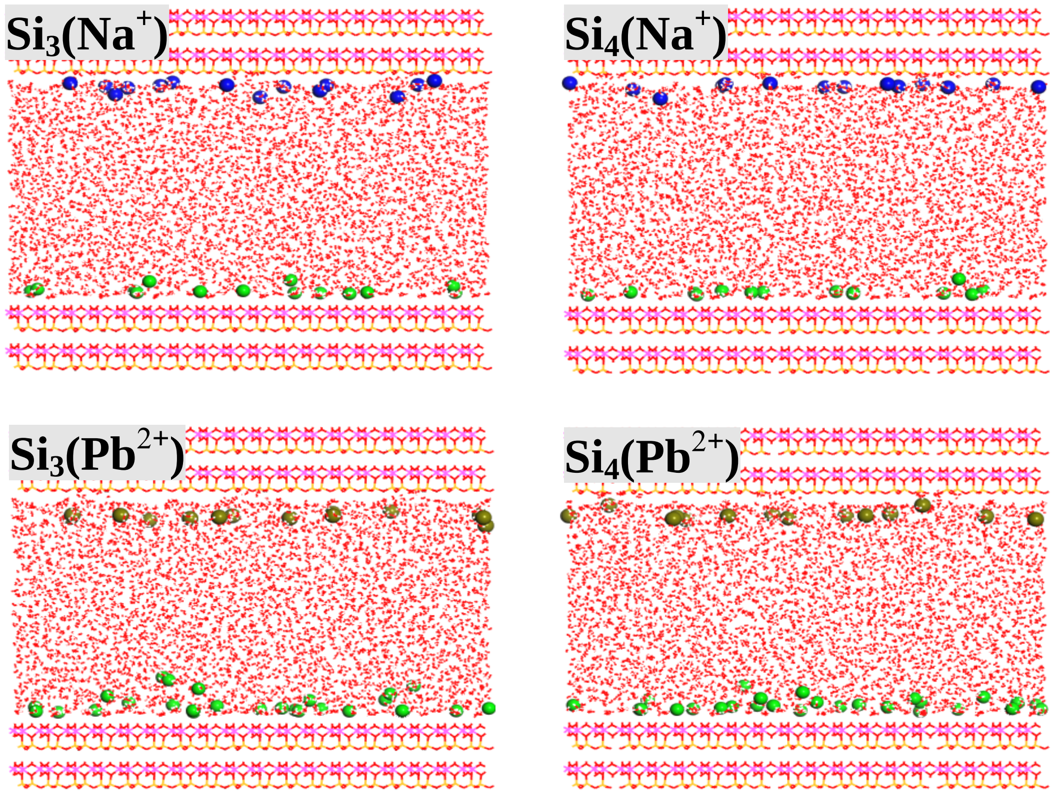

Figure S25. Configurations of kaolinite with **Si_3** and **Si_4** defect sites in equilibrium with 0.16 mol/L NaCl and $PbCl_2$ solutions.

Na^+, Pb^{2+} and Cl^- ions are represented as blue, dark yellow and green balls, respectively.

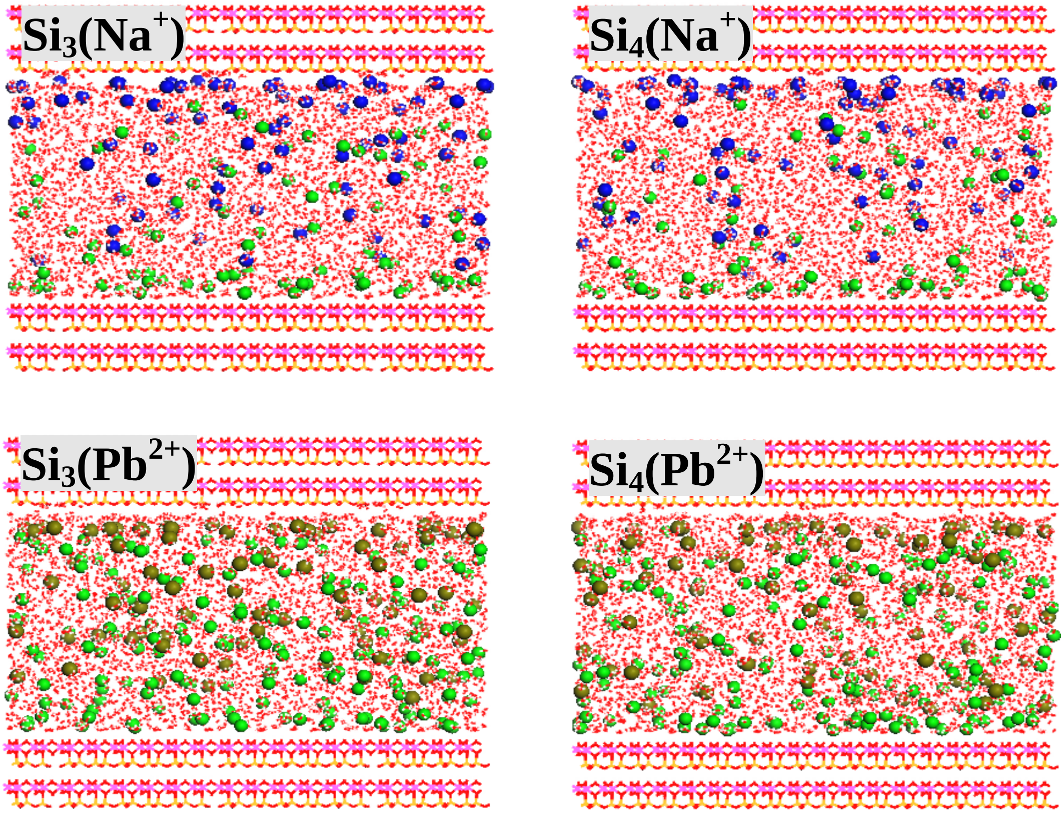

Figure S26. Configurations of kaolinite with **Si_3** and **Si_4** defect sites in equilibrium with 0.96 mol/L NaCl and $PbCl_2$ solutions.

Na^+, Pb^{2+} and Cl^- ions are represented as blue, dark yellow and green balls, respectively.

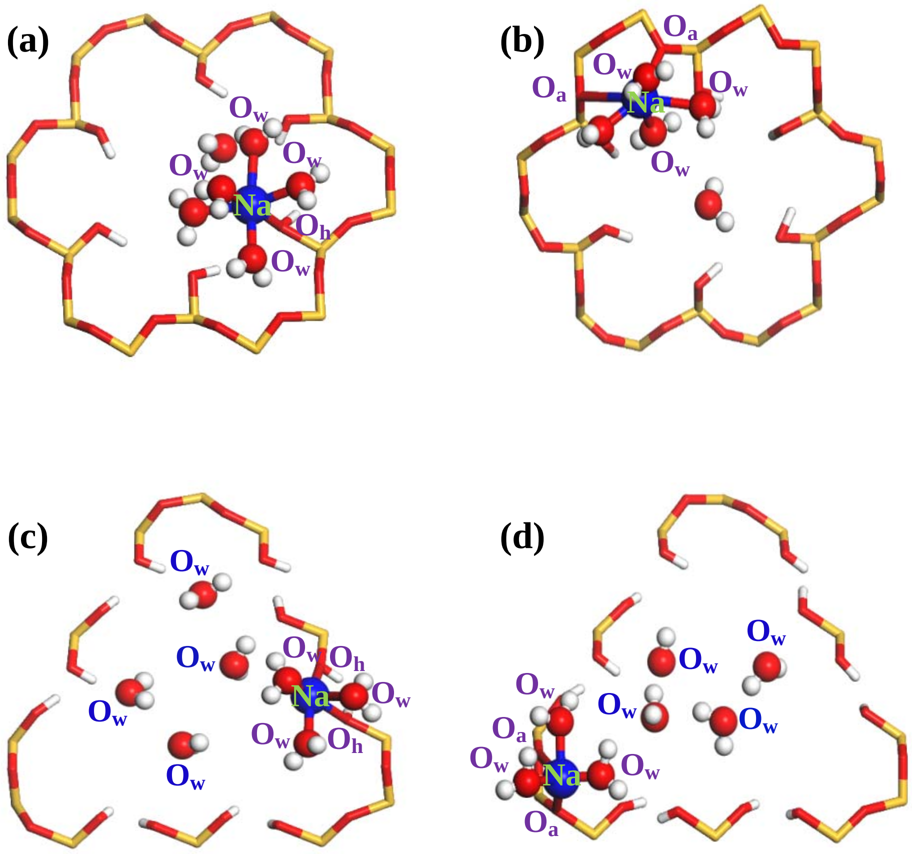

Figure S27. I Local structures of inner-sphere Na^+ ions adsorbed on the tetrahedral SiO_4 surfaces of defective kaolinite (**Si_3** and **Si_4**).

(a), (b) are for **Si_3** and (c), (d) for **Si_4**, respectively. Note that Figure 3a is the third interaction structure for these two defect sites (**Si_3** and **Si_4**) as in the cases of **Si_1** and **Si_2**.

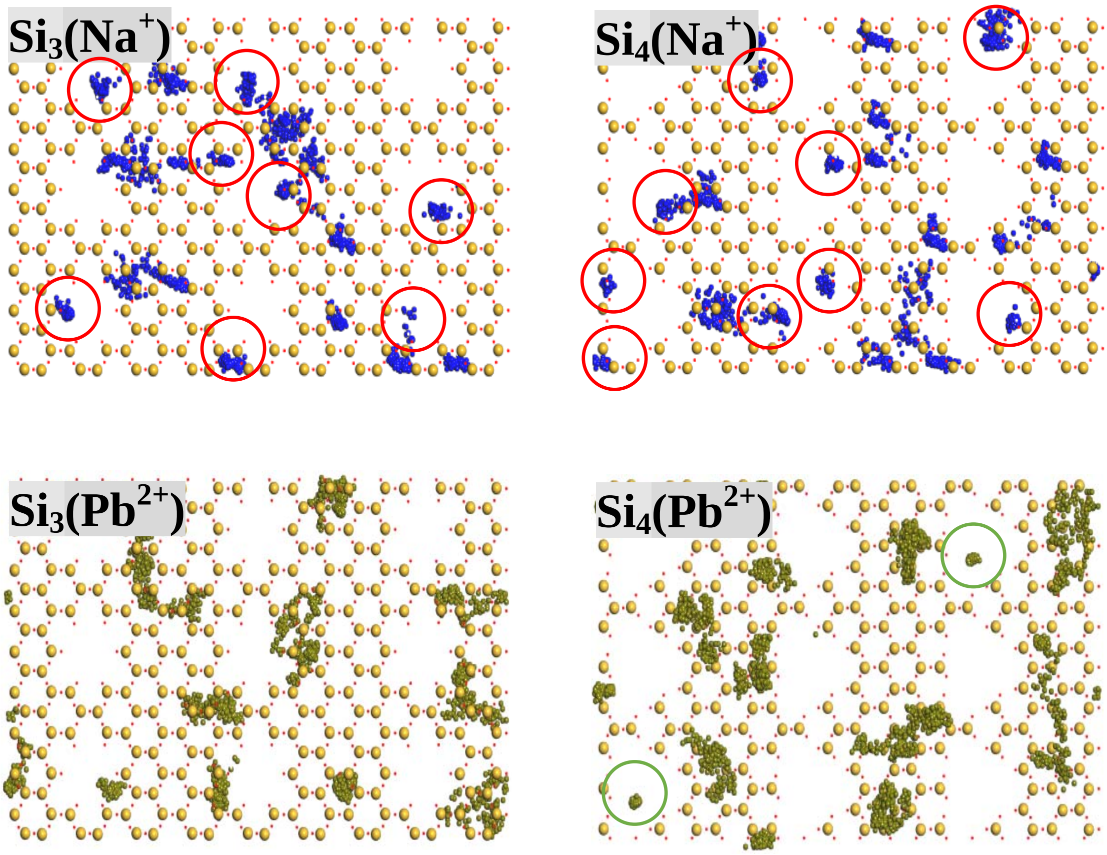

Figure S28. Trajectory maps of the tetrahedral SiO_4 surfaces of defective kaolinite (**Si_3** and **Si_4**) in contact with 0.16 mol/L NaCl and $PbCl_2$ solutions.

Only the inner- and outer-sphere metal ions are made visible, and Na^+ and Pb^{2+} ions are represented as blue and dark yellow balls, respectively. Inner Na^+ ions that fall around the silanol nests are highlighted by red circles, and two quasi inner-sphere Pb^{2+} ions are highlighted by green circles.

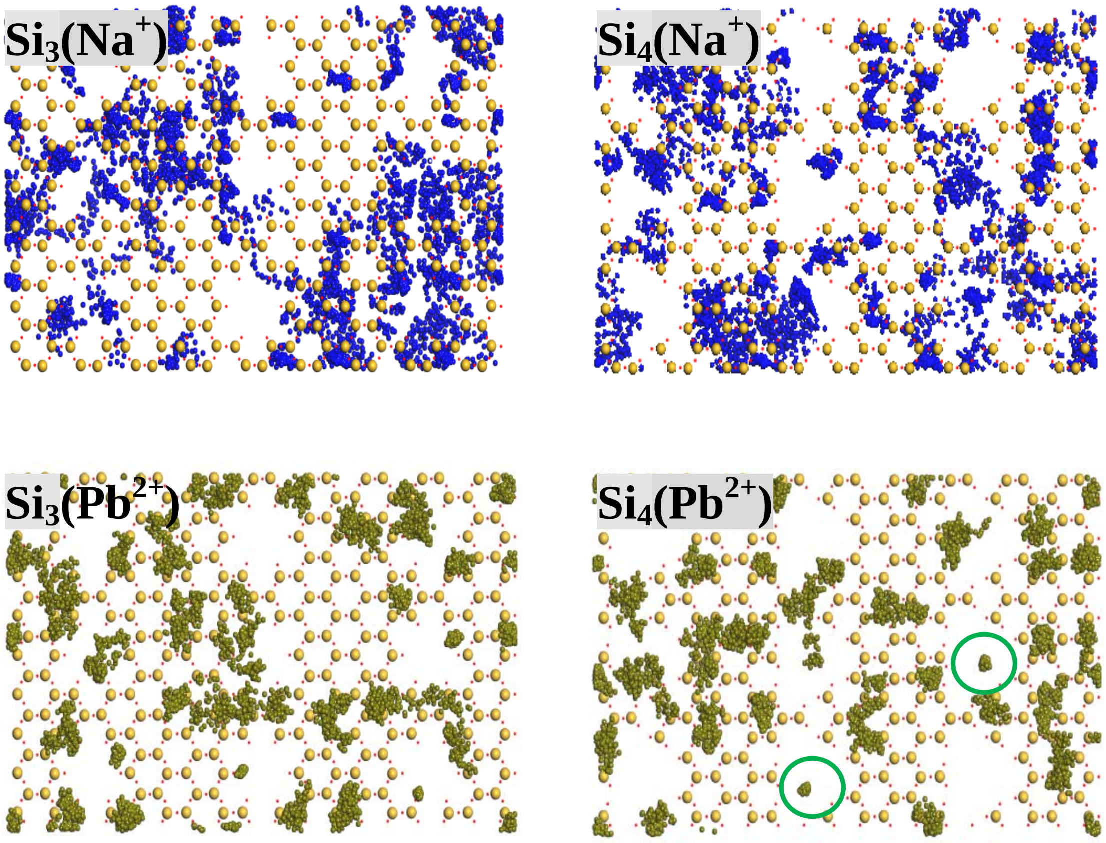

Figure S29. Trajectory maps of the tetrahedral SiO_4 surfaces of defective kaolinite (**Si_3** and **Si_4**) in contact with 0.96 mol/L NaCl and $PbCl_2$ solutions.

Only the inner- and outer-sphere metal ions are made visible, and Na^+ and Pb^{2+} ions are represented as blue and dark yellow balls, respectively. The two quasi inner-sphere Pb^{2+} ions are highlighted by green circles.

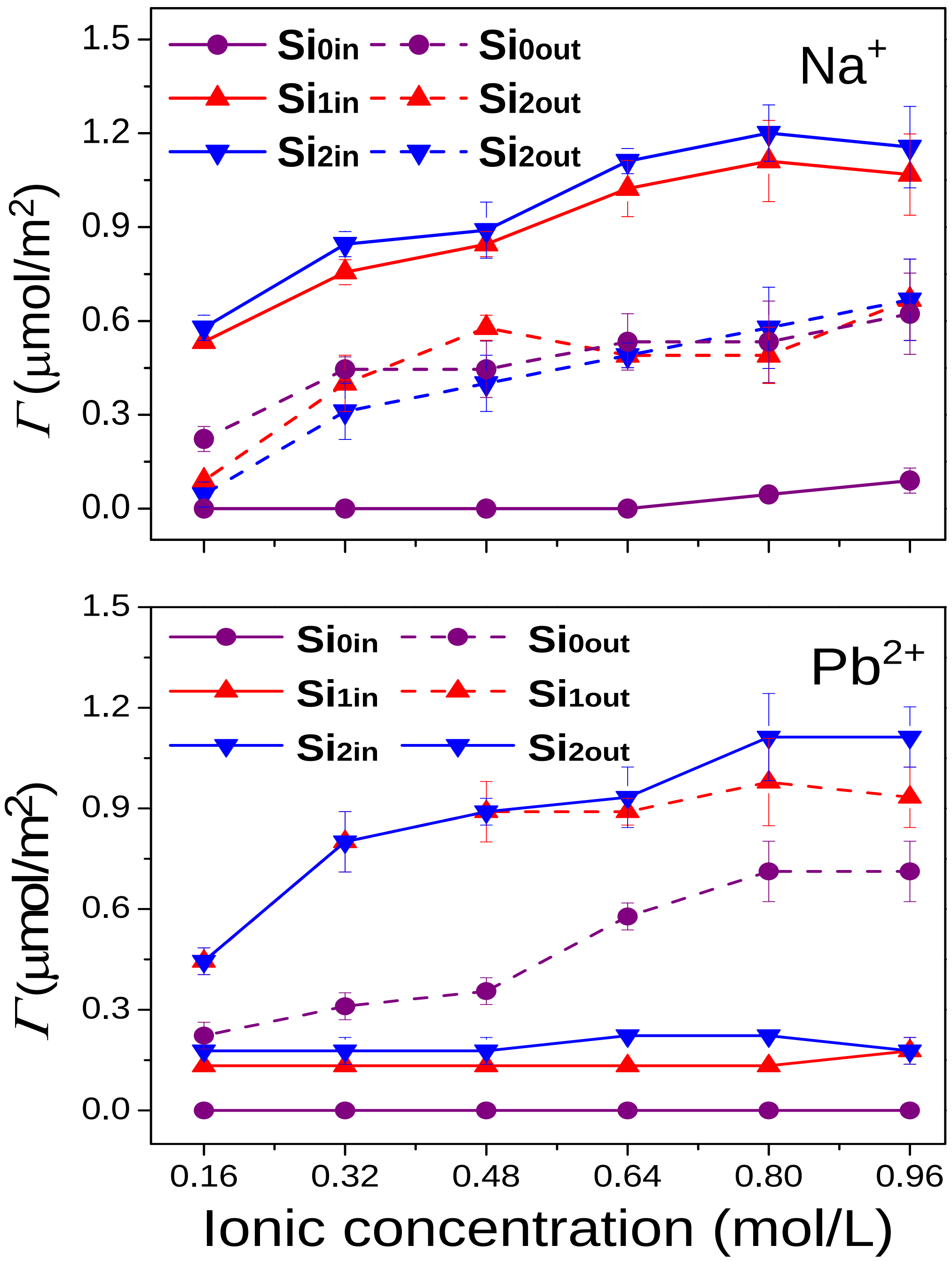

Figure S30. Adsorption capacities (Γ) of Na^{+}/Pb^{2+} ions from $NaCl$/$PbCl_2$ solutions with a wide range of concentrations onto the tetrahedral SiO_4 surfaces of kaolinite. Inner- and outer-sphere adsorption modes are differed by solid and dashed lines, respectively.

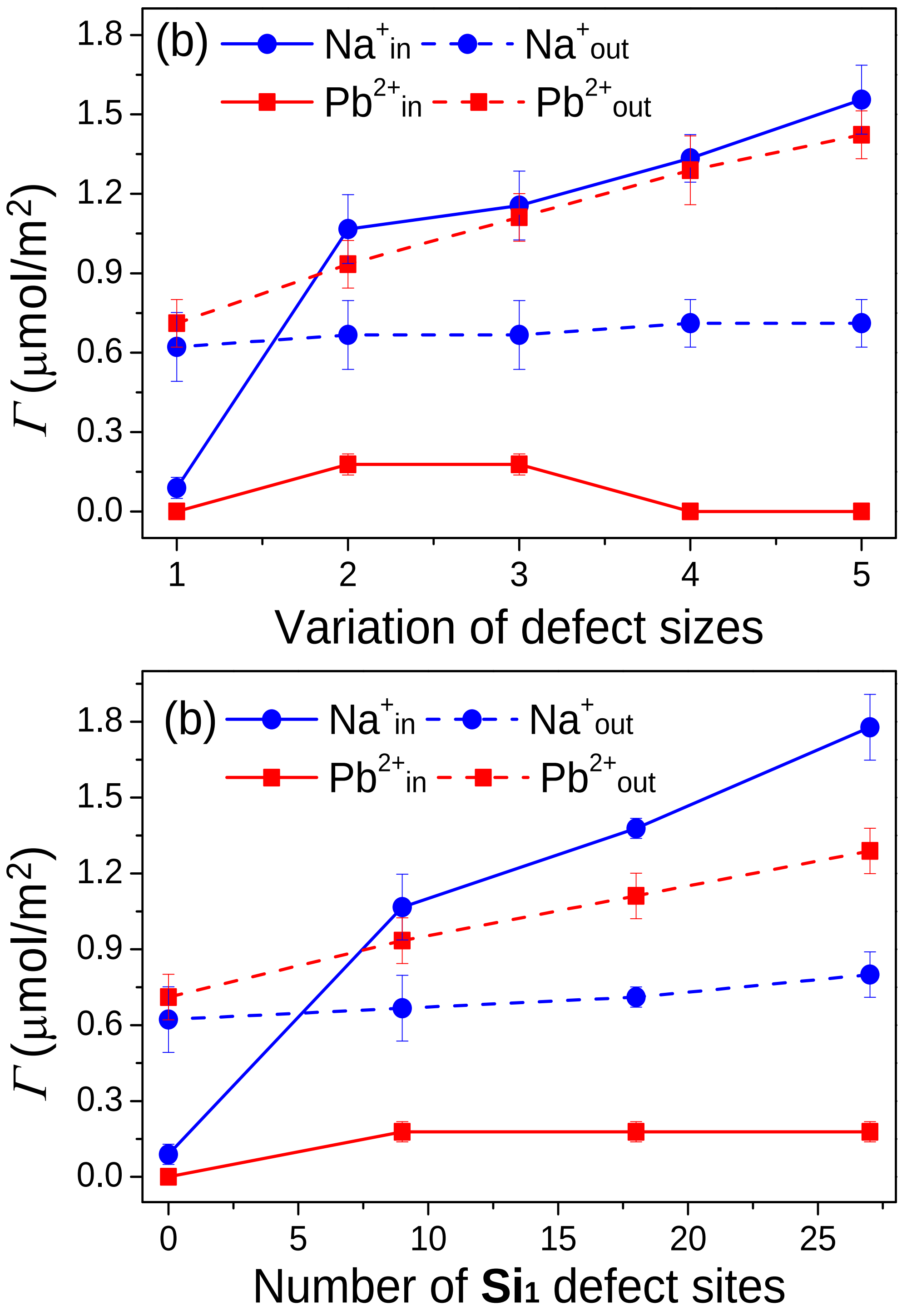

Figure S31. Effects of (a) the size of defect sites and (b) the content of **Si$_1$** defect site on adsorption capacities (Γ) of Na^+/Pb^{2+} ions from 0.96 mol/L NaCl and $PbCl_2$ solutions on the tetrahedral SiO_4 surface of kaolinite.

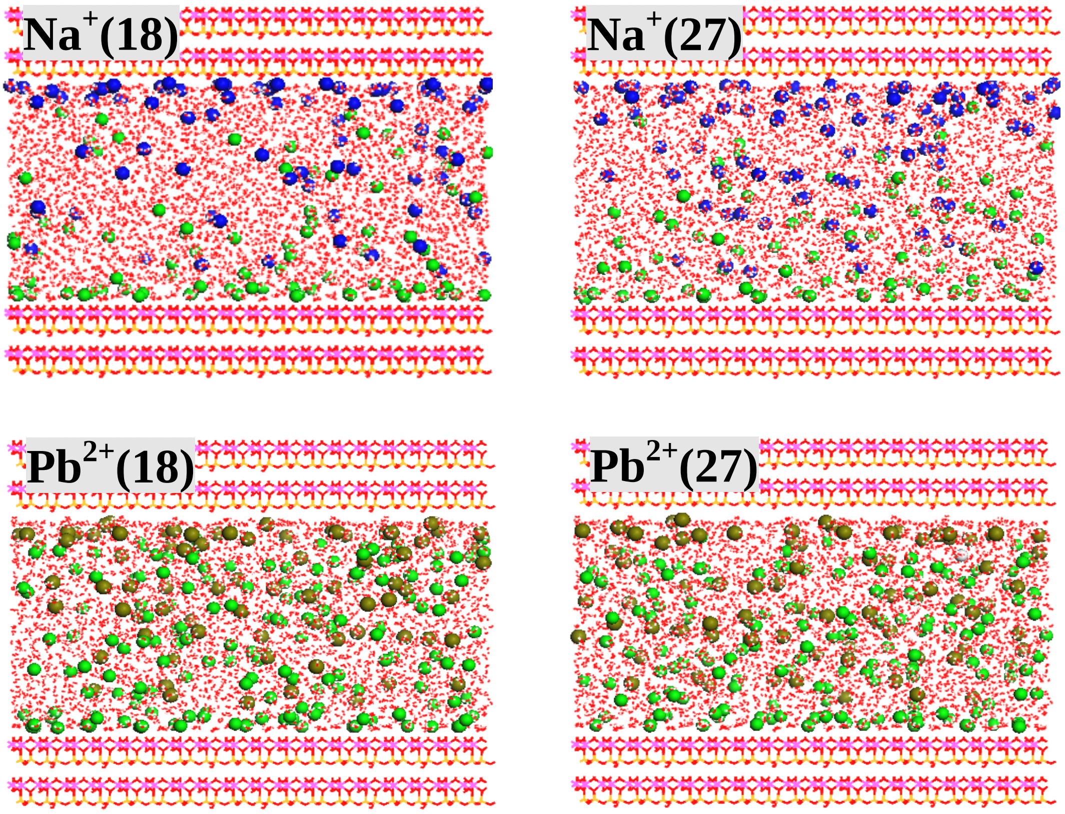

Figure S32. Configurations of kaolinite with different contents of **Si_1** defect site in equilibrium with 0.96 mol/L NaCl and $PbCl_2$ solutions.

The numbers of **Si_1** defect site are indicated in the legends. Na^+, Pb^{2+}, and Cl^- ions are represented as blue, dark yellow and green balls, respectively.
